# Prioritizing multiple therapeutic targets in parallel using automated DNA-encoded library screening

**DOI:** 10.1038/ncomms16081

**Published:** 2017-07-17

**Authors:** Carl A. Machutta, Christopher S. Kollmann, Kenneth E. Lind, Xiaopeng Bai, Pan F. Chan, Jianzhong Huang, Lluis Ballell, Svetlana Belyanskaya, Gurdyal S. Besra, David Barros-Aguirre, Robert H. Bates, Paolo A. Centrella, Sandy S. Chang, Jing Chai, Anthony E. Choudhry, Aaron Coffin, Christopher P. Davie, Hongfeng Deng, Jianghe Deng, Yun Ding, Jason W. Dodson, David T. Fosbenner, Enoch N. Gao, Taylor L. Graham, Todd L. Graybill, Karen Ingraham, Walter P. Johnson, Bryan W. King, Christopher R. Kwiatkowski, Joël Lelièvre, Yue Li, Xiaorong Liu, Quinn Lu, Ruth Lehr, Alfonso Mendoza-Losana, John Martin, Lynn McCloskey, Patti McCormick, Heather P. O’Keefe, Thomas O’Keeffe, Christina Pao, Christopher B. Phelps, Hongwei Qi, Keith Rafferty, Genaro S. Scavello, Matt S. Steiginga, Flora S. Sundersingh, Sharon M. Sweitzer, Lawrence M. Szewczuk, Amy Taylor, May Fern Toh, Juan Wang, Minghui Wang, Devan J. Wilkins, Bing Xia, Gang Yao, Jean Zhang, Jingye Zhou, Christine P. Donahue, Jeffrey A. Messer, David Holmes, Christopher C. Arico-Muendel, Andrew J. Pope, Jeffrey W. Gross, Ghotas Evindar

**Affiliations:** 1 GlaxoSmithKline, 1250 South Collegeville Road, Collegeville, Pennsylvania 19426, USA; 2 GlaxoSmithKline, 830 Winter Street, Waltham, Massachusetts 02451, USA; 3 GlaxoSmithKline, Severo Ochoa 2, Tres Cantos, Madrid 28760, Spain; 4 University of Birmingham, School of Biosciences, Edgbaston, Birmingham B15 2TT, UK

## Abstract

The identification and prioritization of chemically tractable therapeutic targets is a significant challenge in the discovery of new medicines. We have developed a novel method that rapidly screens multiple proteins in parallel using DNA-encoded library technology (ELT). Initial efforts were focused on the efficient discovery of antibacterial leads against 119 targets from *Acinetobacter baumannii* and *Staphylococcus aureus*. The success of this effort led to the hypothesis that the relative number of ELT binders alone could be used to assess the ligandability of large sets of proteins. This concept was further explored by screening 42 targets from *Mycobacterium tuberculosis*. Active chemical series for six targets from our initial effort as well as three chemotypes for DHFR from *M. tuberculosis* are reported. The findings demonstrate that parallel ELT selections can be used to assess ligandability and highlight opportunities for successful lead and tool discovery.

## Abstract (web summary)

Encoded Library Technology (ELT) has streamlined the identification of chemical ligands for protein targets in drug discovery. Here, the authors optimize the ELT approach to screen multiple proteins in parallel and identify promising targets and antibacterial compounds for *S. aureus*, *A. baumannii* and *M. tuberculosis*.

Drug discovery and development is an extremely challenging, lengthy and expensive endeavour with an unsustainable success rate^1^,^2^. Understanding the ligandability of a target protein remains a central challenge for early small molecule drug discovery programmes^3^. An integrated lead discovery approach often employs biochemical target-based screening, cellular screening, phenotypic screening or a combination of methods. In most cases, a biochemical target-based approach is chosen to search for early lead molecules^4^. This approach requires extensive reagent generation, assay development, lead identification and optimization efforts, and can amount to years of invested time and millions of dollars in expense. The approach only interrogates a tiny fraction of the essential and druggable proteome and often results in failure after considerable investment. The alternative phenotypic and cellular screening methods probe multiple targets but often require target deconvolution efforts to support lead optimization as well as significant resource and technology investments to execute^5^,^6^. The antibacterial therapeutic area is particularly challenging, with the number of approved drugs steadily declining since 1980. Multiple factors have contributed to the lack of success, including the emergence of resistance, challenges in designing cell penetration properties into an antibacterial agent, a focus on genes essential for growth in rich media (only 7% in *Escherichia coli*) and a general lack of tools to understand the complex biology of host–pathogen interactions^7^,^8^,^9^. In addition to the many uncharacterized bacterial targets, 75% of protein research into human targets still focuses on only 10% of the proteins known before the human genome mapping^10^. A platform to prioritize this wider scope of targets based on ligandability and the rapid identification of lead and tool molecules for these proteins will aid in a deeper understanding of biology and the discovery of the next generation of therapeutics.

A variety of academic and pharmaceutical research has been directed towards addressing the need for understanding chemical tractability over the past decade and both experimental and computational strategies have been reported^11^,^12^,^13^. As described elsewhere, chemical tractability or ligandability is the ability of a protein to bind chemical matter, whereas druggability describes the possibility of discovering a molecule for the target that will modulate the disease state^3^. A technique to characterize small molecule ‘hot spots’ on proteins was reported by Hajduk *et al*.^14^, where the relationship between ligandability and fragment-binding measurements with NMR was described. Edfeldt’s more recent feature highlights the use of fragment technology to probe the ligandability of 36 targets in the AstraZeneca portfolio over an 8-year period^15^. Computational methods and docking approaches have been reported and are highly valueable^16^,^17^. These techniques have helped guide target selection for the past decade but are often limited by target throughput, experimental efficiency and the prerequisite that structural information is available.

A relatively recent addition to drug discovery screening is the use of DNA-encoded chemical libraries (DEL) or encoded library technology (ELT) to discover small molecule binders to targets of interest^18^. First conceptualized in 1992 (ref. 19), various methods for creating large DNA-encoded combinatorial libraries have been developed, such as split and pool encoding^20^, DNA-templated synthesis^21^,^22^ and self-assembling libraries^23^,^24^. Regardless of the library synthesis technique, binders to individual proteins are selected by affinity from complex mixtures. Binding events are detected by harnessing the power of high-throughput sequencing to identify DNA tags encoding the bound chemotypes. Chemotypes of interest can then be re-synthesized off-DNA for assay testing and further development. The technology has become widely used throughout the pharmaceutical, biotech and academic environments to discover ligands and is continually evolving^25^,^26^,^27^. ELT has been employed successfully to identify numerous molecules that bind to proteins from diverse target classes and sample deep chemical space as well as understand structure activity relationships directly from the screening output^28^,^29^,^30^,^31^,^32^,^33^,^34^,^35^,^36^,^37^,^38^.

At GlaxoSmithKline (GSK), we have created a DNA-encoded compound collection containing over 100 different libraries comprised of billions of unique molecules generated from over 40 reaction types using a split and pool encoding strategy^39^,^40^. These libraries are ultimately assembled and screened in a single pool, allowing for the rapid identification of chemical ligands. The technology has continued to mature and ELT is now considered for every small molecule screening effort in the GSK portfolio to discover new chemical matter. As high-throughput sequencing has continued to advance and we have refined our analysis methods, the target capacity of the technology has increased. Pairing these improvements with the automation of protein immobilization and affinity selections has allowed for the screening of multiple targets in a single experiment, as opposed to probing one target at a time, enabling a panel to be executed in a 5–7-month period^41^. Thus, the interrogation of a larger fraction of the proteome is possible while maintaining the simplicity and efficiency of a reductionist target-based approach.

Here we present the successful use of this strategy to identify tractable targets from *Staphylococcus aureus* and *Acinetobacter baumannii* and to discover individual lead/tool molecules for six different target proteins. We describe the chemical series and their activity against each protein. In addition, we propose an evolution of the panel screening, in which the output of the screen moves beyond identifying active pharmacophores and is used to rapidly assess targets based on their ligandability as determined by ELT. The refined approach was used to prioritize proteins from *Mycobacterium tuberculosis* and we present those results along with active pharmacophores against one of the highest-ranked targets as a proof of concept. Our results demonstrate that ELT can inform the allocation of resources within the drug discovery process towards the most chemically tractable targets. We envision the methodology will provide a tool to assess targets associated with virtually any therapeutic area. Parallel screening could include targets clustered in pathways, related targets, or a single target examined under multiple conditions perhaps using multiple constructs^42^,^43^. While this assessment is independent from target selection in the broader biological context, the technique could enable a deeper understanding of disease biology by rapidly providing much-needed tool molecules^18^,^44^,^45^. Here we report data supporting this assertion as well as several novel chemotypes as tools for the antibacterial field.

## Results

### ELT selection outcome

**Figure 1:**
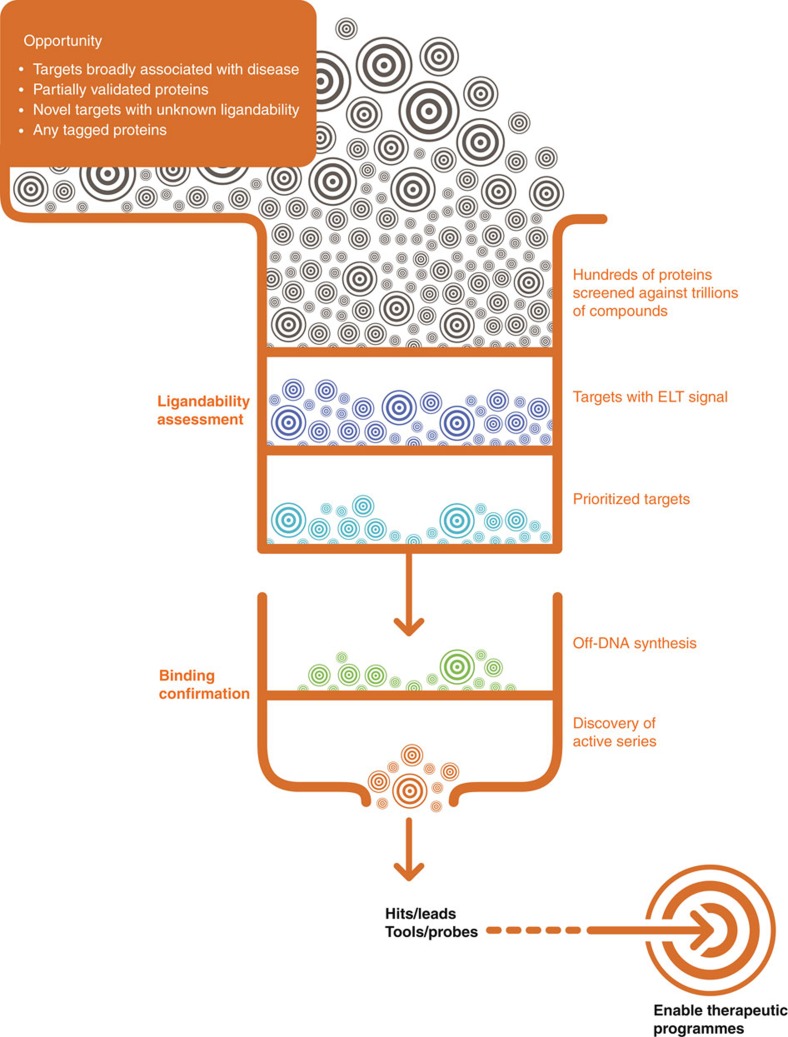
Schematic representation of the ELT screening and ligandability assessment strategy. Hundreds of potential targets of interest are immobilized and screened against GSK's DNA encoded libraries. Targets with signal are ranked by the counts of ELT binders which correlate to the protein's chemical ligandability. Data is used to plan off-DNA synthesis and confirm hits in assays. In addition to novel chemical starting points for lead optimization, the output is a target/chemotype pair that enables therapeutic programs by providing tools for target validation as well as a ranking of targets to prioritize further studies.

**Figure 2:**
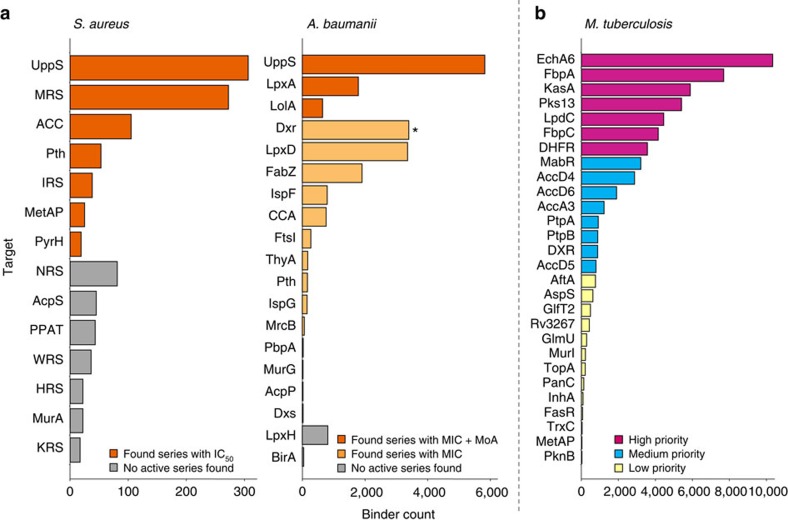
Count of binders that are specific to each target. (**a**) Results of screening against *S. aureus* and *A. baumanii* panels. Targets are binned and coloured by the activity of molecules found for the target and sorted by the number of binders. (**b**) Proposed priority of targets for the *M. tuberculosis* panel. Targets are sorted by binder count and coloured by the proposed priority based on the number of specific binders found. *Compounds for Dxr confirmed by biochemical assay.

A schematic showing the streamlined selection of targets for R&D efforts through the ELT tractability approach is illustrated in Fig. 1. The ELT selections were conducted by immobilizing affinity-tagged protein onto an affinity matrix, then exposing the protein to pooled compound libraries before washing away non-binders and recovering bound compounds by heat elution (detail below). This process was repeated to enrich bound species and reduce the population that does not bind to the protein of interest (described previously)^20^,^31^,^32^,^33^,^37^. The individual selection process was adapted to an automation platform such that hundreds of proteins could be evaluated in parallel. For each selection, final yields of 10^7^–10^9^ sequences of DNA were obtained, quantified using qPCR and amplified for sequencing as described in the ‘Methods’ section and previously^20^. The collected data were translated from the DNA barcode to the associated encoded molecule. On the basis of the library size and the number of sequences obtained, the noise level was calculated for each selection. Signal strength is reported as a value relative to that level (that is, signal value of 10 represents 10-fold greater measurement than noise). All data points with signal greater than two were included in subsequent data analysis steps. This output was then filtered to remove chemotypes that had been identified as binders to affinity matrix or multiple proteins in past selections (non-specific or frequent nuisance binders). This set of specific binders was clustered by chemical similarity (Tanimoto score >0.85) or shared building blocks. The compounds can be used as tools to assess the validity of the target or potentially as a lead molecule. Over the course of these panels, the number of libraries available for ELT screening expanded from 36 in the initial panel to 84 in the final screening panel and is currently over 100. This increase in library size was accompanied by a parallel increase in the reaction types used to create those libraries contributing to a modest increase in diversity and an ability to probe a greater breadth of chemical space. Generally, we assessed the target’s tractability by examining the number of enriched chemical series as illustrated in Fig. 2 by a plot of the number of binders versus target protein. Committing chemistry resource to the most tractable targets first, clusters with dense representation, high signal strength and favourable chemical properties were prioritized for synthesis of representative chemical series^46^. In addition, a structure similarity search (Tanimoto >0.85) was conducted against the corporate collection for every target, including those with fewer binders, enabling rapid hit confirmation as well as interrogation of all targets with signal. Specific outcomes for each of the three panels are described below.

### *S. aureus* panel

The *S. aureus* ELT target panel was a collection of 39 essential enzyme targets. Table 1 shows the high-level progression of the targets in each panel; for detailed target information, see Supplementary Table 1. These targets were selected based on their potential utility as antibacterial drug targets and the availability of a biochemical assay system either inside GSK or rapidly adaptable from reported literature. N- and C-terminal dual-tagged proteins were subjected to ELT selections; one tag was used for rapid protein purification and the second for ELT selection immobilization. Miniaturization, automation and coupling of the purification and ELT selections in a parallel method enabled maximum efficiency (described in detail in Supplementary Methods). Data from the targets were examined individually and chemical series for 14 targets were chosen for follow-up by off-DNA synthesis (Fig. 2a). Approximately five compounds per chemical series and three chemical series for each target were synthesized for activity testing in biochemical assays. Following this confirmation, the activity of the compounds was assessed by cellular toxicity assays as described in the ‘Methods’ section. After compound synthesis and *in vitro* testing, seven targets gave at least one chemical series with confirmed biochemical activity measured with a minimum of two replicates, see Supplementary Table 1. This represents an attrition rate of roughly 50%, which is consistent with our previous ELT screening experience at GSK. Antibacterial activity was demonstrated against *S. aureus* for several chemical series identified from five targets, methionyl-tRNA synthetase (MRS), isoleucyl-tRNA synthetase (IRS), methionine aminopeptidase (MetAP), undecaprenyl pyrophosphate synthase (UppS) and acetyl-CoA carboxylase (ACC). The *S. aureus* UppS ELT hit series, its medicinal chemistry efforts, X-ray co-crystal structure and genetic and biochemical confirmation of antibacterial MoA were recently reported in a separate publication^47^. Similarly, the acetyl-CoA carboxylase hit series and characterization studies will be published at a later time. See Supplementary Methods for more details on compound characterization.

In Table 2, we disclose a representative novel MRS inhibitor belonging to a phenylbenzimidazole chemical series. It has potent enzyme activity in an MRS biochemical assay with an IC_50_ of 830 pM and possesses moderate antibacterial activity with a minimum inhibitory concentration (MIC) value of 0.5 μg ml^−1^ against *S. aureus*. Importantly, we conducted studies that suggest the cellular activity of the compound was linked to the inhibition of MRS. Increasing the levels of MRS in an overexpressing strain resulted in a higher MIC. We observed that benzimidazole 1 gave a significant MIC increase of 8-fold (uninduced) and >128-fold (induced with 0.1 μg ml^−1^ anhydrotetracycline) as compared to a vector control strain. These studies were conducted with a minimum of two replicates. In contrast, we were unable to observe the on-target antibacterial activity of the IRS inhibitor represented by the *t*-butylphenyl-piperazine amide 2 shown in Table 2. We include this series as an example of a hit that binds and inhibits its selected target but whose antibacterial activity could not be established as on-target. An exemplar from a MetAP benzimidazole series is shown (benzimidazole-triazole 3) with an IC_50_ of 0.35 μM in the biochemical assay (described in Supplementary Methods) but lacks antibacterial activity, hence its antibacterial MoA could not be confirmed in overexpression studies.

### *A. baumannii* panel

On the basis of our experience with the *S. aureus* campaign, we decided to expand the number of targets examined from the *A. baumannii* genome as a representative organism for the discovery of novel Gram-negative agents. To further simplify the process, we tested the antibacterial activity of all synthesized compounds against a panel of bacterial strains as shown in Supplementary Table 2. This eliminated the need to develop biochemical assays for each target and focused the effort on agents with cell penetration. Eighty proteins were selected for screening from the *A. baumannii* genome and, similar to the *S. aureus* panel described above, constructs were generated incorporating dual affinity tags (streptavidin-binding peptide and FLAG tag). *E. coli* was again used as a heterologous expression host and cell pellets, confirmed to be expressing protein, were thawed on the day of selection and lysed (described in Supplementary Methods). Each target protein was purified using streptavidin agarose resin, eluted with biotin and quantified using an Agilent Bioanalyzer all on the same day as the selection experiment. This removed the need for large-scale protein preparations to be conducted and removed a freeze/thaw cycle that can often compromise the quality of the reagent. Of the 80 targets, 70 were successfully purified using this rapid process. Our panel screen gave positive ELT selection signal for 52 targets and 18 were prioritized for off-DNA synthesis. After off-DNA synthesis of 3–5 chemical series per target and/or similarity searching of the GSK compound collection, 17 of the targets had at least one chemical series with activity in the antibacterial MIC assay panel and 3 targets had compounds that show positive MoA data. We disclose three chemical series identified from these targets with confirmed on-target activity shown in Table 2. LpxA, involved in lipid A biosynthesis, yielded two chemotypes with multiple compounds showing on-target activity. We show a general structure of a chemical series for one of the LpxA chemotypes and a particular aryl-urea series 4 with confirmed on-target MoA. UppS from *A. baumannii* was again found to produce a high number of binders and three unique series with positive MoA were identified, one of these (compound 5) is shown in Table 2. LolA, a lipoprotein chaperone, afforded a chemical series with positive MoA with a disclosed tetrahydropyrido-pyrimidine exemplar 6 shown in Table 2. See the Supplementary Methods for additional details on compound characterization, assay methods and activity results across a panel of bacteria.

### *M. tuberculosis* panel

Having seen the ability of these panels to rapidly find ligands for a diverse set of protein targets, we proposed using the number of ELT binders to rank the tractability of proteins from *M. tuberculosis*. An additional goal of this screen was to obtain tool molecules that could help validate a target as pharmacologically relevant. We did not place any requirements for target inclusion other than that the protein had to be purified and contain either a 6-histidine or biotin affinity tag. We obtained a diverse group of targets from academic collaborators for ELT screening. Eleven academic partners provided 42 protein preparations for screening (Supplementary Table 1). Although little to no previous information existed on the chemical tractability for most of these 42 proteins, a small number of known, chemically tractable targets were included as ‘controls’ (for example, InhA and DHFR). The ranking of target tractability is shown in Fig. 2b. Twenty-seven out of 42 targets afforded enriched chemotypes, and 13 targets with a greater number of binders were selected for further data analysis. Each of these targets produced multiple chemical series, and we chose three–five chemotypes for synthesis and off-DNA evaluation. The synthesized compounds 7–9 (Table 2) were easily assayed in a biochemical assay from the reported literature and we disclose three distinct chemical series with potency varying from 492 nM to 4.9 μM against dfrA (also known as DHFR). Compounds 7–9 were active against *M. tuberculosis* and the MIC values, measured in duplicate, are reported in Table 2. Although inhibitors of DHFR are already widely known, the mixture of familiar (compound 8 has a similar substructure to the known DHFR inhibitor methotrexate) and novel inhibitor chemotypes (7 and 9) identified by the ELT selection serves to validate the platform and demonstrate its powerful potential. InhA, the enoyl reductase targeted by the frontline therapeutic isoniazid, was screened but did not result in hits (see ‘Discussion’ section). Assessment of the compounds synthesized for other targets remains ongoing, with chemical series against four targets, KasA, LpdC, DHFR and DXR, confirmed in biochemical assays to date.

## Discussion

Tools to prioritize the abundance of uncharacterized proteins that will serve as the next generation of drug targets are crucial to the successful development of new medicines. The ELT target panel platform described here provides an opportunity to select protein targets based on an experimental and data-driven ligandability assessment. Starting with a diverse set of proteins that are of interest to a particular therapy area, the strategy rapidly identifies tractable targets and simultaneously defines novel starting points for therapeutic programme teams to explore. To our knowledge, no other report provides an experimental approach to tractability assessment that can evaluate a large set of proteins *de novo* in such a short period of time^15^,^17^.

Initially, the concept of screening many targets in parallel was proposed as a way to simply identify novel chemical matter for antibacterial drug discovery with less investment of both time and effort. The dual tagging, automation and miniaturization strategies described in the ‘Methods’ section (and previously) enabled the purification and screening of multiple targets in a single experiment^41^. We screened 39 proteins from *S. aureus* in the first panel, utilizing 36 libraries combined in a single pool, highlighting 14 tractable targets and, with limited chemistry investment, we discovered active chemical series for seven of the targets. Building on the success of the *S. aureus* panel, we initiated a refined strategy against a panel of protein targets from the Gram-negative pathogen *A. baumannii*. While most of these targets are essential for growth to the bacteria, some targets were added in this iteration, like LpxA, that are non-essential for *A. baumannii*. These are of interest to Gram-negative discovery projects in general as they are essential in most other Gram-negative pathogens and show severe growth impairment in *A. baumannii* when knocked out^48^. To rapidly identify potential lead compounds as well as explore a wider set of targets, we directly measured MIC, rather than developing individual biochemical assays for each target. Thus, the ability of hits to overcome bacterial permeability was added as a filter. This refinement increased the number of targets available for screening since no biochemical assay development was needed. However, the lack of biochemical data reduced our ability to confirm the activity of every chemotype as a tool rather than a cell penetrant lead. We observed active compounds by MIC testing, with hits from 17 of 18 targets showing activity against *A. baumannii*. Due to resource constraints and the focus of the therapeutic programme, only 3 of the 17 hits were fully characterized in mechanistic studies and are reported here.

By applying ELT screening to the antibacterial panels, we were able to rapidly interrogate 102 targets and discover active chemical matter against 24, a success rate of 24%. A similar rate of success for finding hits from high-throughput screening (HTS) against antibacterial targets has been reported earlier by GSK^49^. Indeed, the relatively low success in HTS along with the time required for lead molecule development as well as regulatory and commercial concerns are cited as reasons why many large pharmaceutical companies have abandoned this therapeutic area^50^. A comparison of 29 *S. aureus* targets common to both HTS and ELT approaches showed that in 21 of the 29 HTS campaigns, our ELT strategy would have predicted the outcomes (summarized in Table 3; for details, see Supplementary Table 3). In addition, for the remaining eight targets, the ELT screen successfully found hits for five targets where HTS did not. Another benefit of the ELT parallel screening method is that significantly less resources and time are needed to screen the collection of targets when compared to HTS. The time required to proceed from the set of purified proteins to an analysis of the chemical output averaged 3–4 months with an additional 2–3 months of time committed to chemical synthesis and activity testing. HTS assay development through hit analysis and hit qualification happens on a similar timescale (9–12 months). However, while only a single target is probed in HTS, here we describe a parallel ELT strategy that can interrogate many proteins in the same timeframe using only microgram amounts of reagent and far less human resource.

We hypothesized that applying chemistry resource to only the targets with the greatest number of binders would further improve the screening efficiency. The relationship between the ELT output and success in discovering active chemical series is illustrated by Fig. 2a. Here we have binned targets by outcome and ordered by the number of binders, finding that the successful targets were those with a greater number of binders. In the *S. aureus* panel, 7 of 14 highly ranked targets were confirmed as active and in the *A. baumannii* panel, three prioritized targets yielded compounds with confirmed MoA. The finding that ELT could rapidly winnow down a diverse set of targets and highlight those that were chemically ligandable provided an opportunity to improve our decision making and prioritize resources onto the most tractable targets. Utilizing the output of the ELT selections to predict downstream target success, both time and money can be saved while simultaneously identifying novel chemical starting points. We therefore hypothesize that ELT panels can additionally be used for pure tractability assessments for any group of interesting targets.

This was explored in a third panel by screening against *M. tuberculosis* proteins. As focus shifted from the identification of new chemical matter to tractability assessment, we chose to screen only against high-quality purified protein. The benefit of this can be seen in the reduced attrition of targets entering selection, with 41 of 42 proteins being amenable to ELT at the selection stage (Table 1 and Supplementary Table 1). Targets were ranked by decreasing number of binders (Fig. 2b). Active chemical matter for three high-priority targets (DHFR, KasA and LpdC) and one medium-priority target (DXR) has been confirmed, and chemotypes for the nine remaining targets are currently being evaluated. The effects of refinements and advances in the ELT process and data analysis are evident when comparing the panels shown in Fig. 2, which illustrates increases in both the number of binders found and number of targets with signal. This increased information density enabled the ranking of targets by tractability, allowed for improved discrimination between targets and facilitated better decision making.

**Figure 3:**
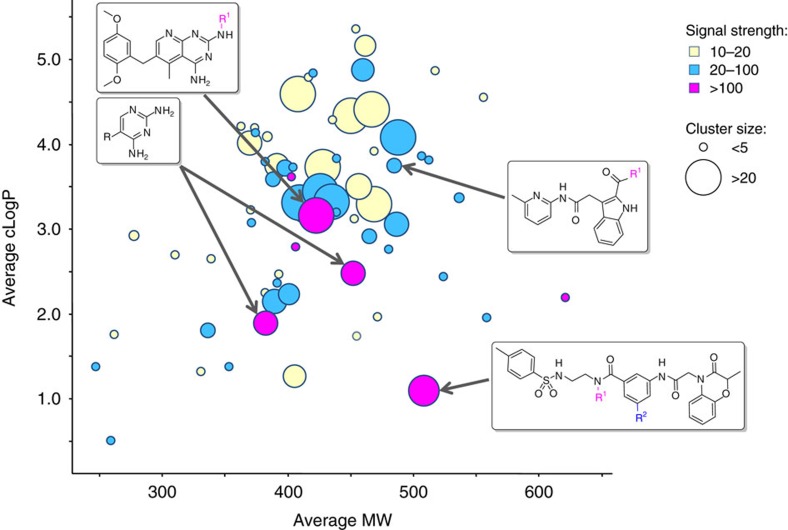
Representative data set of enriched binders for the *M. tuberculosis* target DHFR. The average molecular weight of clustered chemotypes is plotted versus average cLogP. Clusters are sized by the number of members and coloured by maximum signal strength. Chemical series with active molecules are indicated by their scaffolds.

The challenges in interpreting data and planning chemical synthesis have been met by advances in analysis tools and streamlining the planning of chemical synthesis. Historically, we examined an individual library through cube analysis to provide guidance for off-DNA hit to lead efforts^30^,^31^,^32^,^33^,^35^,^37^,^51^. Figure 3 shows a representative data set for a single target, DHFR, where chemical clusters are plotted with their average molecular weight versus the average cLogP. This is a common representation of the data that allows for rapid comparison of chemical series by signal intensity and also by physicochemical properties. Chemical series with higher signal intensity and larger cluster size that also fell into more attractive physicochemical space were selected for follow-up^46^. All targets were evaluated in parallel and common chemical reactions were executed in a concerted manner to increase efficiency. Representatives from each of the highlighted chemical series were then synthesized off-DNA and tested in biochemical or cellular assays. Additional visualizations were generated to examine potential structure activity relationships^37^,^51^. The structure activity relationships analysed within each chemical cluster allows for rapid pharmacophore identification and expansion of a single hit into a chemical series.

A key component of our strategy is that attrition is expected and that a negative result is not predictive, it simply helps focus on success at each step. It should be emphasized that we aim to expand the possibility of discovery to a wider range of targets and then redirect focus onto ligandable targets with speed and efficiency and do not advocate ignoring difficult targets. It is also important to note that targets with fewer ELT binders under our screening conditions may in fact be highly tractable under different conditions. InhA, the target of tuberculosis drug isoniazid, was found to have low tractability despite having known ligands. This enzyme is known to require cofactor to bind inhibitors and has an active site loop that plays a critical role in inhibition^52^,^53^,^54^. Recent publications report ELT selections for InhA consistent with the notion that the presence of cofactor improves this target’s tractability^33^,^42^. In fact, the method described here could be used to test the same target under many conditions or multiple constructs of the same target for optimization. These conditions may influence the stability of the protein or the conformation of the active site, which will impact the outcome of the ELT selection. Previous reports have focused on the discovery of ligands specific for protein conformations or complexes, either by screening in the presence of cofactors or against homologous proteins and our technique can be easily adapted for similar screens against any group of proteins to produce a relevant ranking^33^,^42^,^55^.

The ELT screening campaigns presented here have successfully identified multiple antibacterial compounds with confirmed MoA and have led to multiple lead optimization programmes. We have also applied this principle to other novel target areas, such as deubiquitinases, methyl readers, bromodomains and histone methyltransferases, and current studies are underway to include additional target areas (for example, the kinome), expanding the potential impact of this strategy. In cases where modulation of many targets converges on a single disease or phenotype, we are designing ELT panels where all of the off-DNA synthesis converges on a single phenotypic assay. The result will be a tractability ranking for all of the targets connected to a particular disease state along with potential tool and lead molecules to further explore the biology. Given the highly competitive, challenging and expensive endeavour of drug discovery, it is crucial to focus resource where it is most likely to have a positive impact. Our strategy enables teams to quickly identify targets to focus on and we see tremendous potential for this approach to impact other areas throughout the R&D community.

## Methods

### Just-in-time protein purification

*E. coli* cell culture expressing target protein was stored as a frozen pellet until the day of selections. Approximately 200 mg of cell pellet was dissolved in B-PER cell lysis solution (Thermo 90084) and incubated at room temperature for 20 min with occasional stirring. Solutions were divided evenly into eppendorf tubes and centrifuged at 18,000*g*, 4 °C for 20 min. A 10 ml filter spin column (Pierce 89898) was prepared for each sample. The column was loaded with 400 μl volume of 50% slurry of Streptavidin Ultra-link Resin (Peirce 53114). Solution was allowed to flow through and 5 ml of ELT buffer (50 mM Tris-HCl pH 7.5, 0.15 M NaCl, 0.1% Tween 20) was added to equilibrate resin bed. The resin was centrifuged for 3 min at 500 r.p.m., the column capped and 100 μl of buffer added to keep the resin moist. A measure of 3 ml of clarified cell lysate was loaded onto the prepared resin bed and incubated on ice for 15–20 min. The solution was allowed to flow through the column and centrifuged for 3 min at 500 r.p.m.. A measure of 10 ml of ELT buffer was loaded to wash the resin and centrifuged for 3 min at 500 r.p.m.. A measure of 500 μl of 2.5 mM D-biotin was added (Sigma B-4501). To make this stock solution solid, D-biotin was dissolved to 0.5 M in 1 M NaOH, diluted to 2.5 mM with ELT buffer and incubated on ice for 45 min with gentle and occasional stirring. Column was transferred to a clean tube (Falcon) and eluted by centrifugation at 500 r.p.m. for 3 min. Purified proteins were quantified using a Bioanalyzer (Agilent 230 Protein Chip Kit, Fisher NC9415734) where purity and approximate concentrations were accessed. A yield of 30–200 μg of target protein was observed with purity varying from ∼60 to >95%.

### Encoded library preparation for selection

ELT libraries were combined into a pooled library for screening purposes. For each screening effort, the then available libraries were ligated to contain a unique library identifier and then combined into an equimolar mixture at a concentration of 0.5 mM. After final library closing to add a pool identification sequence, these libraries could then be identified as associated with each individual condition after sequencing (see Supplementary Methods).

### PhyNexus tip preparation and automated selections

Each condition was screened using a Beckman Coulter, BioMek FX robot. Four 200 μl Phynexus tips packed with 5 μl beds of either M2 anti-FLAG, IMAC or streptavidin resin were employed per target and equilibrated before selections using ELT selection buffer (50 mM HEPES, pH 7.5, 300 mM NaCl, 1 mM CHAPS, 10 mM imidazole, 0.1 mg ml^−1^ sheared salmon sperm DNA, 1 mM MgCl_2_, 1 mM CaCl_2_, 1 mM DTT). Tips were used within 2 h of equilibration. Ten nmoles of pooled library were suspended in 100 μl of selection buffer and used as input for each selection. Ninety-six-well plates were prepared containing 10 μg of target protein suspended in 110 μl of selection buffer per well in row A for each of four rounds of selection. A measure of 110 μl selection buffer was pipetted per row into rows B–G to serve as washes. In the case of streptavidin tips only, 1 mM biotin was included in the wash buffer in row B to reduce enrichment of streptavidin binders. In addition, 120 μl of selection buffer in row H was used for heat elution. A BioMek FX protocol was developed in which purified protein from row A was captured for 15 min on Phynexus tips, washed once with selection buffer from row B, exposed to library pool for 30 min, washed five times with selection buffer (rows C–G) and heat eluted in selection buffer from row H at 90 °C to recover binders. Four rounds of selection were performed in this manner with the output of each round carried forward as the input to the successive round with fresh protein and new tips.

### Selection output processing

Outputs of each selection condition were purified using a Qiagen Qiacube running a nucleic acid purification protocol and then quantified by qPCR run on a Roche Lightcycler. Total DNA output ranged from 2 × 10^7^ to 1 × 10^9^ copies, and was PCR amplified with primers adaptors to add sequences compatible with Illumina sequencing flowcells. PCR output was purified using Agencourt AMPure XP SPRI beads according to the manufacturer’s instructions and then quantitated on an Agilent BioAnalyzer using a high sensitivity DNA kit. Final concentration of amplicon for each sample was between 2 and 40 nM. Portions of products were loaded to generate ∼20 million clusters per selection condition on an Illumina GAII or HiSeq platform.

### Sequencing and data analysis

Libraries were pooled in equal volume and each library had a unique DNA tag that enabled sequence deconvolution. Each warhead was uniquely tagged with a specific DNA tag combination (described previously^20^) and also included a degenerate region to account for sequencing or amplification artifacts. Compounds with identical degenerate regions were counted as a single occurrence.

For every library and for each target, the total number of unique warhead sequences was counted. Compounds were then grouped by the different possible combinations of building blocks (for example, single cycle ‘mono’ synthons, two cycle ‘di’ synthons and so on). The total count for every combination was then used to calculate signal as the fold value of number of copies divided by the expected noise level (as defined by the theoretical distribution of copies). Every chemotype with a signal value >2 was reported. All chemotypes with signal were compared against a database of historic target. Any chemotypes that also had signal against a historic target were flagged as promiscuous and removed from further consideration. Any chemotype with signal against empty matrix (no-target control) was similarly removed. Of the remaining chemotypes, only those that had signal against a single target from the panel were considered for final evaluation, except in cases where the target had highly similar binding pockets, substrates or cofactors.

A data table was created containing all chemotypes and their associated signal values for each target/library combination. These data were graphed in Spotfire and used to rank the targets by total number of chemotypes. To prioritize off-DNA follow-up synthesis, chemotypes with signal were clustered together based on calculated Tanimoto similarity by two-dimensional fingerprints. Clusters with high representation and favourable physical chemical properties (lower average molecular weight, lower average cLogP) were chosen for further evaluation and off-DNA chemistry planning.

### Data availability

All relevant data are available from the authors on request.

## Additional information

**How to cite this article:** Machutta, C. A. & Kollmann, C. S. *et al*. Prioritizing multiple therapeutic targets in parallel using automated DNA-encoded library screening. *Nat. Commun.* **8**, 16081 doi: 10.1038/ncomms16081 (2017).

**Publisher’s note:** Springer Nature remains neutral with regard to jurisdictional claims in published maps and institutional affiliations.

## Supplementary Material

Supplementary Information

## Figures and Tables

**Table 1:** ELT screening progression.

**Phase**	***S. aureus***	***A. baumannii***	***M. tuberculosis***
*Ligandability assessment*			
Initial number of targets considered	39	80	42
Targets amenable to ELT selection	32	70	41
Targets with ELT signal	14	52	27
			
*Binding confirmation*			
Targets with off-DNA synthesis	14	18	13
Targets with confirmed active chemical series (IC_50_ and/or MIC)	7	17	4*
Targets with confirmed MoA	2	3	ND

ND, not determined.

The table shows a summary of the progression of protein targets through each tractability panel. For additional detail and a full list of targets prosecuted, see Supplementary Table 1.

^*^Off-DNA activity assessment ongoing for remaining nine targets.

**Table 2:** Representative chemical series discovered in three tractability campaigns.

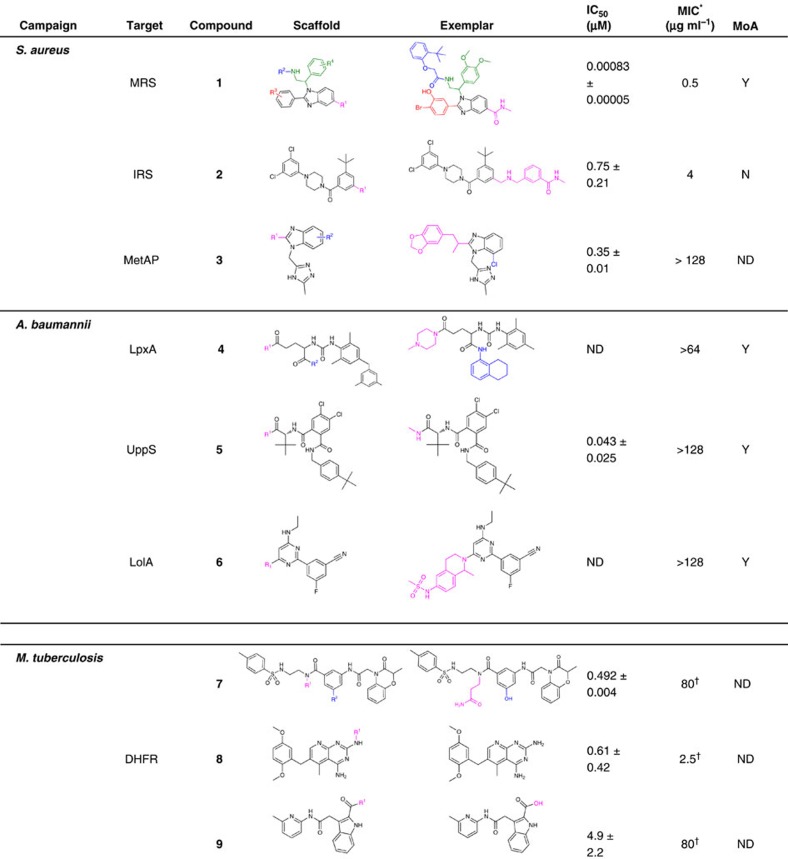							

ND, not determined; MoA, mode of action.

The scaffold column represents the selected pharmacophore with areas of substitution indicated by R. All data are reported for the single exemplar shown. IC_50_ values are reported as the average of two replicate experiments with s.d. values calculated using the n−1 method.

^*^Minimum inhibitory concentration measured against *S. aureus* WCUH29 wild type or *A. baumannii* BM652 efflux strains for *S. aureus* or *A. baumannii* campaigns with a minimum of two independent experiments.

^†^MIC unit is in μM and is determined against *M. tuberculosis* H37Rv.

**Table 3:** Consistency of HTS and ELT outcomes for 29 *S. aureus* targets.

	**ELT active**	**ELT inactive**
HTS active	4	3
HTS inactive	5	17

Comparison of target outcomes from ELT and HTS where the same target was screened by both methods. ELT and HTS outcomes agreed in 21 of 29 campaigns. For the remaining eight targets, ELT found hits for five where HTS did not.
