# Author Correction: Prioritizing multiple therapeutic targets in parallel using automated DNA-encoded library screening

**DOI:** 10.1038/ncomms16227

**Published:** 2018-07-13

**Authors:** Carl A. Machutta, Christopher S. Kollmann, Kenneth E. Lind, Xiaopeng Bai, Pan F. Chan, Jianzhong Huang, Lluis Ballell, Svetlana Belyanskaya, Gurdyal S. Besra, David Barros-Aguirre, Robert H. Bates, Paolo A. Centrella, Sandy S. Chang, Jing Chai, Anthony E. Choudhry, Aaron Coffin, Christopher P. Davie, Hongfeng Deng, Jianghe Deng, Yun Ding, Jason W. Dodson, David T. Fosbenner, Enoch N. Gao, Taylor L. Graham, Todd L. Graybill, Karen Ingraham, Walter P. Johnson, Bryan W. King, Christopher R. Kwiatkowski, Joël Lelièvre, Yue Li, Xiaorong Liu, Quinn Lu, Ruth Lehr, Alfonso Mendoza-Losana, John Martin, Lynn McCloskey, Patti McCormick, Heather P. O’Keefe, Thomas O’Keeffe, Christina Pao, Christopher B. Phelps, Hongwei Qi, Keith Rafferty, Genaro S. Scavello, Matt S. Steiginga, Flora S. Sundersingh, Sharon M. Sweitzer, Lawrence M. Szewczuk, Amy Taylor, May Fern Toh, Juan Wang, Minghui Wang, Devan J. Wilkins, Bing Xia, Gang Yao, Jean Zhang, Jingye Zhou, Christine P. Donahue, Jeffrey A. Messer, David Holmes, Christopher C. Arico-Muendel, Andrew J. Pope, Jeffrey W. Gross, Ghotas Evindar

Nature Communications 8: Article number: 16081; DOI: 10.1038/ncomms16081 (2017); Published: 07 17 2017, Updated: 07 13 2018

The original version of this Article omitted the following from the Acknowledgements:

‘We thank Robert Kirkpatrick for implementing the high throughput protein design strategy that enabled screening and triage of essential *A. baumannii* targets, based on whole genome sequencing and annotation of BM4454 strain; and Stephanie Van Horn, Allan Kwan, Elizabeth Valoret for *A. baumannii* genome sequencing and annotation.’

Also, the original version omitted an acknowledgement to Prof. Lydia Tabernero as one of our collaborators for supplying the purified proteins used in the Tuberculosis screen.

This has been corrected in both the PDF and HTML versions of the Article.

