# Supplementary material for: Prioritizing multiple therapeutic targets in parallel using automated DNA-encoded library screening
Source: Nat Commun. 2017 Jul 17;8:16081. doi: 10.1038/ncomms16081 (PMC5520047; doi:10.1038/ncomms16081)
Supplement: Supplementary Information [file ncomms16081-s1.pdf]

SI GUIDE

Title of file for HTML: Supplementary Information
Description: Supplementary Figures, Supplementary Tables, Supplementary Notes, Supplementary Methods and Supplementary References.

SUPPLEMENTARY INFORMATION

Supplementary Figures:

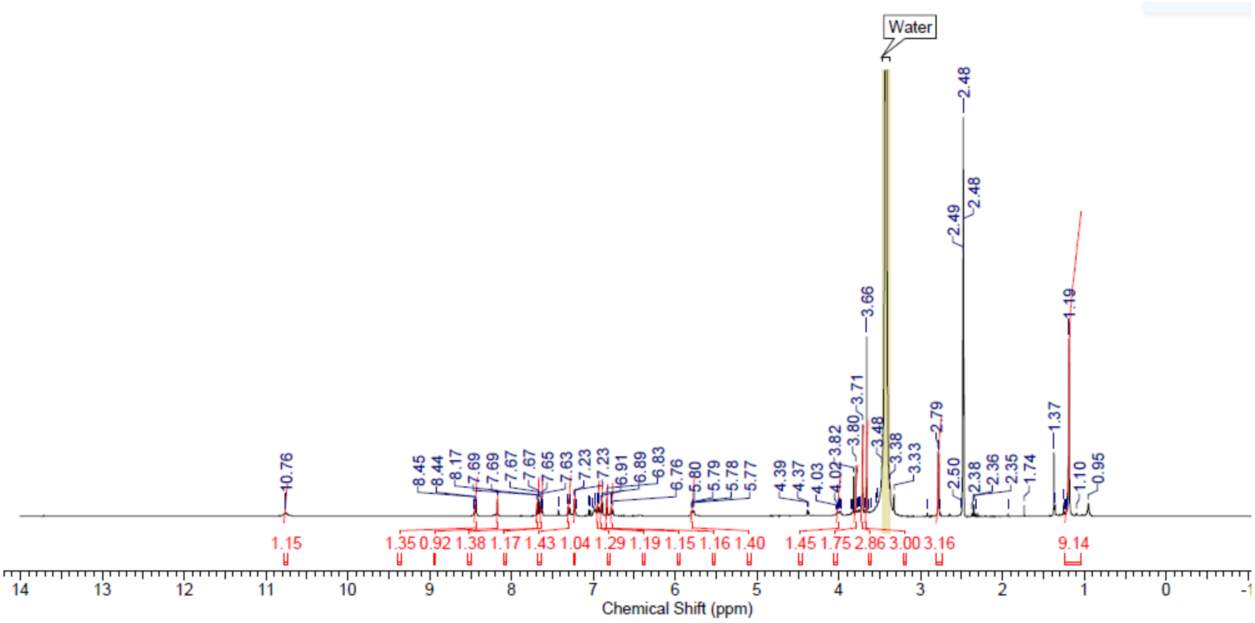

Supplementary Figure 1: ^{1}**H NMR (DMSO-d**$_{6}$**) spectrum of 1-1.**

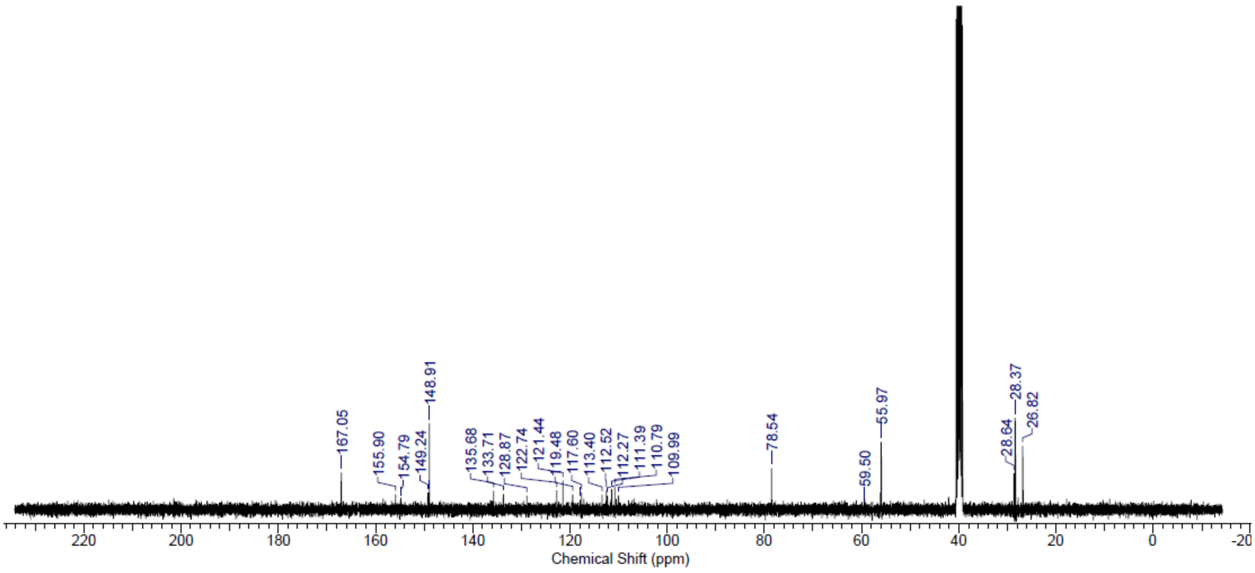

Supplementary Figure 2: ^{13}**C NMR (DMSO-d**$_{6}$**) spectrum of 1-1.**

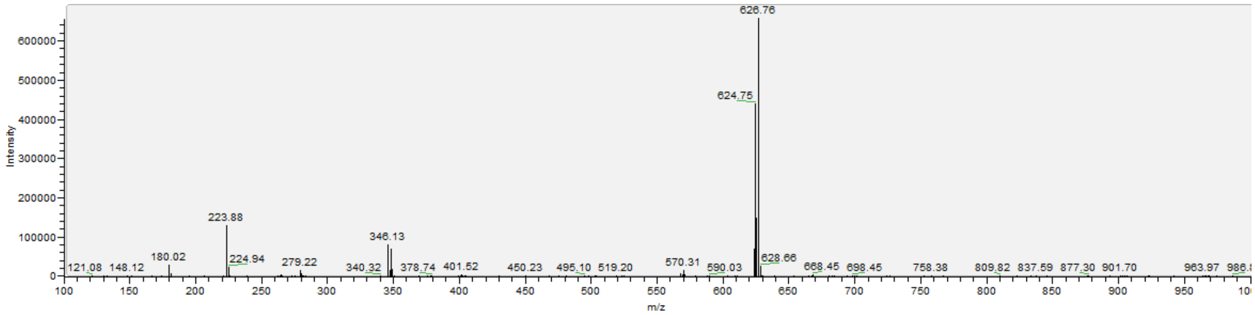

Supplementary Figure 3: ESI MS spectrum of 1-1.

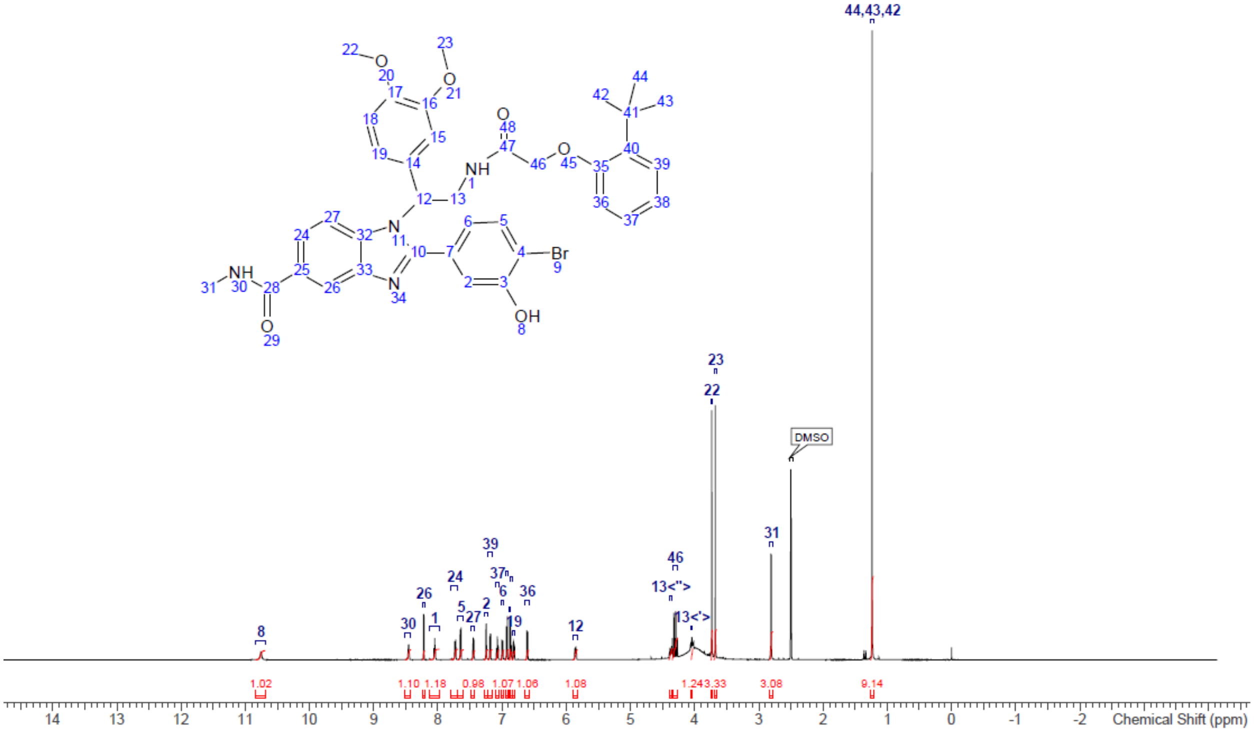

Supplementary Figure 4: ^{1}H NMR (DMSO-d_6) spectrum of 1.

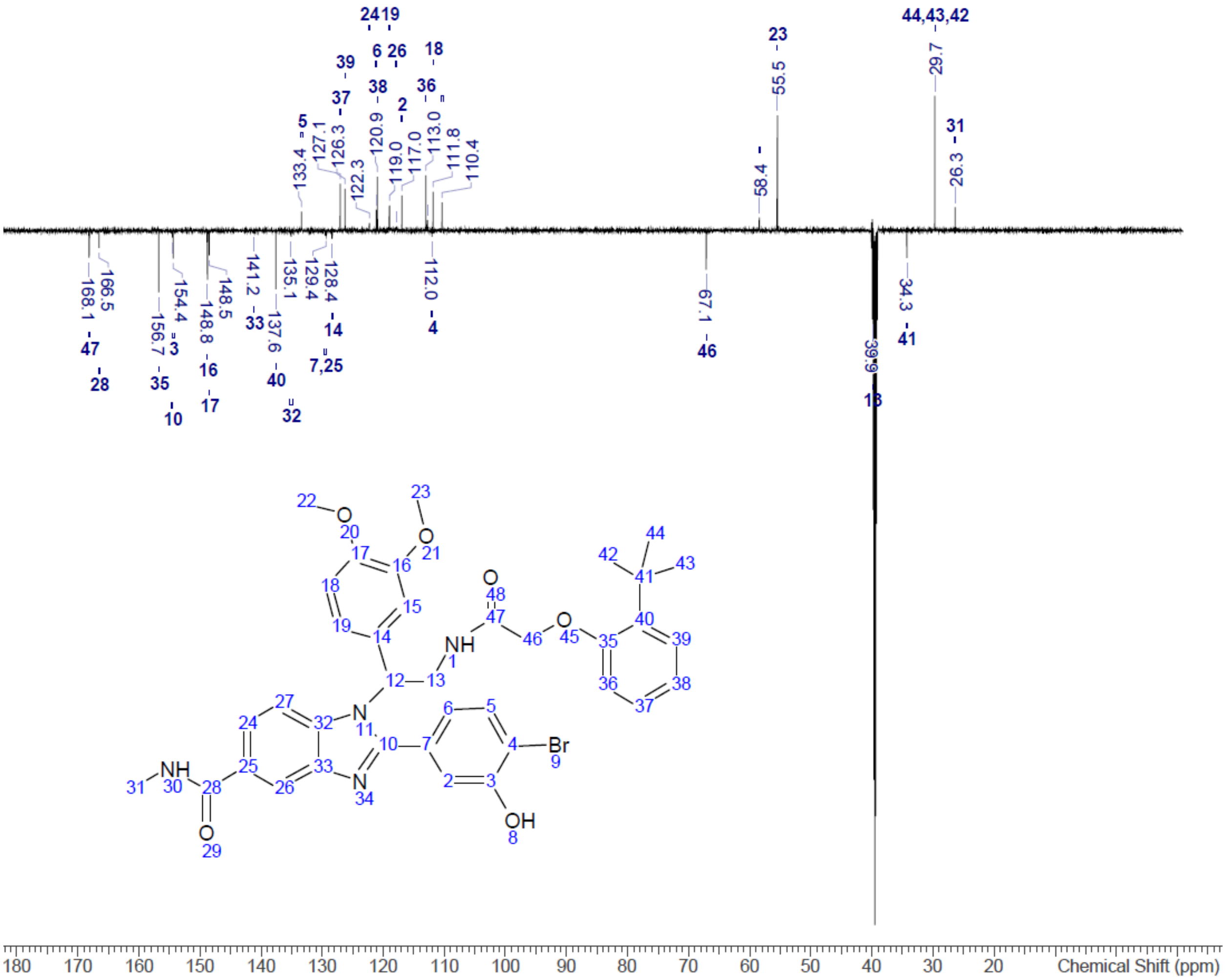

Supplementary Figure 5: ^{13}C NMR (DMSO-d_6) spectrum of 1.

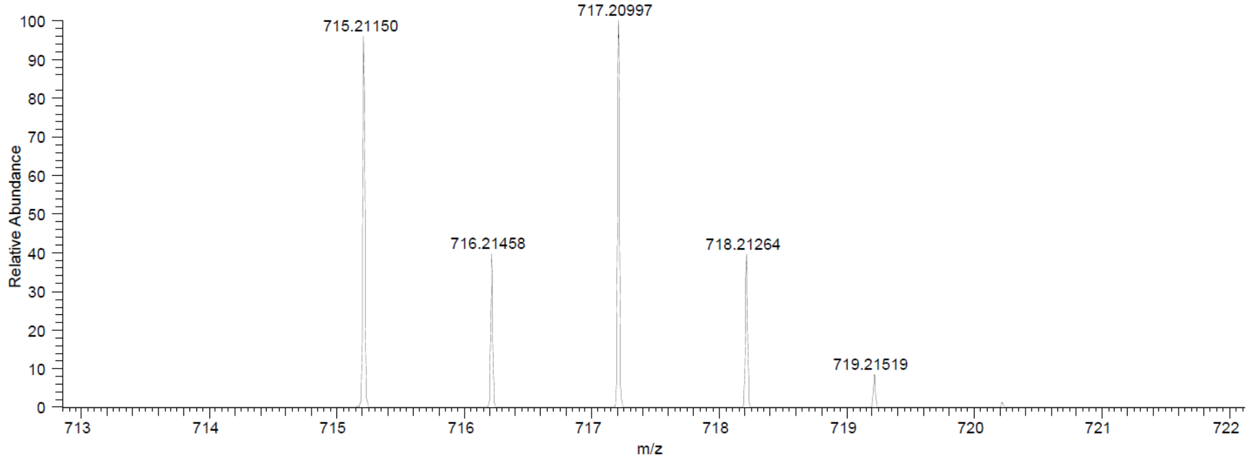

Supplementary Figure 6: HRMS spectrum of 1.

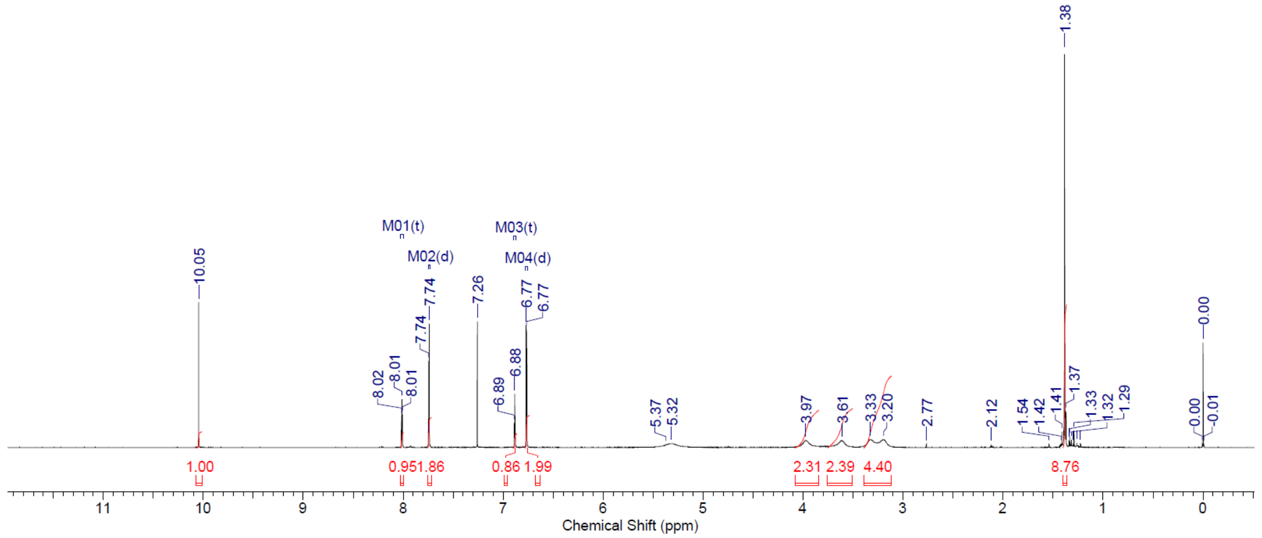

Supplementary Figure 7: ^{1}H NMR ($CDCl_3$) spectrum of 2-1.

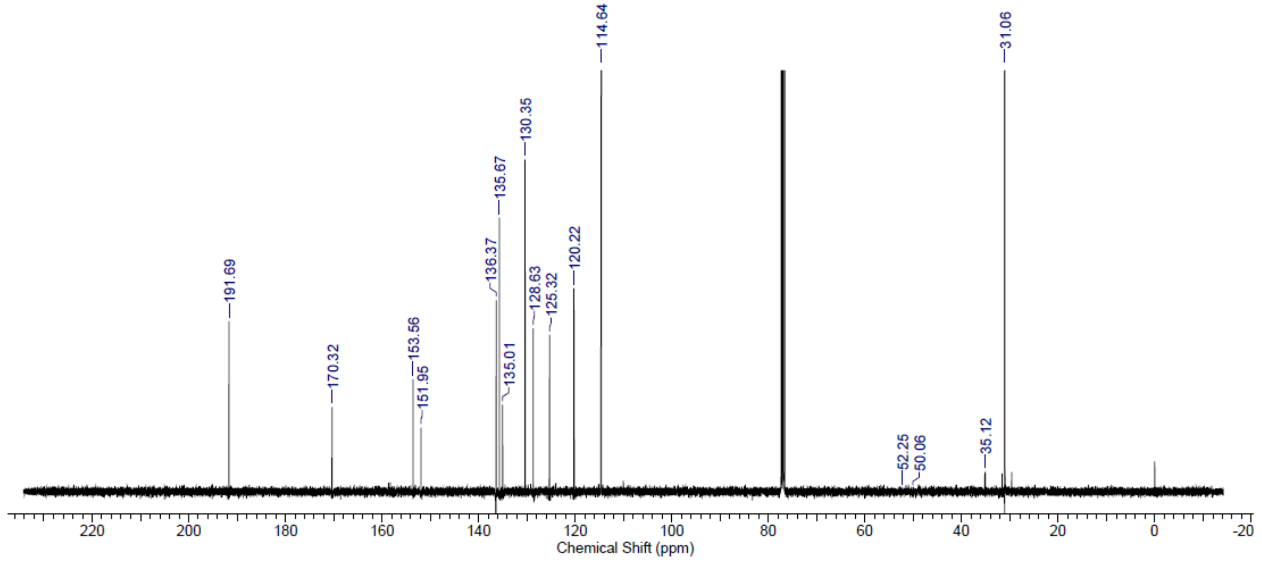

Supplementary Figure 8: ^{13}C NMR ($CDCl_3$) spectrum of 2-1.

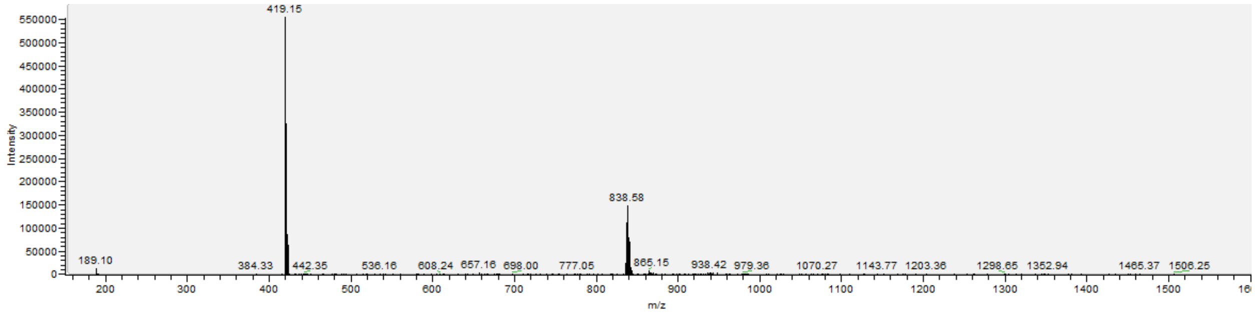

Supplementary Figure 9: ESI MS spectrum of 2-1.

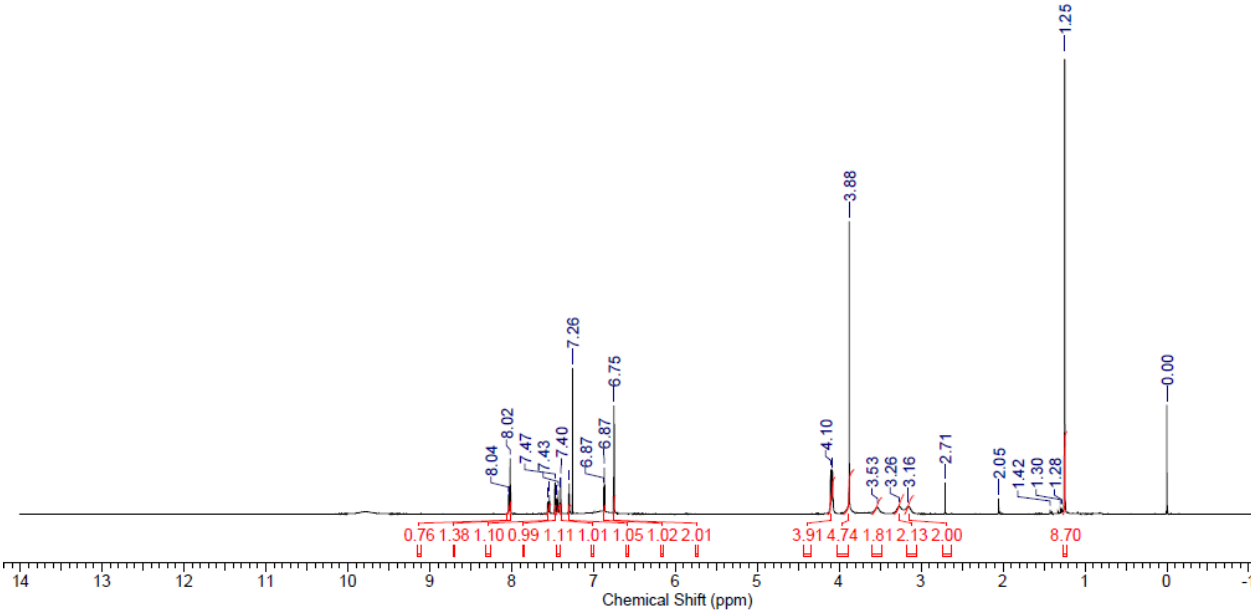

Supplementary Figure 10: ^{1}H NMR ($CDCl_3$) spectrum of 2-2.

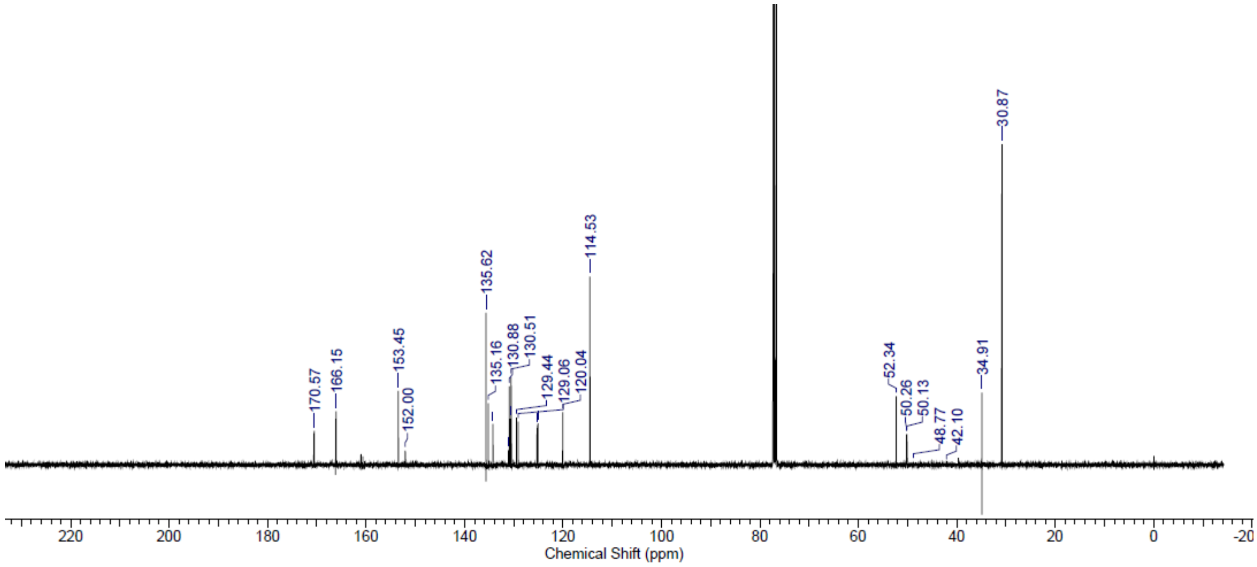

Supplementary Figure 11: ^{13}C NMR ($CDCl_3$) spectrum of 2-2.

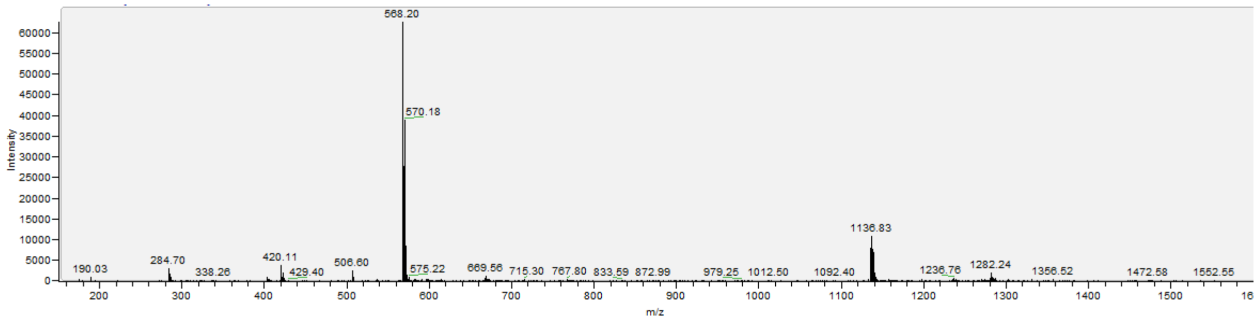

Supplementary Figure 12: ESI MS spectrum of 2-2.

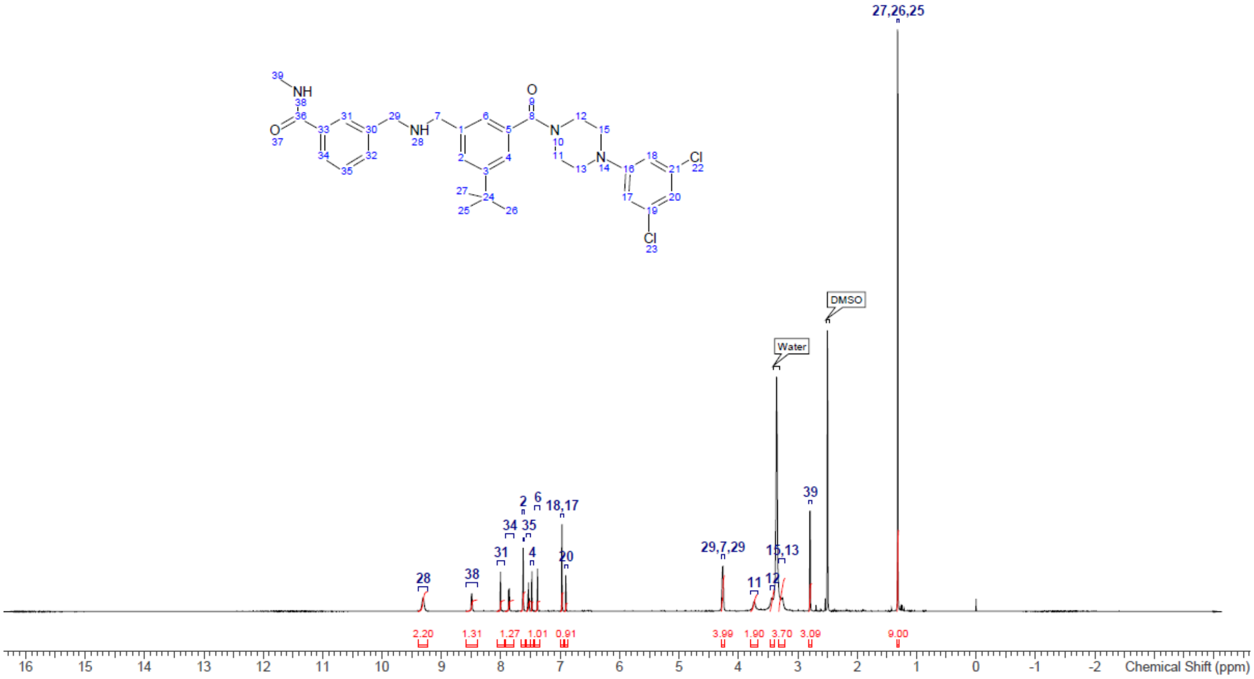

Supplementary Figure 13: ^{1}H NMR (DMSO-d_6) spectrum of 2.

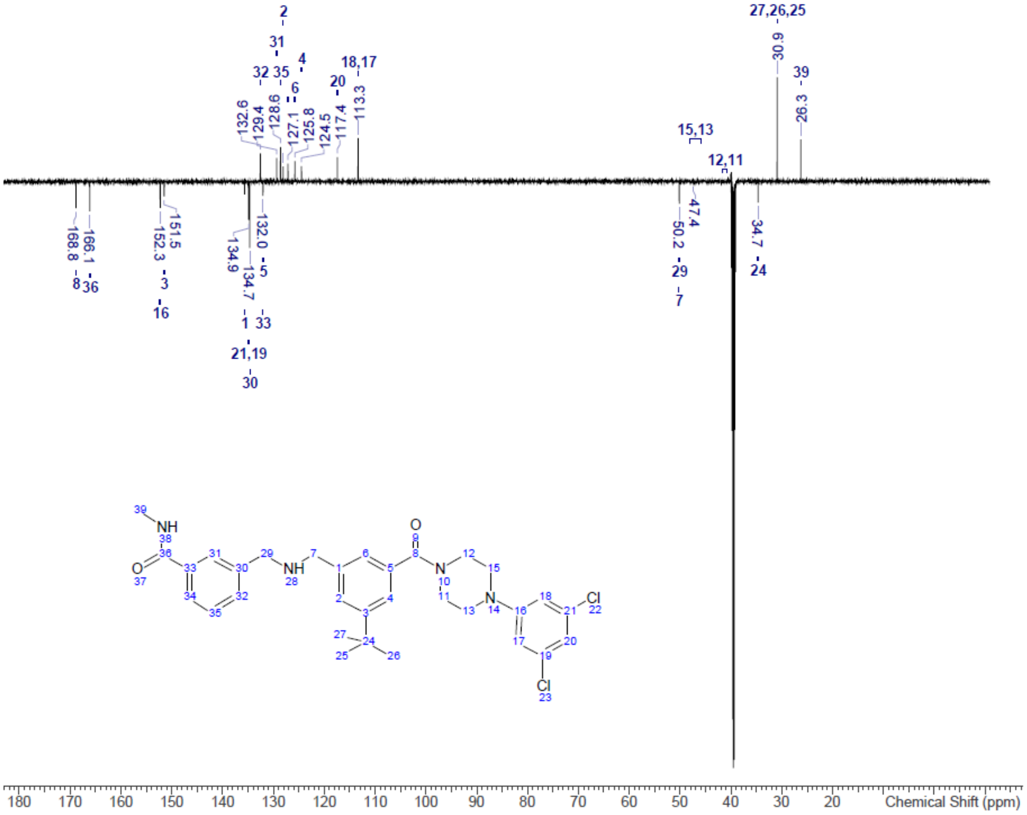

Supplementary Figure 14: ^{13}C NMR (DMSO-d_6) spectrum of 2.

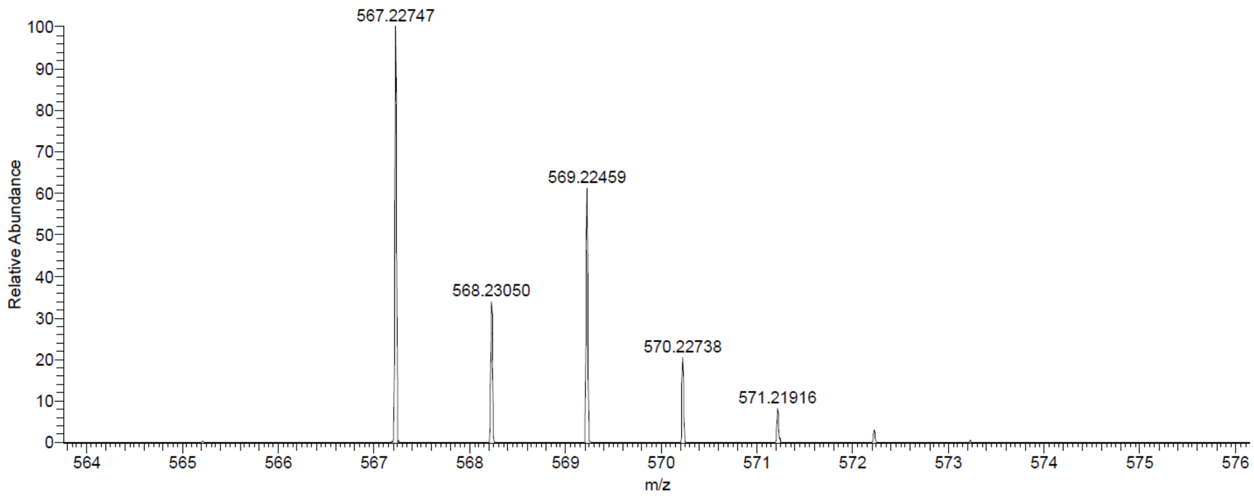

Supplementary Figure 15: HRMS spectrum of 2.

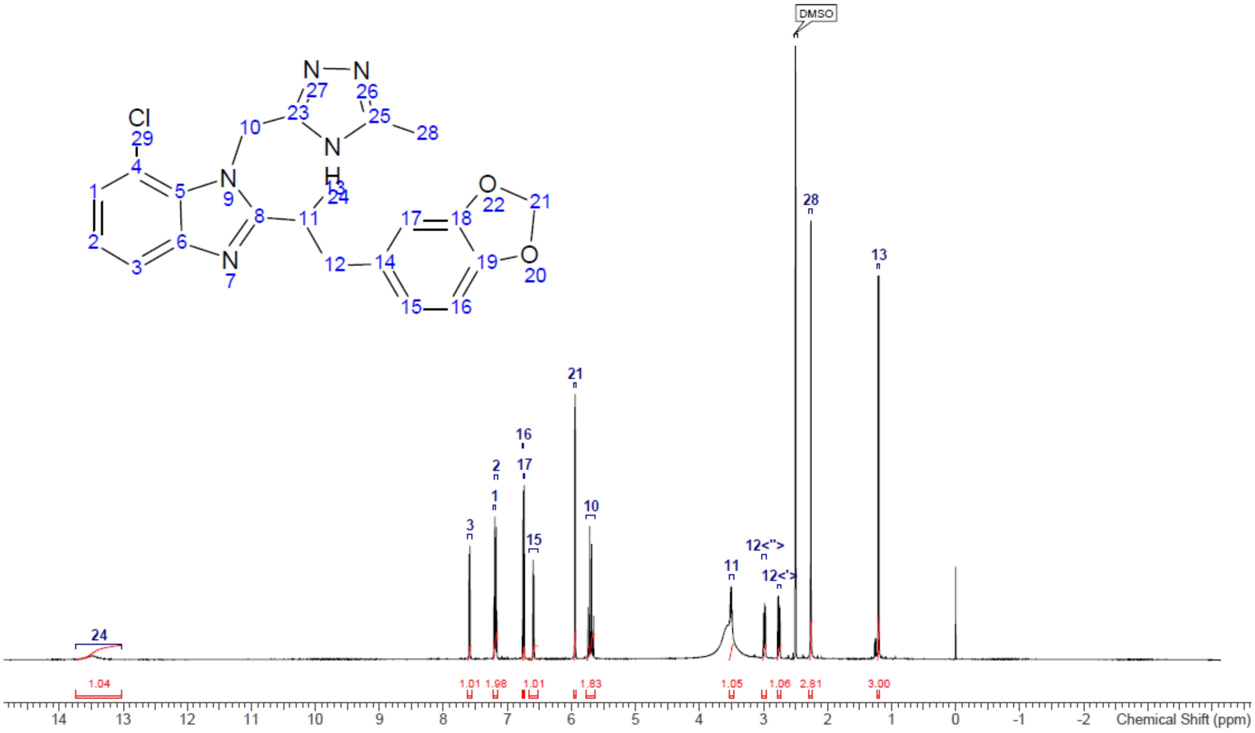

Supplementary Figure 16: ^{1}H NMR (DMSO-d_6) spectrum of 3.

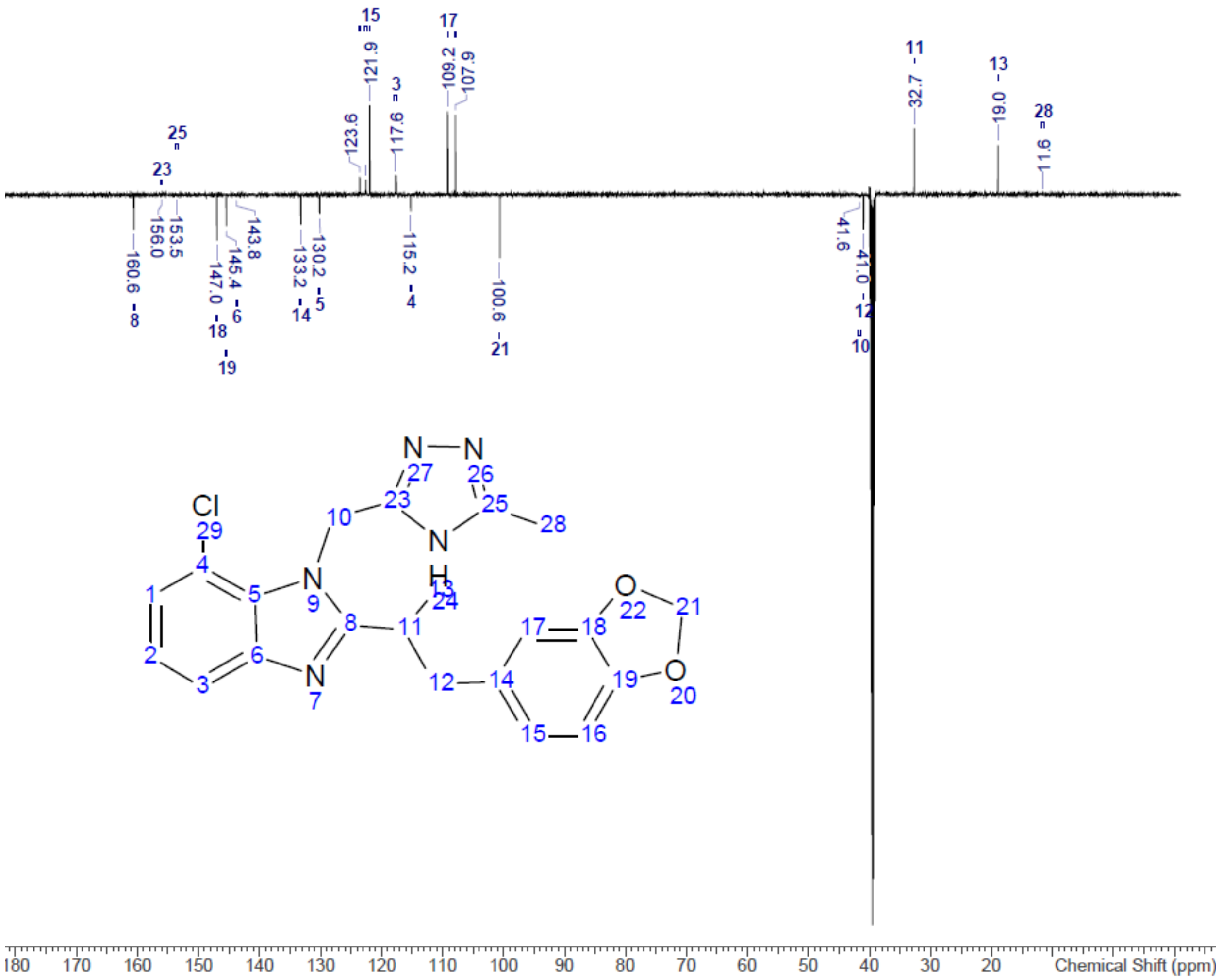

Supplementary Figure 17: ^{13}C NMR (DMSO-d_6) spectrum of 3.

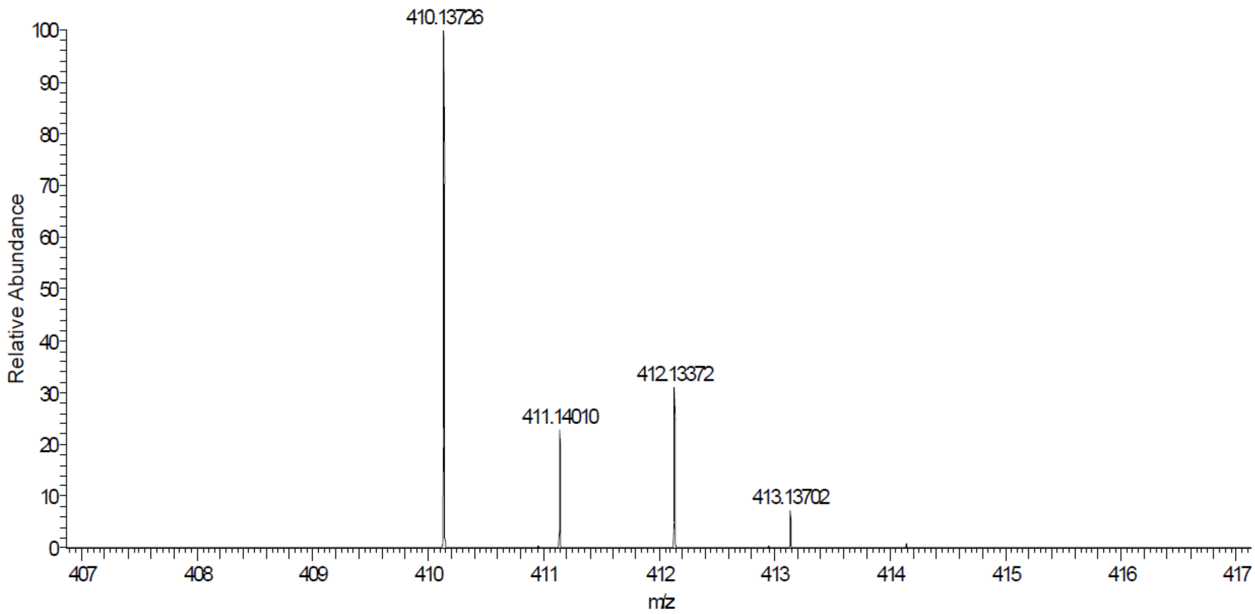

Supplementary Figure 18: HRMS spectrum of 3.

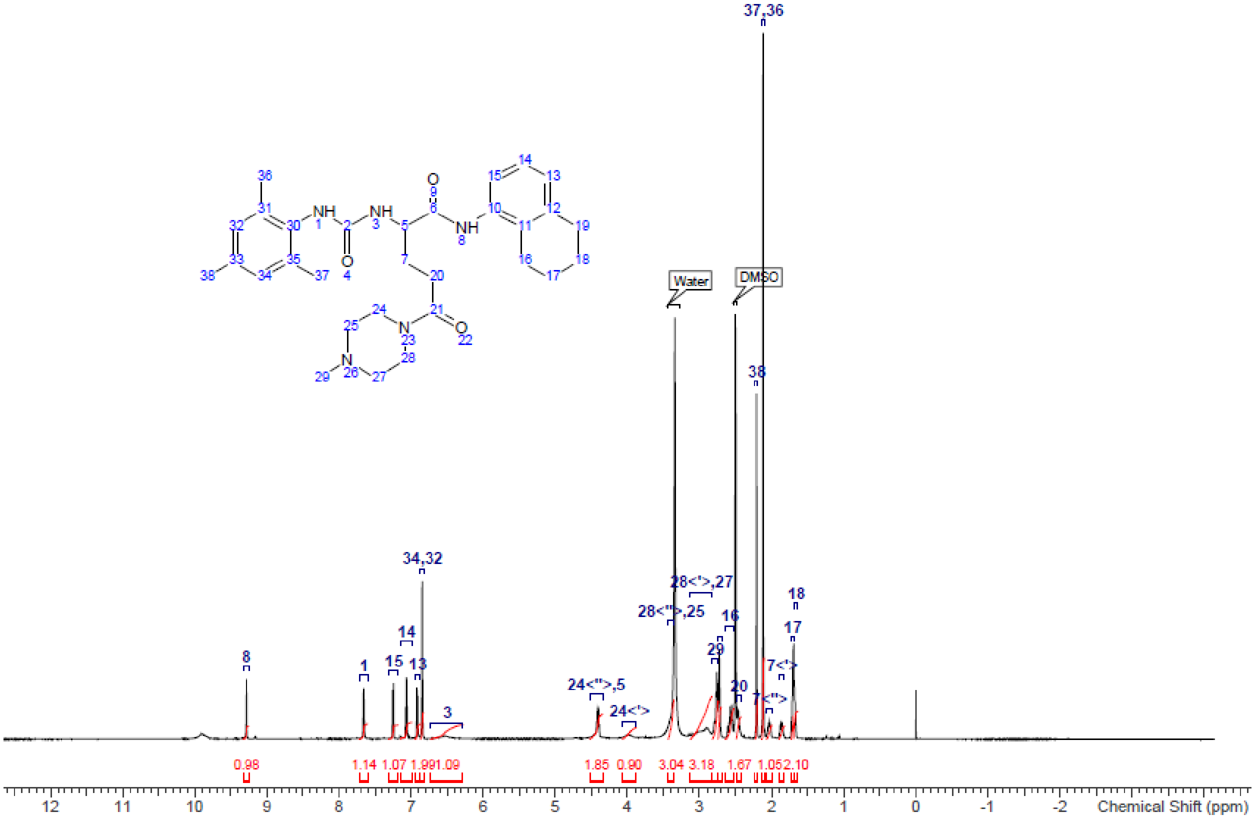

Supplementary Figure 19: ^{1}H NMR (DMSO-d_6) spectrum of 4.

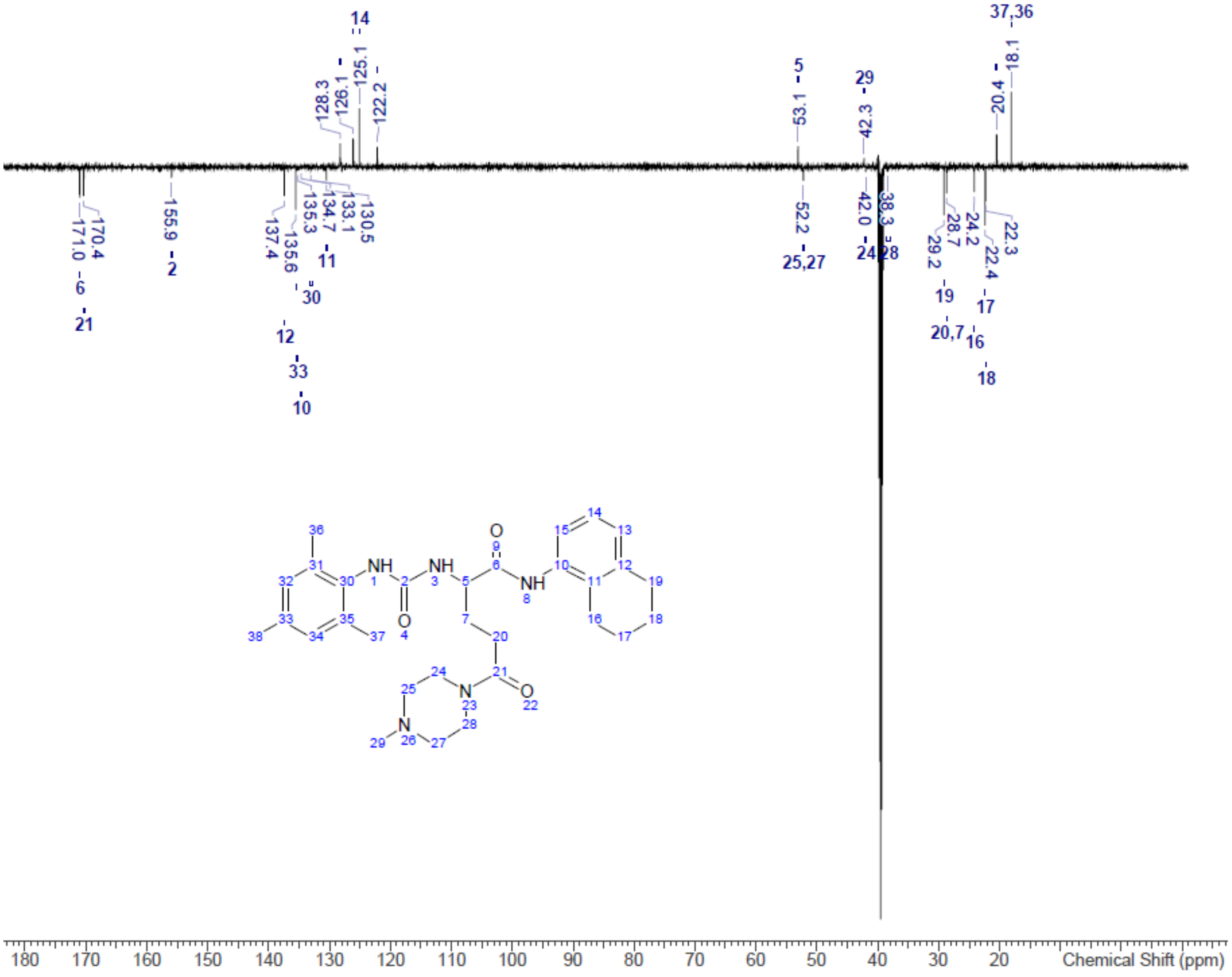

Supplementary Figure 20: ^{13}C NMR (DMSO-d_6) spectrum of 4.

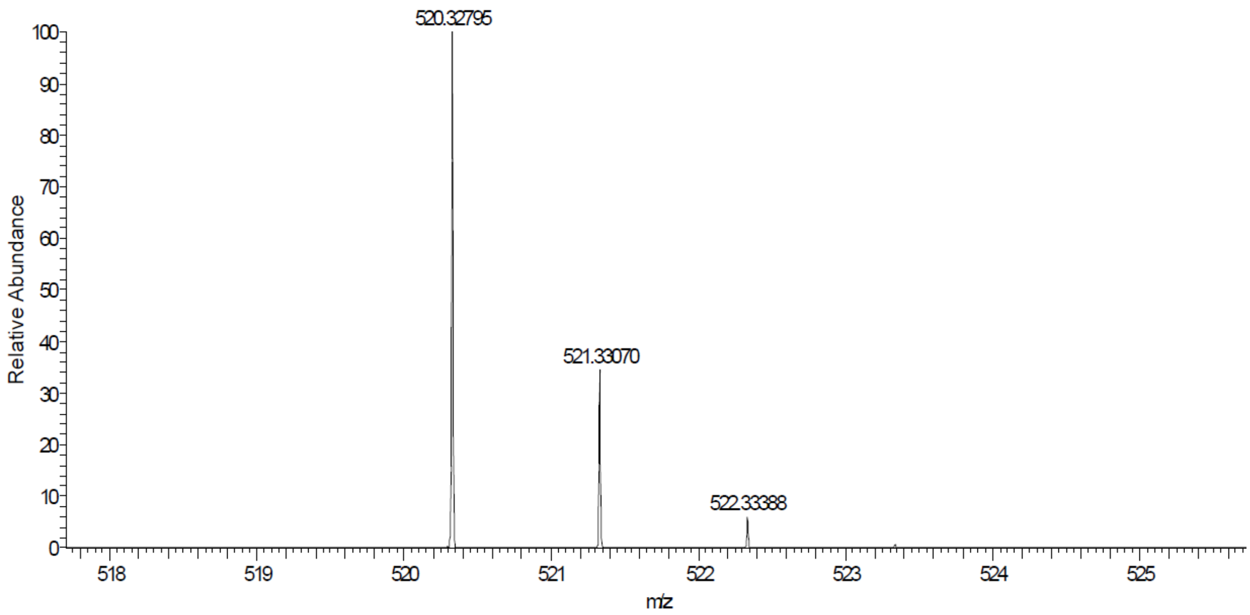

Supplementary Figure 21: HRMS spectrum of 4.

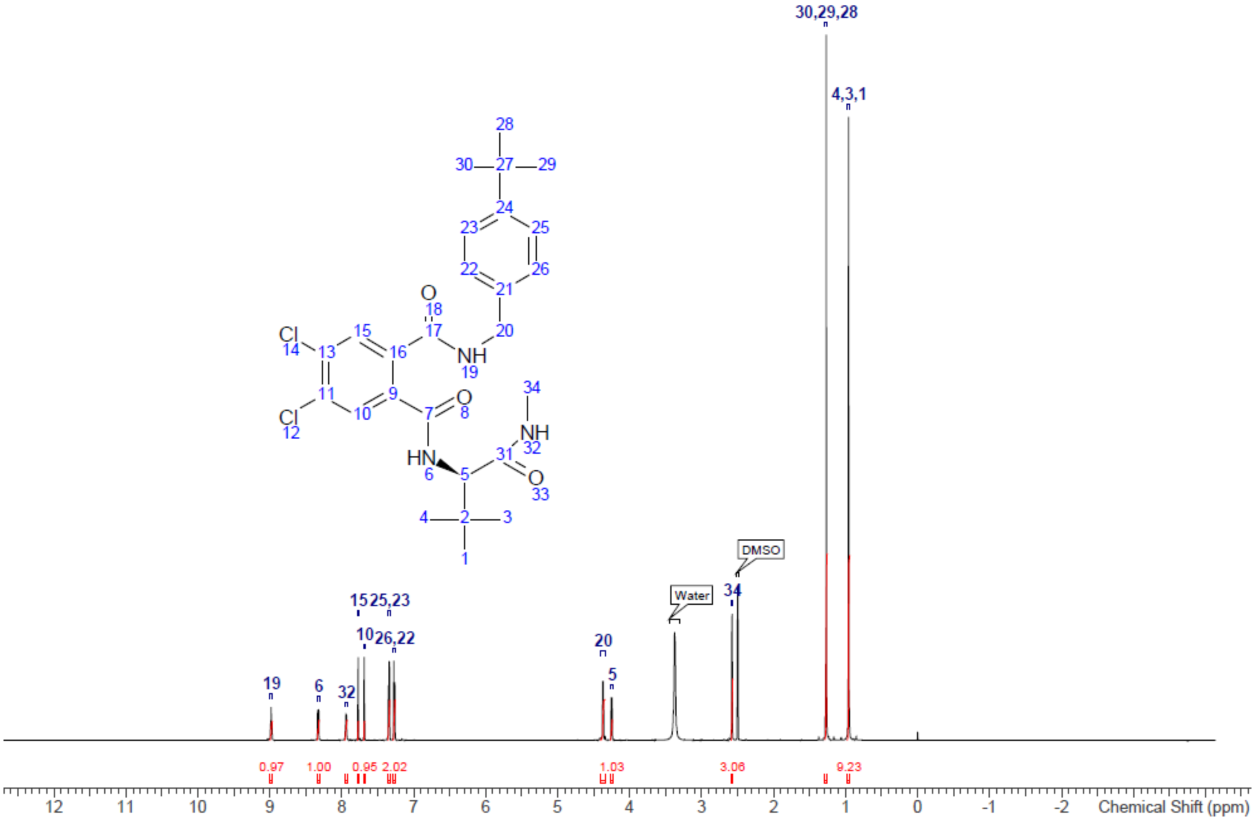

Supplementary Figure 22: ^{1}H NMR (DMSO-d_6) spectrum of 5.

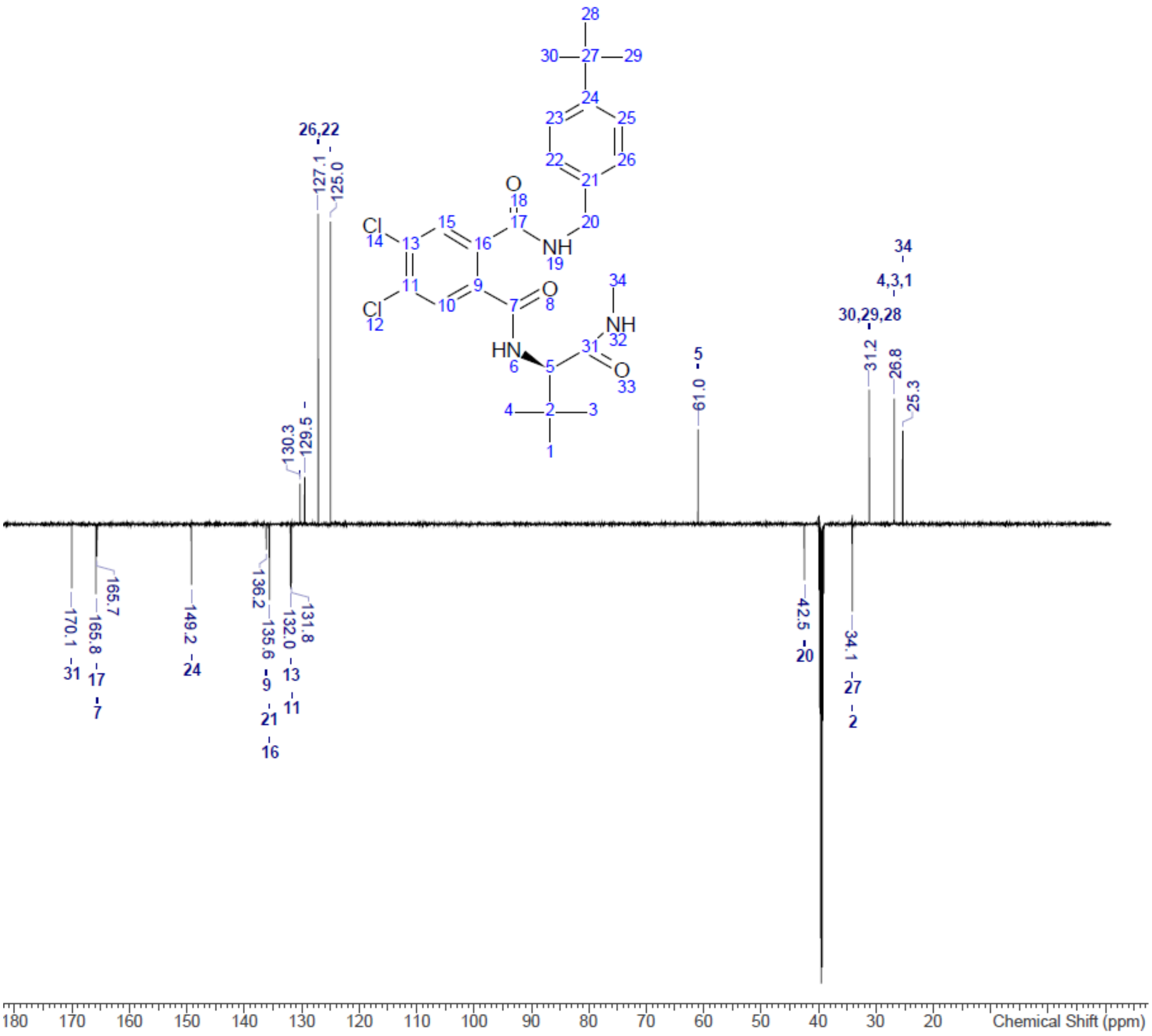

Supplementary Figure 23: ^{13}C NMR (DMSO-d_6) spectrum of 5.

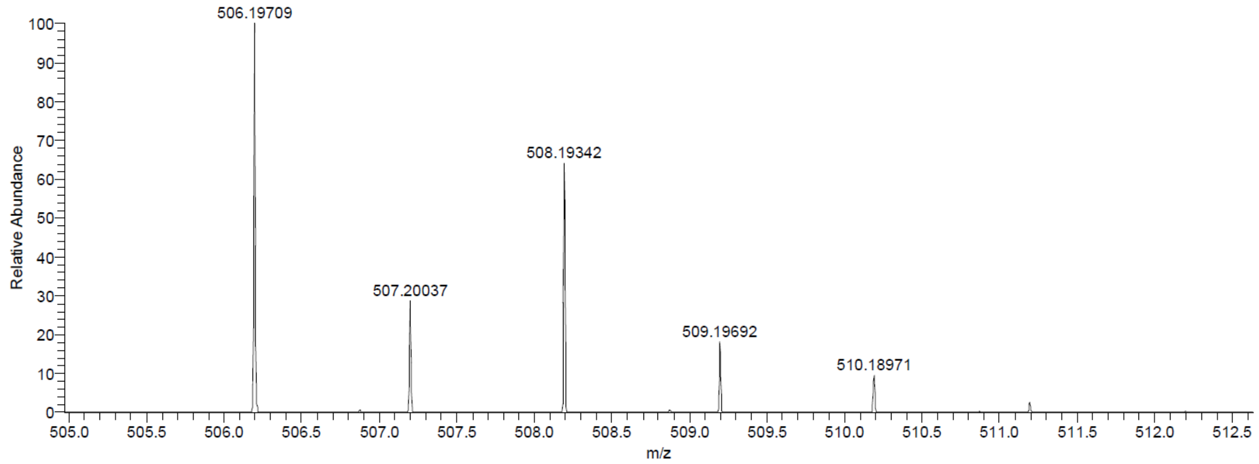

Supplementary Figure 24: HRMS spectrum of 5.

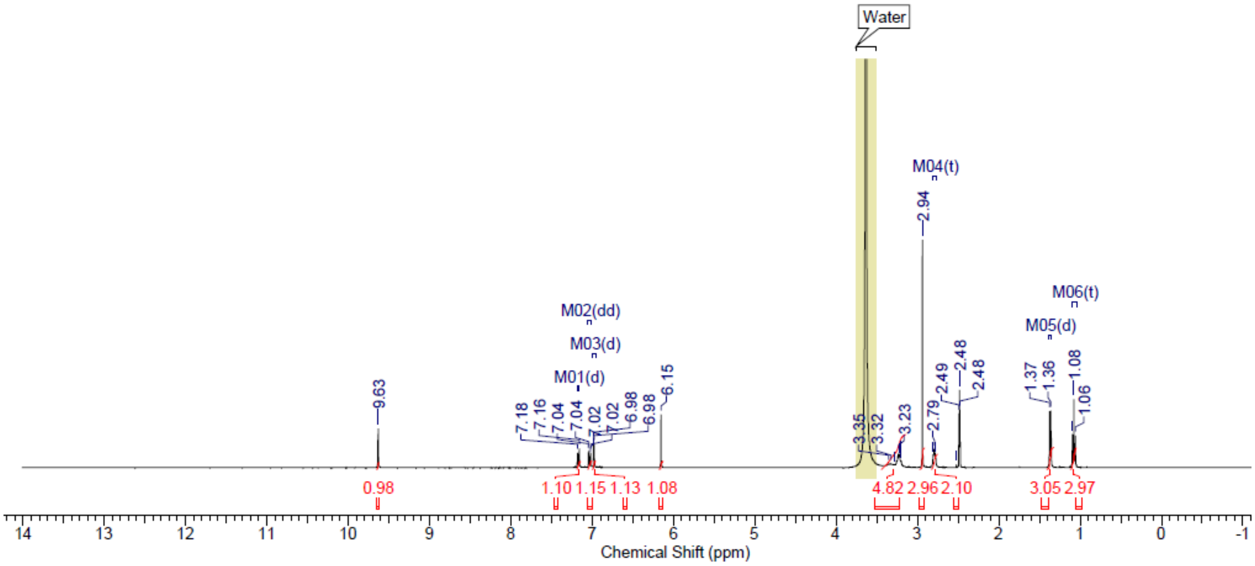

Supplementary Figure 25: ^{1}H NMR (DMSO-d_6) spectrum of 6-1.

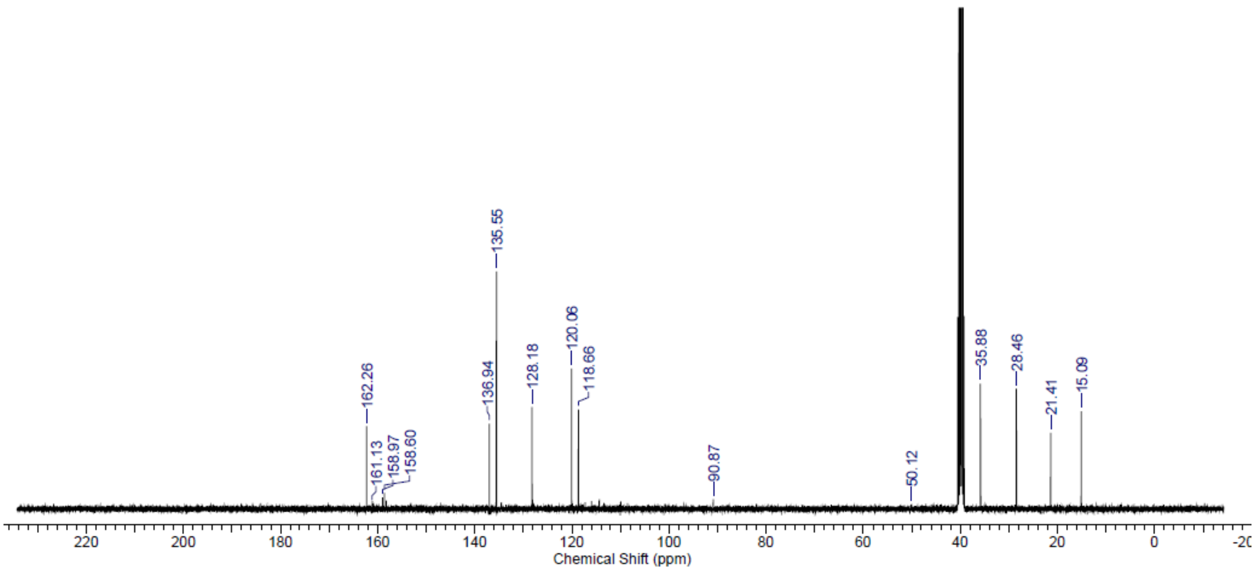

Supplementary Figure 26: ^{13}C NMR (DMSO-d_6) spectrum of 6-1.

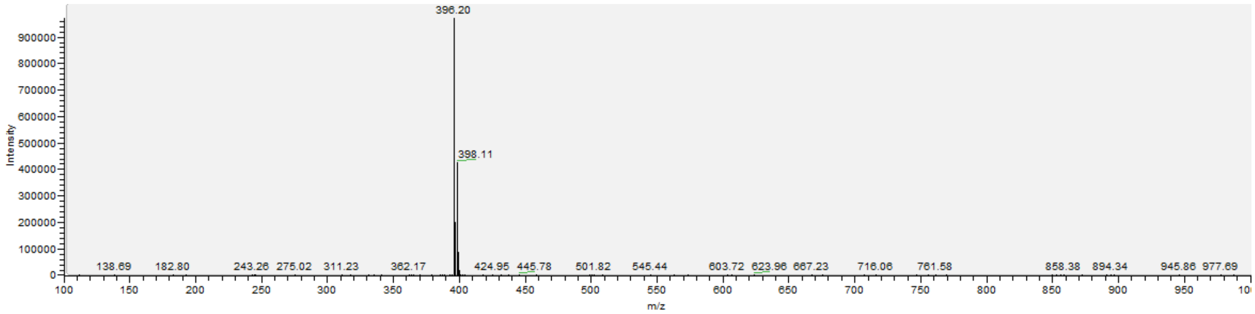

Supplementary Figure 27: ESI MS spectrum of 6-1.

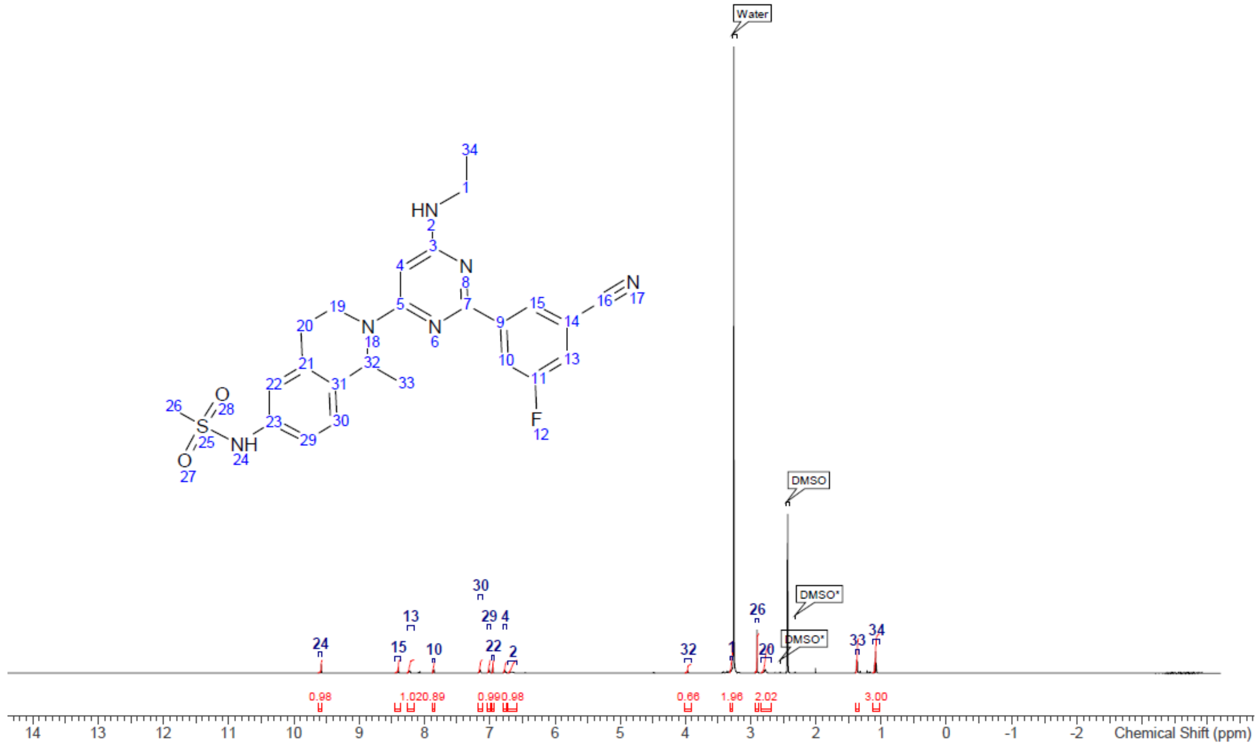

Supplementary Figure 28: ^{1}H NMR (DMSO-d_6) spectrum of 6.

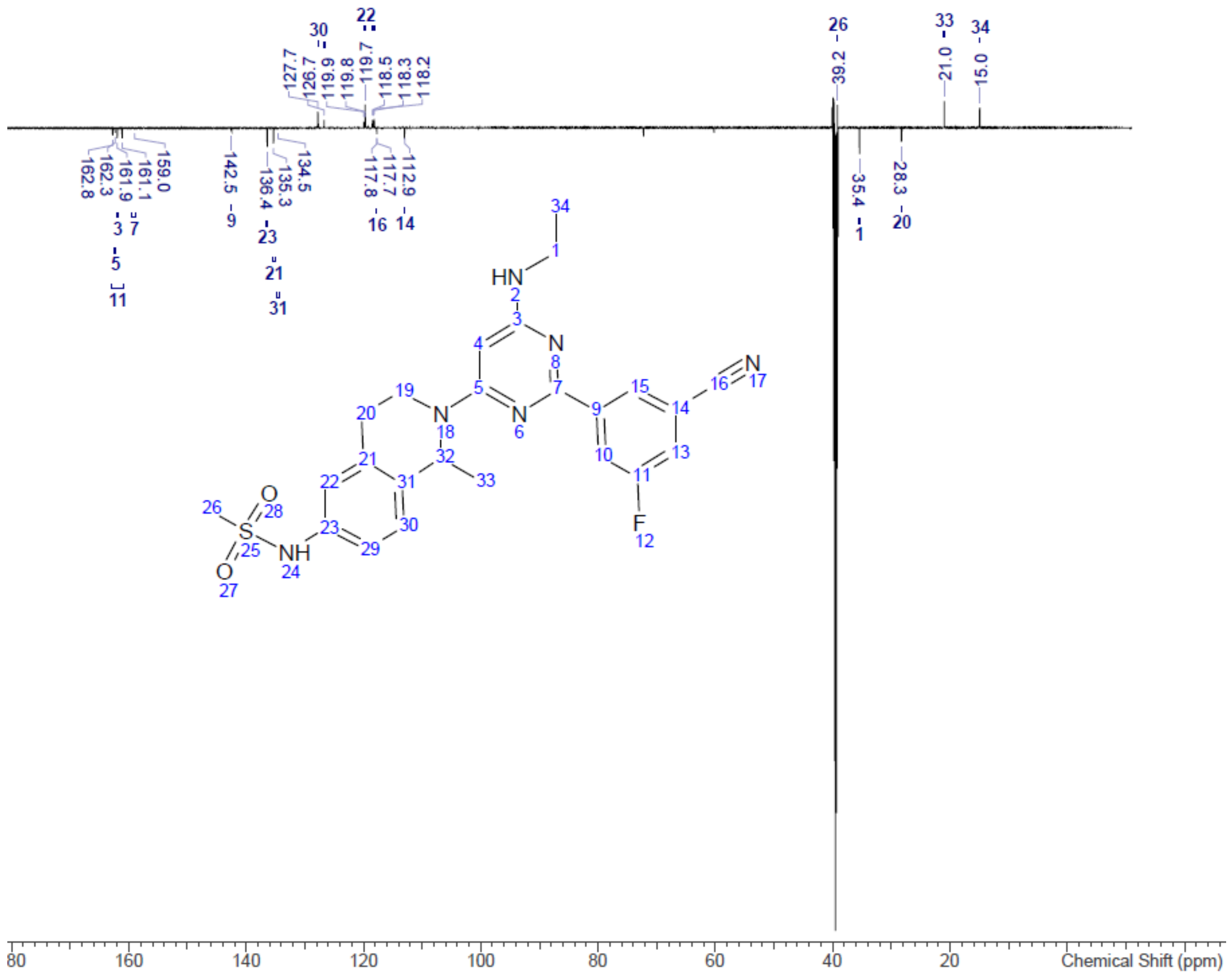

Supplementary Figure 29: ^{13}C NMR (DMSO-d_6) spectrum of 6.

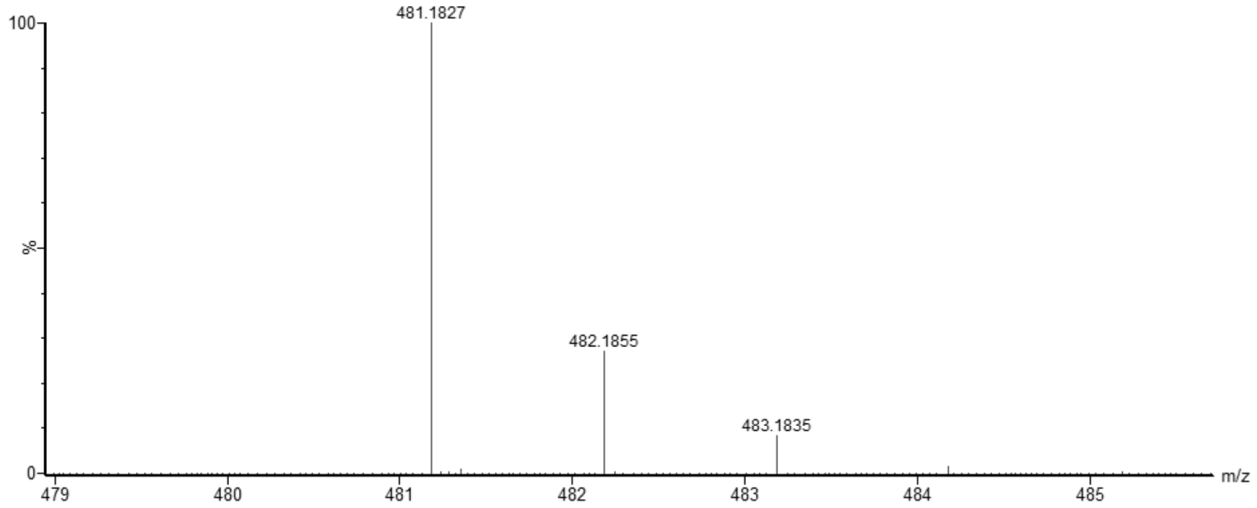

Supplementary Figure 30: HRMS spectrum of 6.

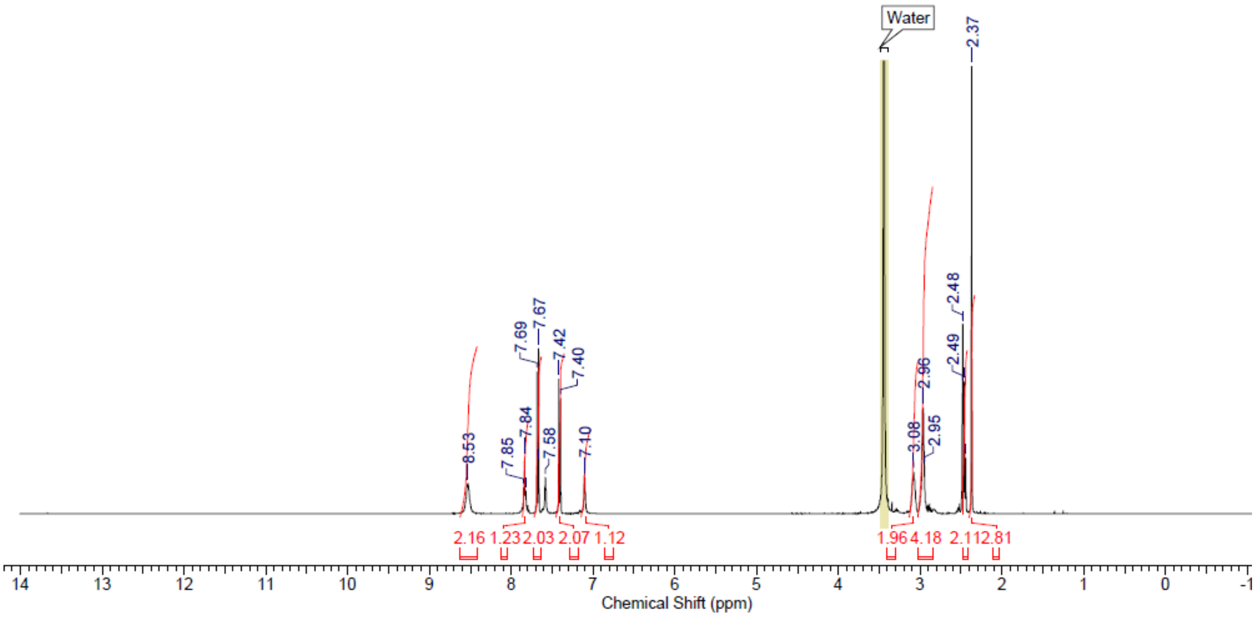

Supplementary Figure 31: ^{1}H NMR (DMSO-d$_6$) spectrum of 7-1.

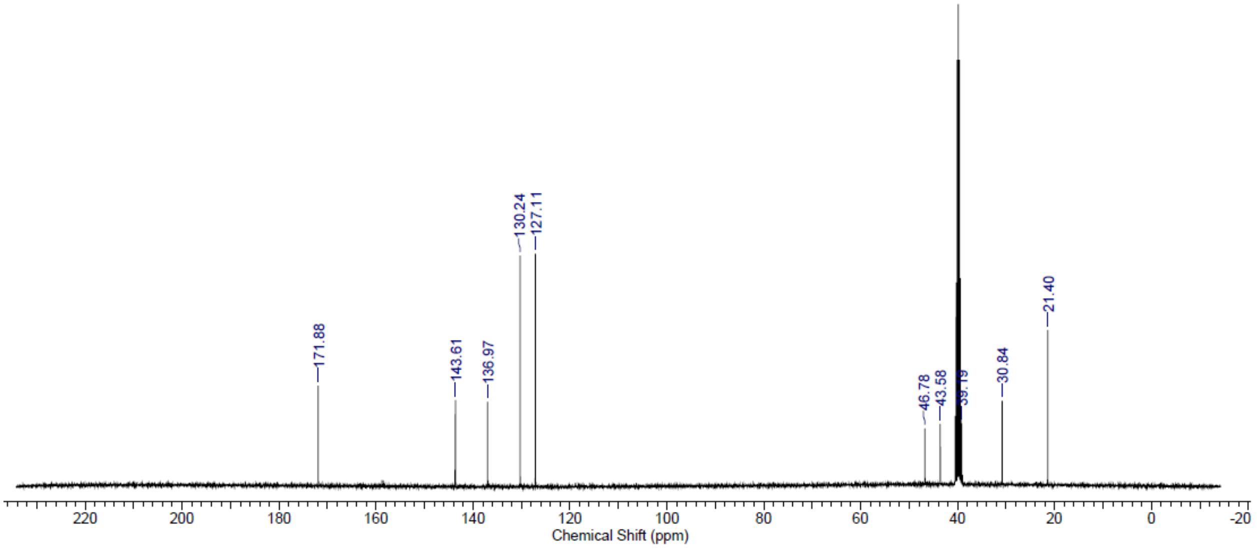

Supplementary Figure 32: ^{13}C NMR (DMSO-d$_6$) spectrum of 7-1.

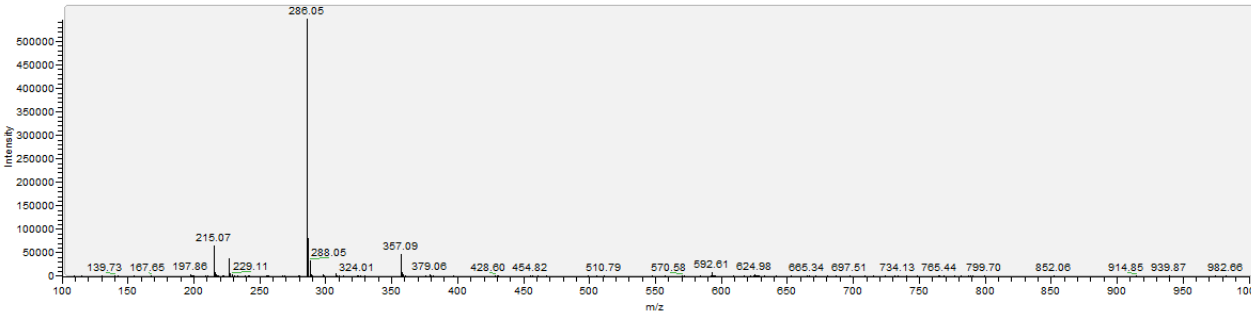

Supplementary Figure 33: ESI MS spectrum of 7-1.

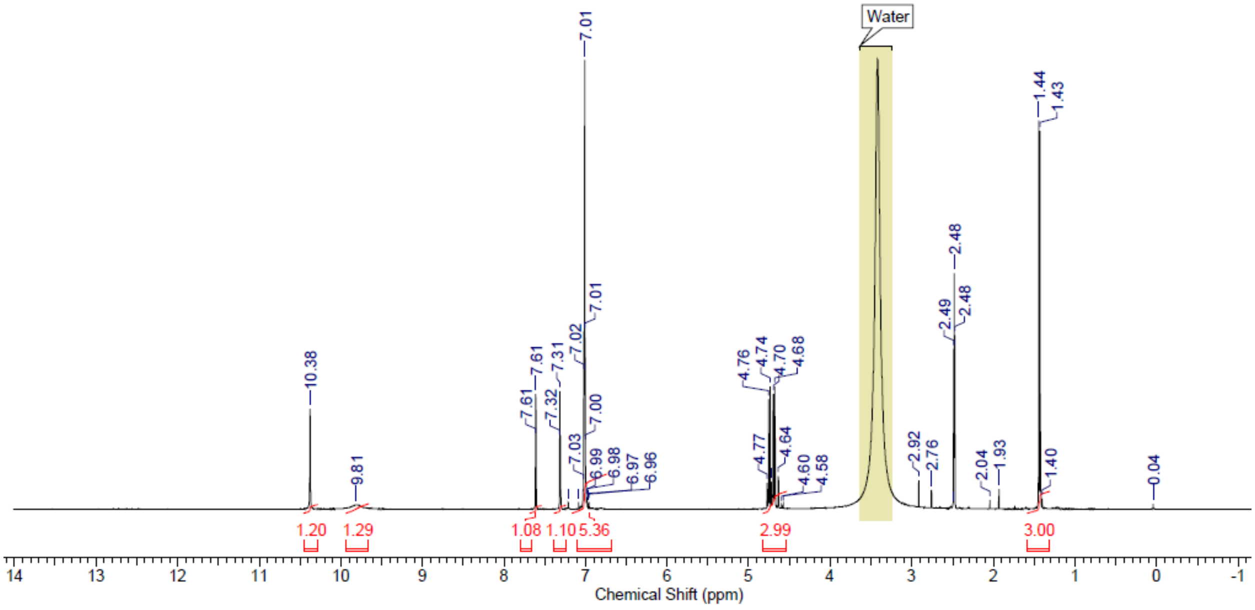

Supplementary Figure 34: ^{1}H NMR (DMSO-d_6) spectrum of 7-2.

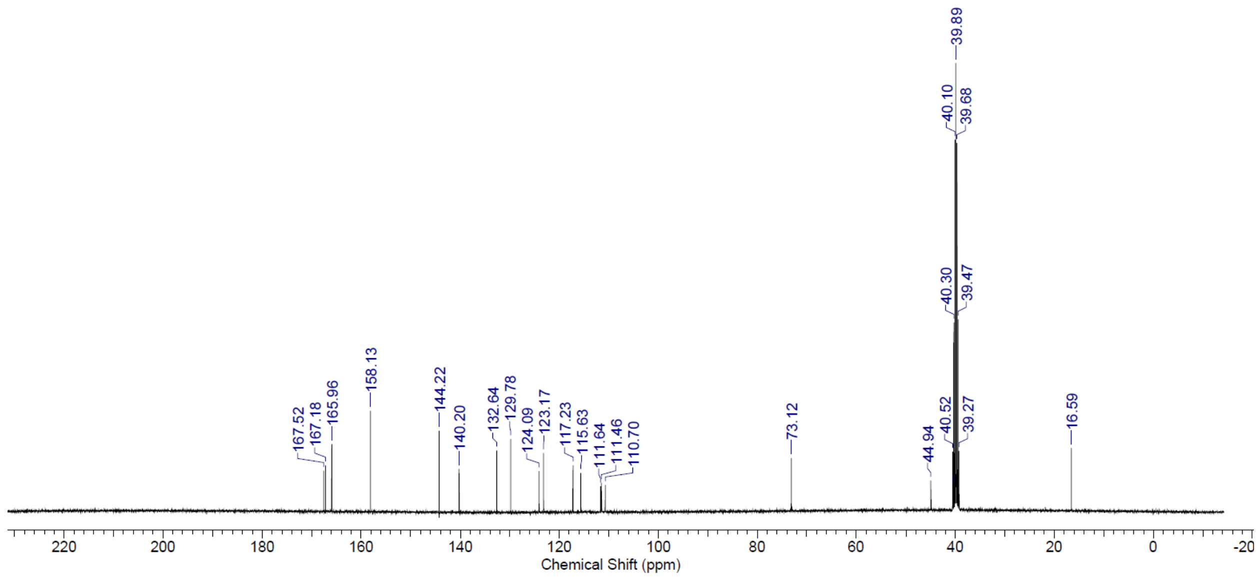

Supplementary Figure 35: ^{13}C NMR (DMSO-d$_6$) spectrum of 7-2.

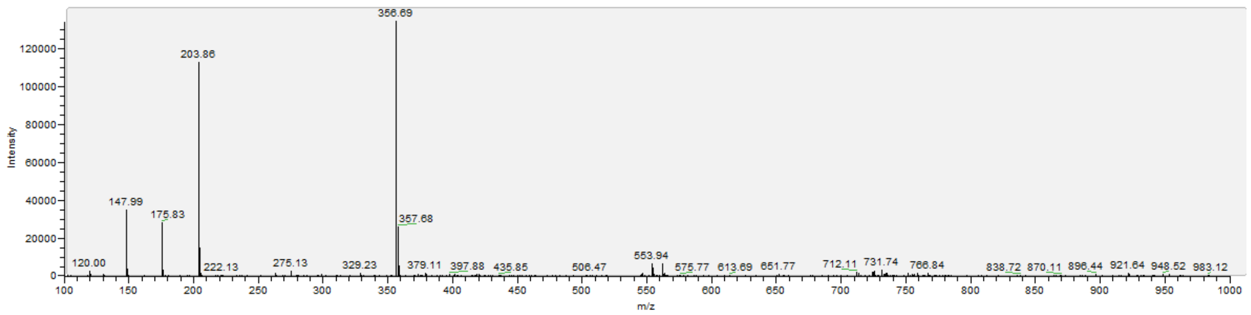

Supplementary Figure 36: ESI MS spectrum of 7-2.

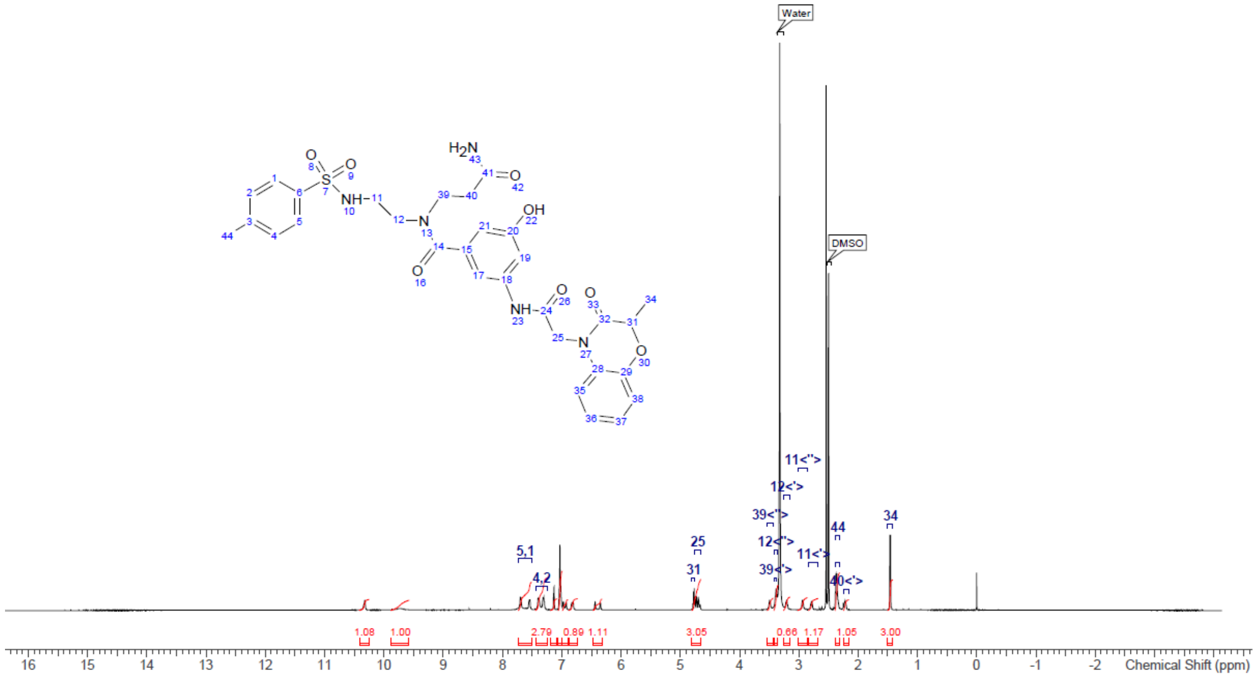

Supplementary Figure 37: ^{1}H NMR (DMSO-d_6) spectrum of 7.

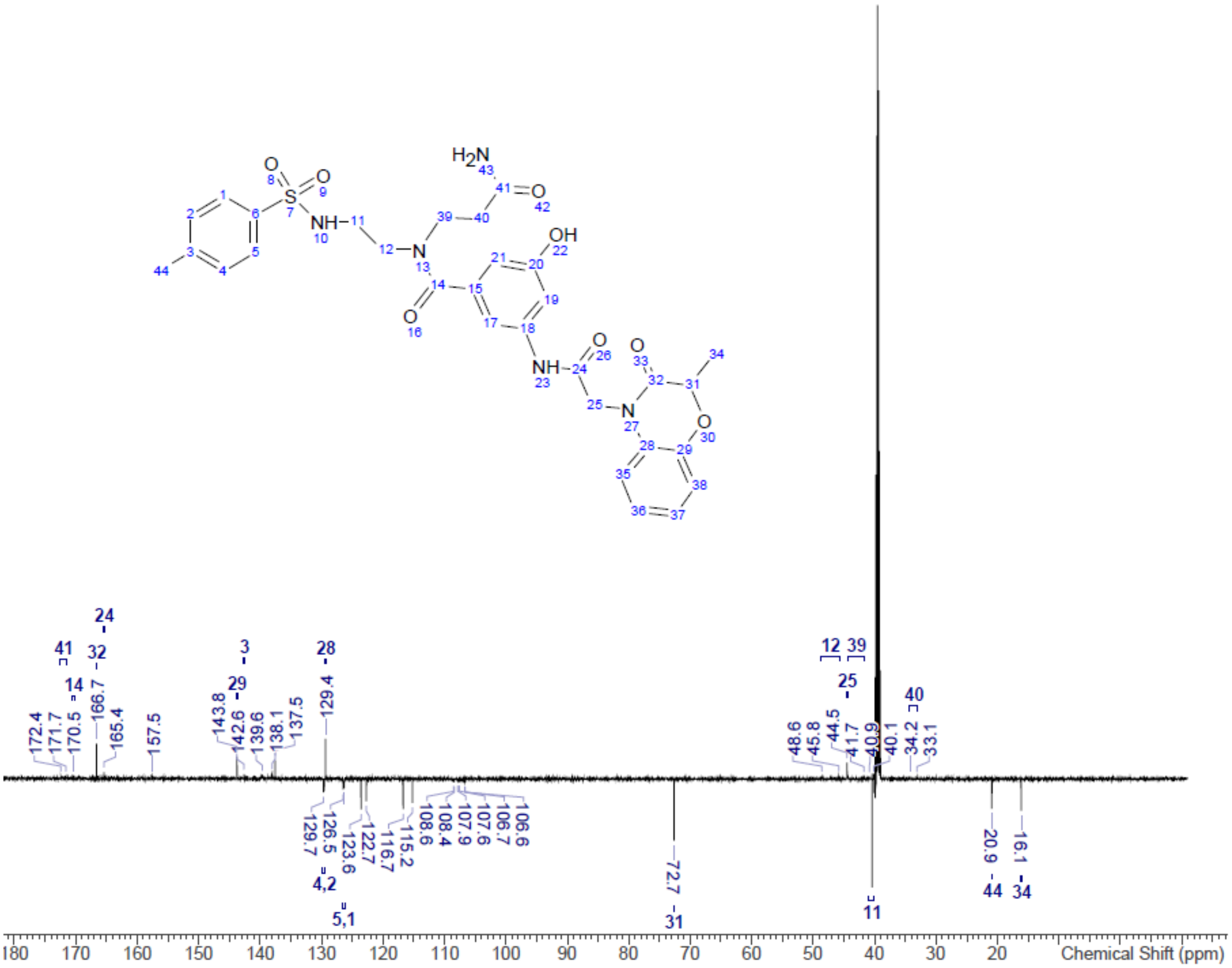

Supplementary Figure 38: ^{13}C NMR (DMSO-d_6) spectrum of 7.

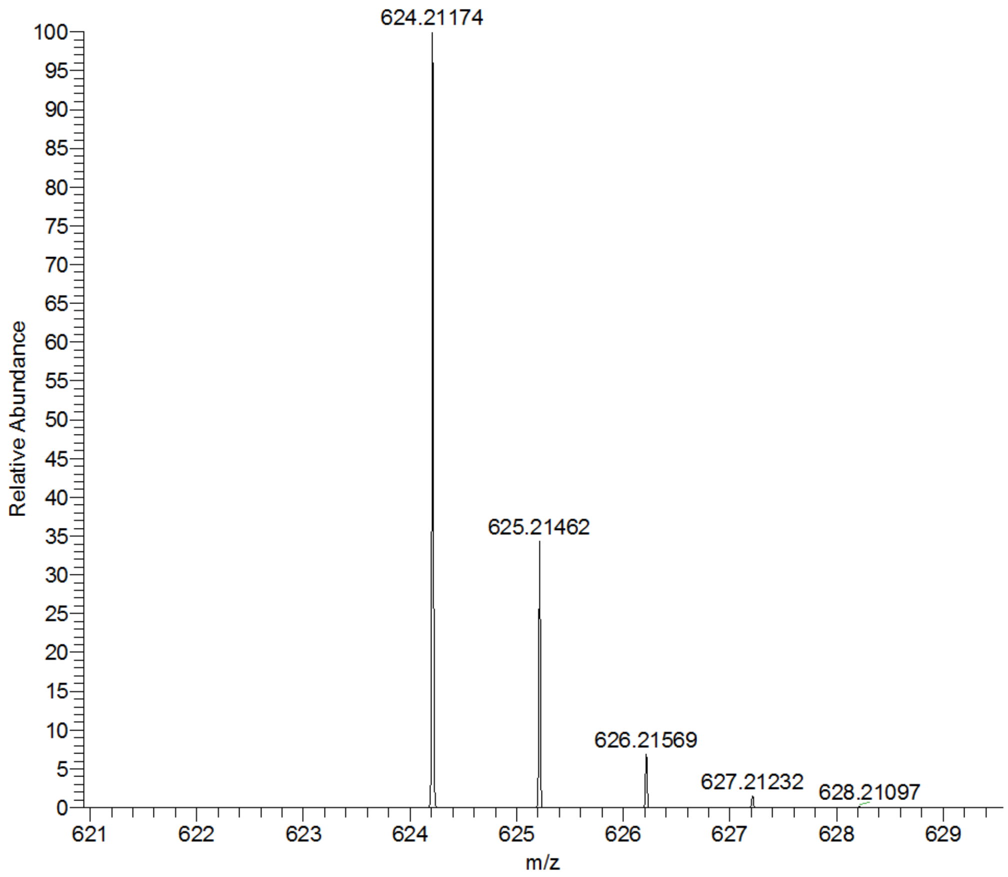

Supplementary Figure 39: HRMS spectrum of 7.

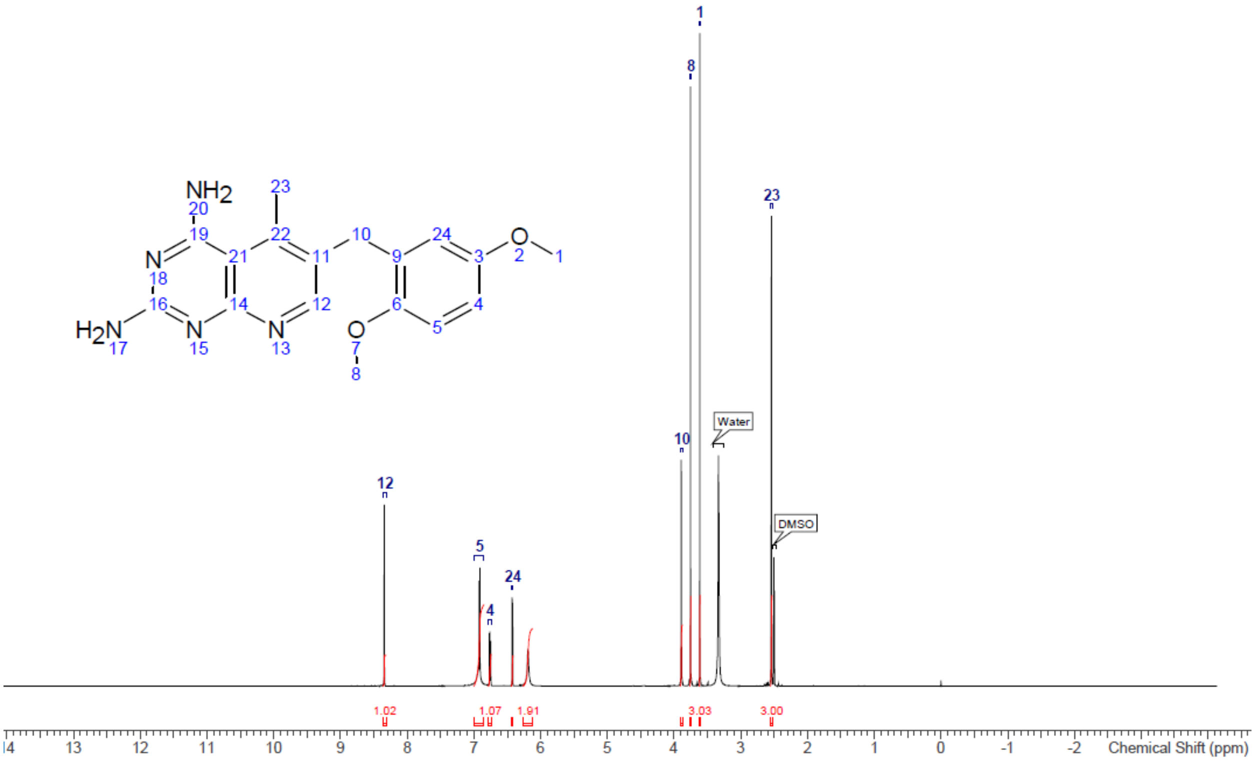

Supplementary Figure 40: ^{1}H NMR (DMSO-d_6) spectrum of 8.

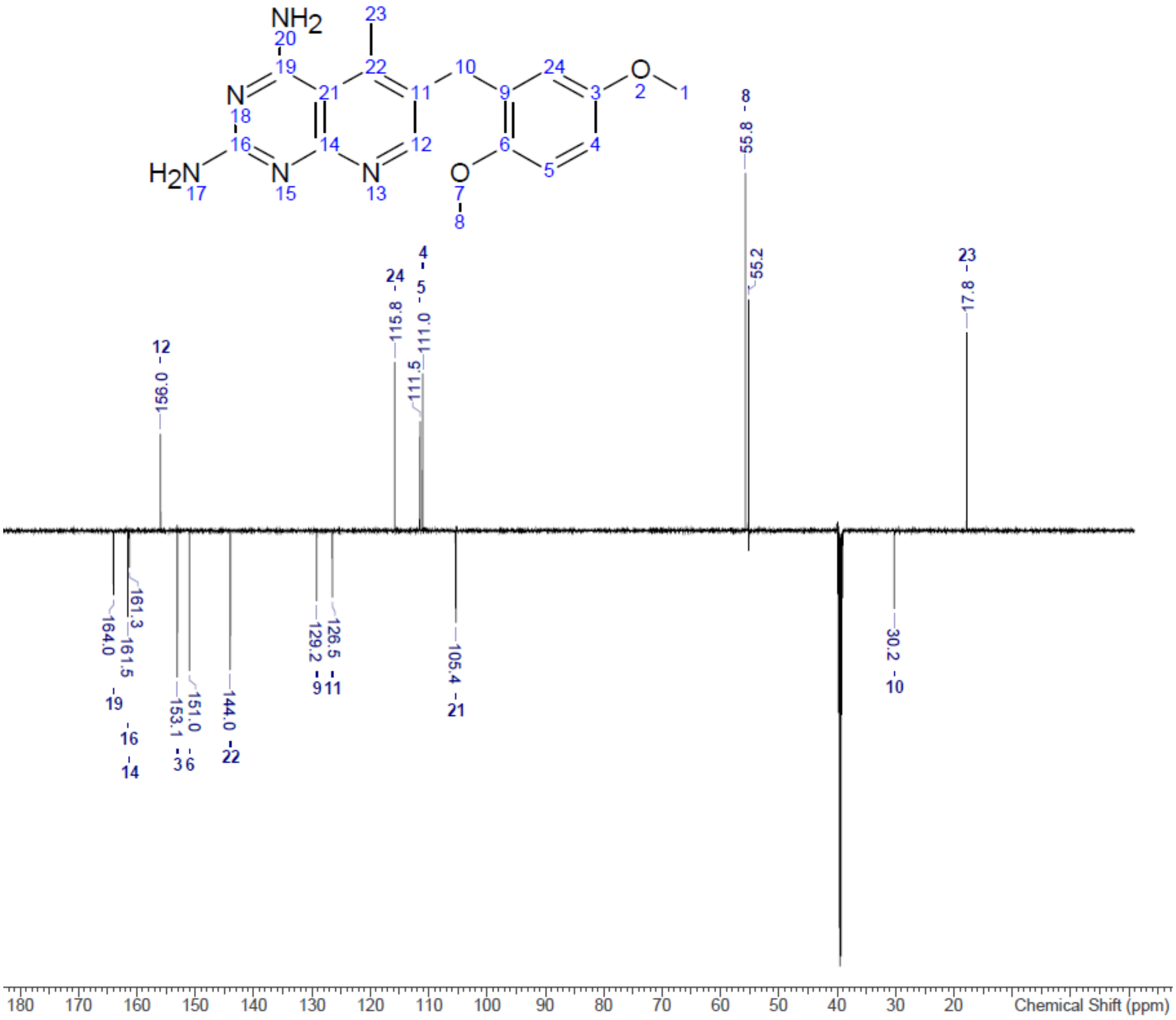

Supplementary Figure 41: ^{13}C NMR (DMSO-d_6) spectrum of 8.

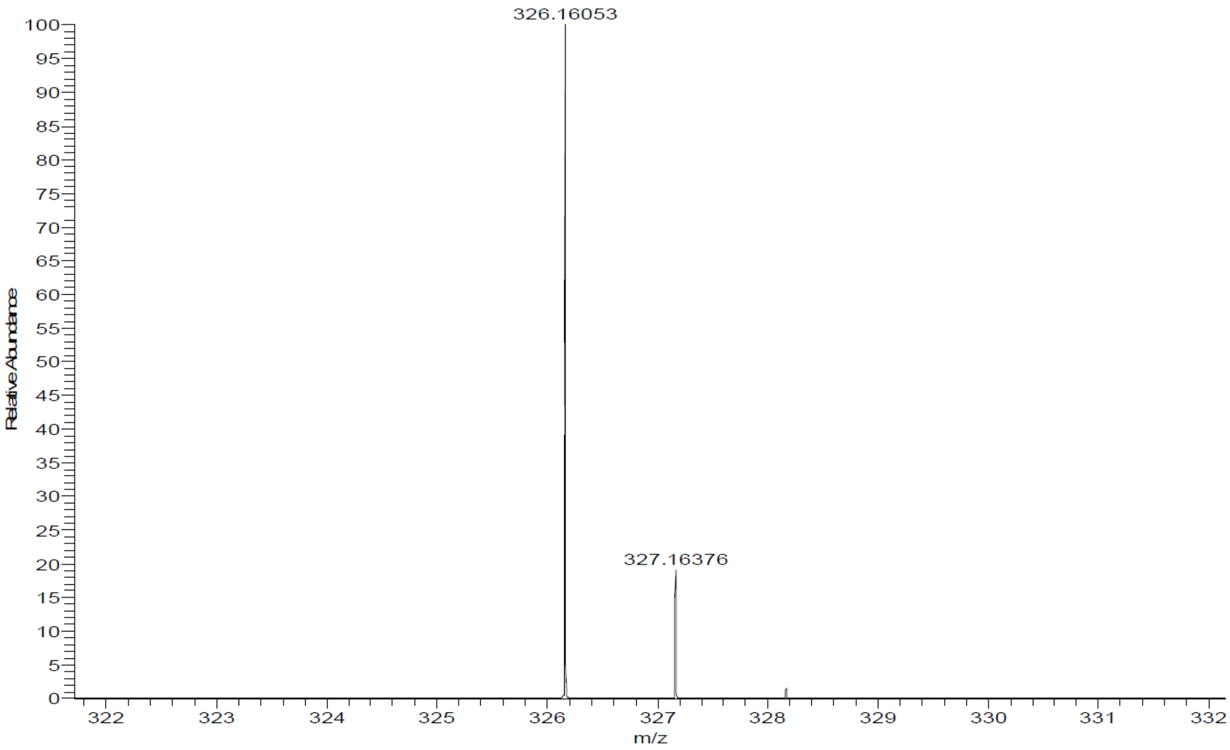

Supplementary Figure 42: HRMS spectrum of 8.

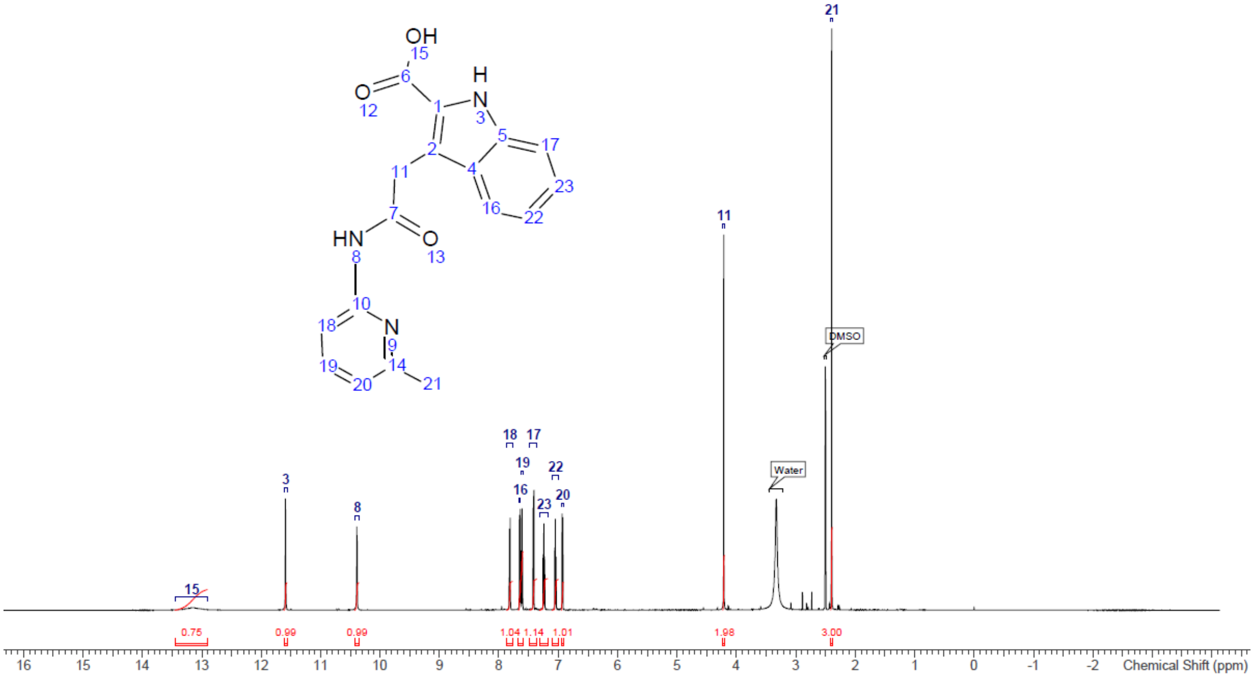

Supplementary Figure 43: ^{1}H NMR (DMSO-d_6) spectrum of 9.

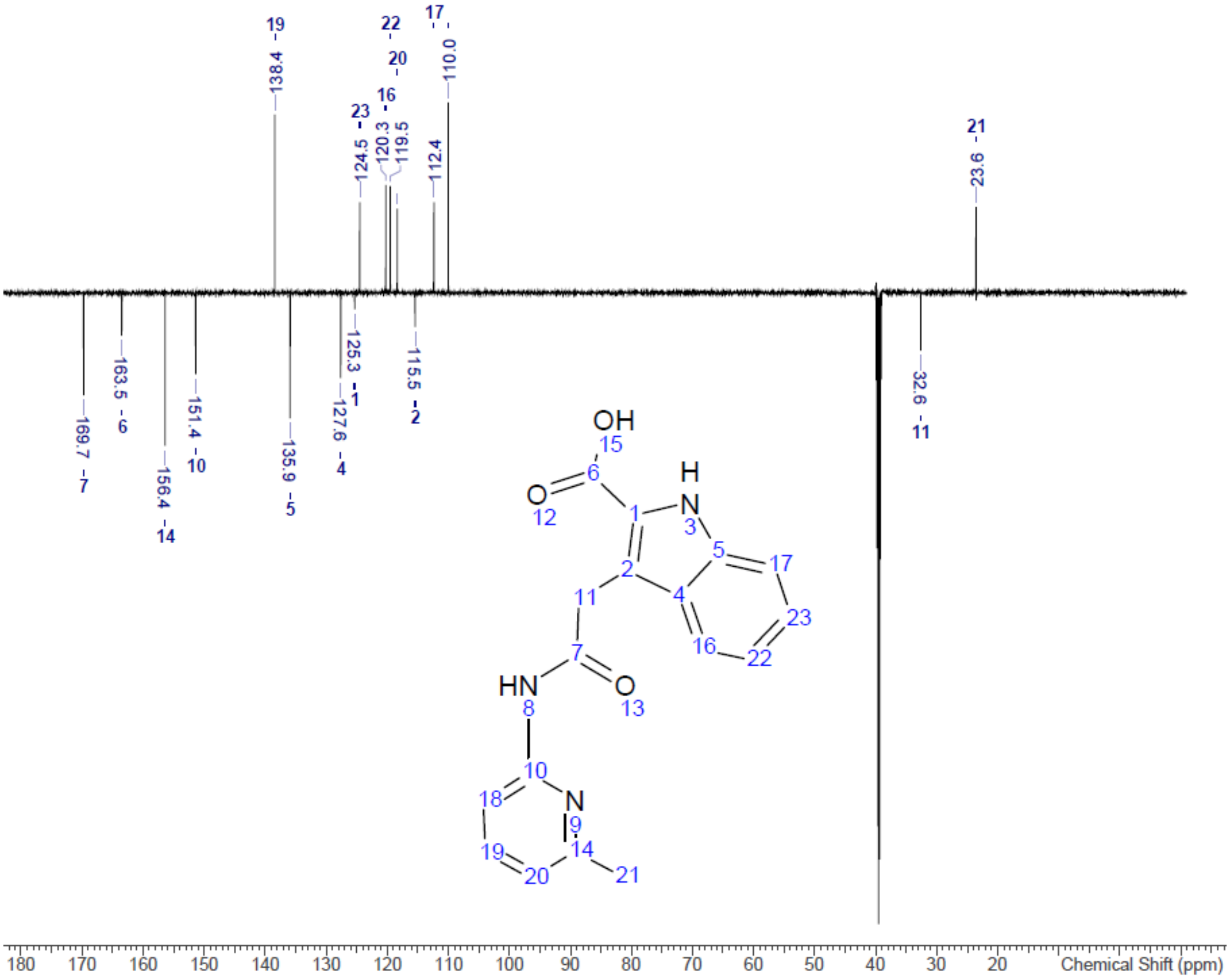

Supplementary Figure 44: ^{13}C NMR (DMSO-d_6) spectrum of 9.

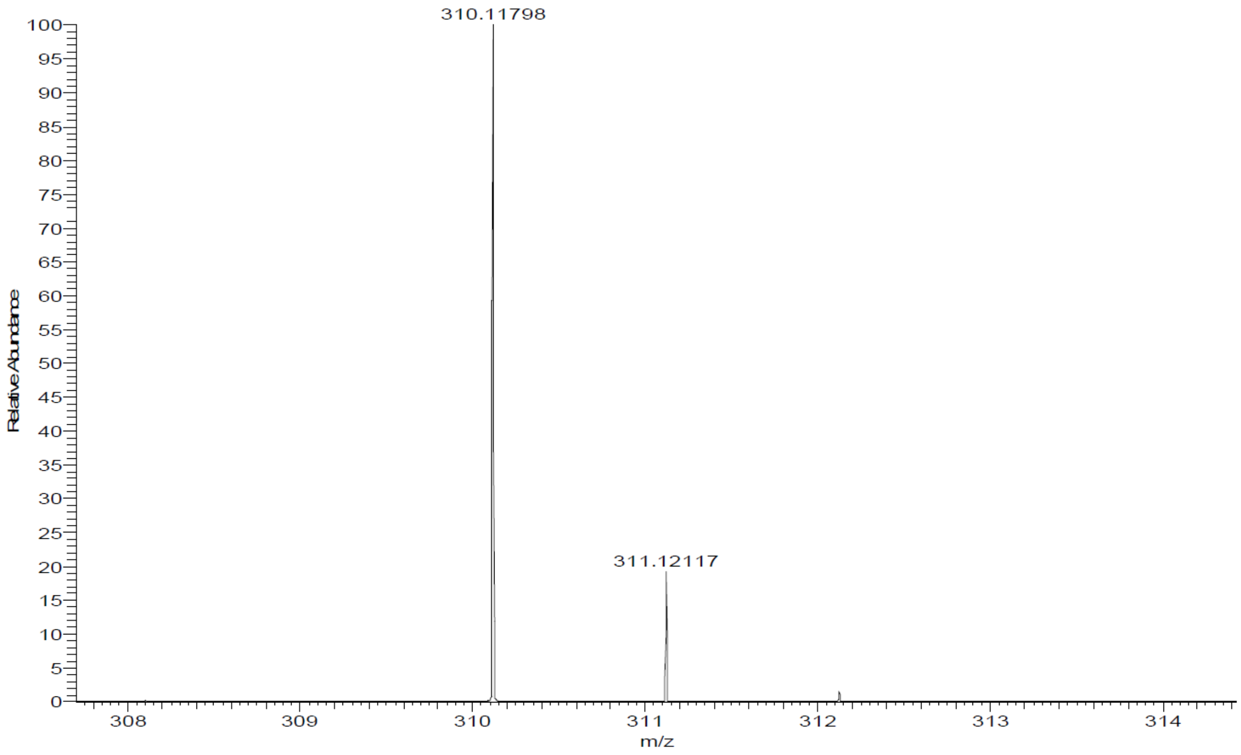

Supplementary Figure 45: HRMS spectrum of 9.

Supplementary Tables:

Supplementary Table 1: Progression of targets through tractability assessment process.

Gene	Protein	ELT signal	Off-DNA synthesis	Discovered active series IC_{50}	MIC	MoA	Compound Disclosed
**A) *Staphylococcus aureus***							
accD/A	Acetyl Co-A carboxylase (ACC)	Y	Y	Y			
acpS	Holo-[acyl-carrier protein] synthase (AcpS)	Y	Y				
alas	Alanyl-tRNA synthetase (ARS)						
birA	Bifunctional biotin-[acetylCoA carboxylase] holoenzyme synthetase (BirA)						
cysS	Cysteinyl-tRNA synthetase (CRS)						
dnaB	Replicative DNA helicase (DnaB)						
dnaE	DNA polymerase III alpha subunit (DnaE)						
asps	Aspartyl-tRNA synthetase (DRS)						
gltX	Glutamyl-tRNA synthetase (ERS)						
fabG	3-oxoacyl-[acyl-carrier-protein] reductase (FabG)						
fabH	3-oxoacyl-(acyl carrier protein) synthase III (FabH)						
pheS/T	Phenylalanyl-tRNA synthetase (FRS)						
glum	N-acetyl glucosamine-1-phosphate uridyltransferase/glucosamine-1-phosphate acetyl transferase (GlmU)						
glyS	Glycyl-tRNA synthetase (GRS)						
Hiss	Histidyl-tRNA synthetase (HRS)	Y	Y				
ileS	Isoleucyl-tRNA synthetase (IRS)	Y	Y	Y	Y		Y
lysS	Lysyl-tRNA synthetase (KRS)	Y	Y				
spsB	Signal peptidase Ib (SpsB)						
leuS	Leucyl-tRNA synthetase (LRS)						
Map	Methionine aminopeptidase (MetAP)	Y	Y	Y			Y
metS	Methionyl-tRNA synthetase (MRS)	Y	Y	Y	Y	Y	Y
murA	UDP-N-acetylglucosamine 1-carboxyvinyltransferase (MurA)	Y	Y				
murB	UDP-N-acetylenolpyruvoylglucosamine reductase (MurB)						

Gene	Protein	ELT signal	Off-DNA synthesis	Discovered active series IC$_{50}$	MIC	MoA	Compound Disclosed
murC	UDP-N-acetylmuramate:L-alanine ligase (MurC)						
asnS	Asparaginyl-tRNA synthetase (NRS)	Y	Y				
PBP-2'	Penicillin-binding protein-2' (PBP-2')						
Def	Peptidyl deformylase (PDF)						
coaD	Phosphopantetheine adenylyltransferase (PPAT)	Y	Y				
pros	Prolyl-tRNA synthetase (PRS)						
Pth	Peptidyl-tRNA hydrolase (Pth)	Y	Y	Y			
pyrH	Uridylate kinase (PyrH)	Y	Y	Y			
RNAP	RNA polymerase (RNAP)						
rnpA	ribonuclease P protein component (Rnase-P)						
serS	seryl-tRNA synthetase (SRS)						
thrS	threonyl-tRNA synthetase (TRS)						
Upps	Undecaprenyl pyrophosphate synthetase UppS	Y	Y	Y	Y	Y	Y[a]
valS	Valyl-tRNA synthetase (VRS)						
trpS	Tryptophanyl-tRNA synthetase (WRS)	Y	Y				
tyrS	Tyrosyl-tRNA synthetase (YRS)						
	**Total**	**14**	**14**	**7**	**3**	**2**	**4**
**B) *Acinetobacter baumannii***							
acpP	Acyl carrier protein (AcpP)	Y	[b]		Y		
bamA	Outer membrane protein assembly factor BamA β-barrel assembly machinery (BamA)	Y					
bamD	Outer membrane protein assembly factor BamD/ β -barrel assembly machinery (BamD)	Y					
birA	Bifunctional biotin-[acetylCoA carboxylase] holoenzyme synthetase (BirA)	Y	Y				
cca	tRNA nucleotidyltransferase (CCA)	Y	Y		Y		
cdsA	Phosphatidate cytidylyltransferase (CdsA)						
dacC	Penicillin-binding protein 5 (DacC)	Y					
ddlb	D-alanine-D-alanine ligase B (DdlB)						
dnaB	Replicative DNA helicase (DnaB)	Y					

Gene	Protein	ELT signal	Off-DNA synthesis	Discovered active series IC$_{50}$	MIC	MoA	Compound Disclosed
dnaE	DNA polymerase III alpha subunit (DnaE)	Y					
dnaG	DNA primase (DnaG)	Y					
dnaX	DNA polymerase III tau and gamma subunits (DnaX)	Y					
dxr	1-deoxy-D-xylulose 5-phosphate reductoisomerase (DXR)	Y	Y	Y	Y		
dxs	1-deoxyxylulose-5-phosphate synthase (DXS)	Y	Y		Y		
engA	Putative GTP-binding protein (EngA)						
engB	Putative GTP-binding protein (EngB)	Y					
fabZ	3-hydroxyacyl-[acyl-carrier-protein] dehydratase (FabZ)	Y	Y		Y		
ftsH	cell division ATP-dependent metalloprotease (FtsH)	Y					
ftsI	transpeptidase involved in septal peptidoglycan synthesis / penicillin-binding protein 3 (FtsI)	Y	Y		Y		
gcp	putative O-sialoglycoprotein endopeptidase Gcp						
glmM	phosphoglucosamine mutase GlmM	Y					
glmS	Glucosamine--fructose-6-phosphate aminotransferase GlmS	Y					
glmU	N-acetyl glucosamine-1-phosphate uridyltransferase/glucosamine-1-phosphate acetyl transferase GlmU						
grpE	heat shock protein 24 nucleotide exchange factor/heat shock protein GrpE						
guaB	inositol-5-monophosphate dehydrogenase (GuaB)						
ispA	Farnesyl diphosphate synthase (IspA)	Y					
ispB	Octaprenyl diphosphate synthase (IspB)	Y					
ispD	4-diphosphocytidyl-2-C-methyl-D-erythritol synthase (IspD)	Y					
ispF	2-C-methyl-D-erythritol 2,4-cyclodiphosphate synthase (IspF)	Y	Y		Y		

Gene	Protein	ELT signal	Off-DNA synthesis	Discovered active series		MoA	Compound Disclosed
				IC_{50}	MIC		
ispG	4-hydroxy-3-methylbut-2-en-1-yl diphosphate synthase (IspG)	Y	Y		Y		
ispH	4-hydroxy-3-methylbut-2-enyl diphosphate reductase (IspH)	Y					
kdtA	3-deoxy-D-manno-2-octulosonate transferase (KdtA)						
lepB	Signal peptidase I (LepB)	Y					
lolA	Outer membrane lipoproteins carrier protein (LolA)	Y	Y		Y	Y	Y
lolB	Outer membrane lipoprotein required for localization of lipoproteins (LolB)						
lolD	Lipoprotein releasing system ATP-binding protein (LolD)						
lptA	Periplasmic LPS-binding protein (LptA)						
lptB	ABC transporter ATP-binding protein (LptB)						
lpxA	UDP-acetylglucosamine acyltransferase (LpxA)	Y	Y		Y	Y	Y
lpxB	Lipid A-disaccharide synthase (LpxB)	Y					
lpxD	UDP-3-O-[3-hydroxylauroyl] glucosamine N-acyltransferase (LpxD)	Y	Y		Y		
lpxH	UDP-2,3-diacylglucosamine hydrolase (LpxH)	Y	Y				
lpxK	Tetraacyldisaccharide 4'-kinase /Lipid A 4'-kinase) (LpxK)	Y					
map	Methionine aminopeptidase (MetAP)	Y					
metK	S-adenosylmethionine synthetase (MetK)	Y					
mraY	Phospho-N-acetylmuramoyl-pentapeptide transferase (MraY)						
mrcB	PBP1b, transglycosylase and transpeptidase (MrcB)	Y	Y		Y		
msbA	Lipid A export ATP-binding/permease protein (MsbA)						
mtgA	Monofunctional biosynthetic peptidoglycan transglycosylase (MtgA)						
murA	UDP-N-acetylglucosamine 1-carboxyvinyltransferase (MurA)	Y					

Gene	Protein	ELT signal	Off-DNA synthesis	Discovered active series IC$_{50}$	MIC	MoA	Compound Disclosed
murB	UDP-N-acetylenolpyruvoylglucosamine reductase (MurB)	Y					
murC	UDP-N-acetylmuramate-L-alanine ligase (MurC)	Y					
murD	UDP-N-acetylmuramoylalanine-D-glutamate ligase (MurD)	Y					
murE	UDP-N-acetylmuramoylalanyl-D-glutamate-2, 6-diaminopimelate ligase (MurE)	Y					
murF	UDP-N-acetylmuramoyl-tripeptide-D-alanyl-D-alanine ligase (MurF)	Y					
murG	UDP-N-acetylglucosamine--N-acetylmuramyl-(Pentapeptide) pyrophosphoryl-undecaprenol N-acetylglucosamine transferase (MurG)	Y	Y		Y		
murI	Glutamate racemase (MurI)	Y					
murJ	Probable peptidoglycan lipid II flippase (MurJ)						
nrdA	Ribonucleoside diphosphate reductase, alpha subunit (NrdA)	Y					
nrdB	Ribonucleoside-diphosphate reductase, beta subunit (NrdB)						
obgE	putative GTP-binding protein (ObgE)	Y					
pbpA	Penicillin-binding protein 2 (PbpA)	Y	Y		Y		
pgsA	CDP-diacylglycerol--glycerol-3-phosphate 3-phosphatidyltransferase (PgsA)						
ponA	PBP1a, transglycosylase and transpeptidase (PonA)						
prfB	Peptide chain release factor 2 (PrfB)	Y					
pth	Peptidyl-tRNA hydrolase (Pth)	Y	Y		Y		
pyrH	Uridylate kinase (PyrH)	Y					
rho	Transcription termination factor (Rho)	Y					
rnpA	Ribonuclease P protein component (RnpA)	Y					
thyA	Thymidylate synthase (ThyA)	Y	Y		Y		
uppS	Undecaprenyl pyrophosphate synthetase (UppS)	Y	Y	Y	Y	Y	Y

Gene	Protein	ELT signal	Off-DNA synthesis	Discovered active series IC_{50}	Discovered active series MIC	MoA	Compound Disclosed
	**Total**	**52**	**18**	**2**	**17**	**3**	**3**
**C) *Mycobacterium tuberculosis***							
accA3	bifunctional acetyl-/propionyl-coenzyme A carboxylase alpha chain (AccA3): biotin carboxylase + biotin carboxyl carrier protein (BCCP)	Y	Y				
accD4	Propionyl-CoA carboxylase beta chain 4 AccD4	Y	Y				
accD5	Propionyl-CoA carboxylase beta chain 5 AccD5	Y	Y				
accD6	Acetyl/propionyl-CoA carboxylase (beta subunit) AccD6	Y					
aftA	Arabinofuranosyltransferase (AftA)	Y					
aspS	Aspartyl-tRNA synthetase (AspS)	Y					
birA	Bifunctional biotin operon repressor and biotin--[acetyl-CoA-carboxylase] synthetase (BirA)						
dapE	Probable succinyl-diaminopimelate desuccinylase (DapE)						
dapF	Diaminopimelate epimerase (DapF)						
dxr	1-deoxy-D-xylulose 5-phosphate reductoisomerase (DXR)	Y	Y	Y			
dfrA	Dihydrofolate reductase DfrA (DHFR)	Y	Y	Y			Y
echA6	Enoyl-CoA hydratase (EchA6)	Y	Y				
egtD	Histidine-specific methyltransferas (EgtD/ Rv3701c)						
embB	Integral membrane indolylacetylinositol arabinosyltransferase EmbB (arabinosylindolylacetylinositol synthase)						
embC	Integral membrane indolylacetylinositol arabinosyltransferase EmbC (arabinosylindolylacetylinositol synthase)						
fasR	fatty acid synthesis Regulator (FasR)	Y					
fbpA	Secreted antigen 85-a FbpA (mycolyl transferase 85A) (fibronectin-binding protein A) (antigen 85 complex A)	Y	Y				

Gene	Protein	ELT signal	Off-DNA synthesis	Discovered active series IC$_{50}$	MIC	MoA	Compound Disclosed
fbpC2	Secreted antigen 85-C FbpC (85C) (antigen 85 complex C) (AG58C) (mycolyl transferase 85C) (fibronectin-binding protein C)	Y	Y				
glfT2	Bifunctional UDP-galactofuranosyl transferase (GlfT2)	Y					
glmU	UDP-N-acetylglucosamine pyrophosphorylase (GlmU)	Y					
guaB2	Inosine-5'-monophosphate dehydrogenase (GuaB2)						
inhA	InhA control	Y					
kasA	3-oxoacyl-[acyl-carrier protein] synthase 1 KasA (beta-ketoacyl-ACP synthase)	Y	Y	Y			
kasB	3-oxoacyl-[acyl-carrier protein] synthase 2 KasB (beta-ketoacyl-ACP synthase)						
leuS	Leucyl-tRNA synthetase (LeuS)						
lpdC	Dihydrolipoamide dehydrogenase (LpdC)	Y	Y	Y			
mabR	Mycolic acid biosynthesis regulator (MabR/ Rv2242)	Y	Y				
mapA	Methionine aminopeptidase MapA (MetAP)	Y					
mapB	Methionine aminopeptidase MapB						
murC	UDP-N-acetylmuramate-alanine ligase (MurC)						
murI	Glutamate racemase (MurI)	Y					
panC	Pantothenate synthetase (PanC)	Y					
pimB	Mannosyltransferase (PimB)						
pknB	Serine/threonine-protein kinase B (PknB)	Y					
pks13	Polyketide synthase (Pks13)	Y					
ppm1	Polyprenol-monophosphomannose synthase (Ppm1)						
MptpA	Mycobacterial Phosphotyrosine protein phosphatase (PtpA)	Y	Y				

Gene	Protein	ELT signal	Off-DNA synthesis	Discovered active series IC_{50}	MIC	MoA	Compound Disclosed
MptpB	Mycobacterial Phosphotyrosine protein phosphatase (PtpB)	Y	Y				
Rv3267	Conserved protein (CPSA-related protein) Rv3267	Y					
sahH	Adenosylhomocysteinase (SahH)						
topA	DNA topoisomerase I (TopA)	Y					
trxB2	Thioredoxin reductase (TrxB2)						
trxC	Thioredoxin reductase (TrxC)	Y					
	**Total**	**27**	**13**	**4**	**-**	**-**	**1**

This table shows the detailed progression of individual targets for each screening campaign. The column headers are described as follows. ELT signal: target had specific binders from ELT screen; Prioritized for off-DNA synthesis: target was chosen for follow-up with chemistry efforts on 3-5 chemotypes; Discovered active series: target had compound with measurable activity (IC_{50} measurement for *S. aureus* and *M. tuberculosis*, MIC measurement for *A.baumannii*); MoA: target where measured activity was demonstrated to be likely through the target. Targets that were not amenable to ELT screening in *A. baumannii* panel: *ffH, htrB, lolC, lolE, lptF, lptG, ostA, uppP, ybjG*.

a) Details of UppS screening are reported elsewhere[6].

b) Compound found through similarity search against corporate collection.

c) InhA as a control target was not included in the count of total number of targets with ELT signal.

Supplementary Table 2: Panel of bacterial strains used to test *A. baumannii* ELT hits.

Bacterial Strain	Description	Compound MIC, µg/ml		
		4	5	6
Escherichia coli 7623 Δ*tolC*	Efflux mutant	>128	>128	>128
Klebsiella pneumoniae 1161486a Δ*tolC*	Efflux mutant	>128	>128	>128
Pseudomonas aeruginosa PA0322 Δ(*mexAB-oprM*) Δ(*mexCD-oprJ*) Δ(*mexEF-oprN*)	Efflux mutant	>128	>128	>128
Acinetobacter baumannii BM4652 Δ*adeABC* Δ*adeIJK*	Efflux mutant	>128	>128	>128
Acinetobacter baumannii ATCC 19606-1 ΔLpxC	LPS mutant	>128	$\leq$ 0.125	32
Haemophilus influenzae H128 Δ*acrB*	Efflux mutant	NT	1	2[b]
Staphylococcus aureus RN4220	Gram-positive	>128	>128[a]	>128

Reported values were observed in a minimum of two replicate experiments.
a) *S. aureus* WCUH29, b) *H. influenzae* Δ*tolC*

Supplementary Table 3: Comparison of *S. aureus* targets screened by both ELT and HTS.

Gene	Protein	ELT Outcome	HTS Outcome
accD/A	Acetyl Co-A carboxylase (ACC)	Prioritized[a]	Hits found
Map	Methionine aminopeptidase (MetAP)	Prioritized[a]	Hits found
metS	Methionyl-tRNA synthetase (MRS)	Prioritized[a,b]	Hits found[b]
trpS	Tryptophanyl-tRNA synthetase (WRS)	Prioritized	Hits Found
Hiss	Histidyl-tRNA synthetase (HRS)	Prioritized	No Hits
ileS	Isoleucyl-tRNA synthetase (IRS)	Prioritized[a]	No Hits
lysS	Lysyl-tRNA synthetase (KRS)	Prioritized	No Hits
asnS	Asparaginyl-tRNA synthetase (NRS)	Prioritized	No Hits
Upps	Undecaprenyl pyrophosphate synthetase UppS	Prioritized[a,b]	No Hits
Def	Peptidyl deformylase (PDF)	No Signal	Hits Found[b]
valS	Valyl-tRNA synthetase (VRS)	No Signal	Hits Found[b]
tyrS	Tyrosyl-tRNA synthetase (YRS)	No Signal	Hits Found
Alas	Alanyl-tRNA synthetase (ARS)	No Signal	No Hits
birA	Bifunctional biotin-[acetylCoA carboxylase] holoenzyme synthetase (BirA)	No Signal	No Hits
cysS	Cysteinyl-tRNA synthetase (CRS)	No Signal	No Hits

Gene	Protein	ELT Outcome	HTS Outcome
dnaB	Replicative DNA helicase (DnaB)	No Signal	No Hits
dnaE	DNA polymerase III alpha subunit (DnaE)	No Signal	No Hits
Asps	Aspartyl-tRNA synthetase (DRS)	No Signal	No Hits
gltX	Glutamyl-tRNA synthetase (ERS)	No Signal	No Hits
Glum	N-acetyl glucosamine-1-phosphate uridyltransferase/glucosamine-1-phosphate acetyl transferase (GlmU)	No Signal	No Hits
glyS	Glycyl-tRNA synthetase (GRS)	No Signal	No Hits
spsB	Signal peptidase Ib (SpsB)	No Signal	No Hits
leuS	Leucyl-tRNA synthetase (LRS)	No Signal	No Hits
PBP-2’	Penicillin-binding protein-2’ (PBP-2’)	No Signal	No Hits
Pros	Prolyl-tRNA synthetase (PRS)	No Signal	No Hits
RNAP	RNA polymerase (RNAP)	No Signal	No Hits
rnpA	ribonuclease P protein component (Rnase-P)	No Signal	No Hits
serS	seryl-tRNA synthetase (SRS)	No Signal	No Hits
thrS	threonyl-tRNA synthetase (TRS)	No Signal	No Hits

a) ELT targets where activity was confirmed by off-DNA synthesis and IC_{50} measurement. b) Targets where antibacterial activity was confirmed and demonstrated through mechanism of action (MoA) studies. For HTS comparison see Payne *et al.* article[7].

Supplementary Table 4: MIC of teicoplanin (µg/ml) vs *A. baumannii* BM4652 strains

	**MIC of teicoplanin (µg/ml) vs *A. baumannii* BM4652 strains**[a]						
LpxA ELT hit compound **4**	0	4	8	16	32	64	128
A. baumannii BM4652	>64	>64	>64	16	**0.5**	**0.5**	**0.5**
A. baumannii BM4652/pRK415	>64	>64	>64	>64	**0.5**	**0.5**	**0.5**
A. baumannii BM4652/pRK415-LpxA	>64	>64	>64	>64	>64	>64	>64

a) Determined in the presence of different concentrations of LpxA compound **4** ranging from 0 to 128 µg/ml.

Supplementary Table 5: LpxA ELT hit activity (µg/ml)

	LpxA ELT hit activity (µg/ml) Compound **4**	
A. baumannii	MIC	MGIC
BM4652 (efflux-)	>128	8
BM4652/pRK415 (efflux-)	>128	8
BM4652/pRK415-LpxA (efflux-)	>128	>128

Supplementary Table 6: Compound 5 antibacterial mechanism of action.

**Compound**	**MIC (ug/ml) vs *A. baumannii* BM4652 efflux mutant[a]**			**Fold MIC increase**
	N/A	+ pRK415	+ pRK415-UppS	
**5**	0.25	0.25	2	8
Ciprofloxacin	1	1	1	1
Ceftazidime	0.5	0.5	0.5	1
Azithromycin	0.031	0.031	0.031	1

a) Determined in the presence of 30 µg/ml polymyxin B nonapeptide.

Supplementary Table 7: Compound 6 antibacterial mechanism of action.

**Compound**	**MIC (µg/ml) *E. coli TOP10* ΔTolC**		**Fold MIC decrease**
	**+ pHN678'**	**+ pHN678'- LolA antisense**	
**6**	128	4	32
Imipenem	1	1	-
Ciprofloxacin	0.0078	0.0078	-
Azithromycin	0.0156	0.0039	4

<u>Supplementary Notes:</u>

Supplementary Note 1: Cellular Confirmation of Compound Mode of Action.

S. aureus ELT compounds:

The antimicrobial mode-of-action (MoA) of compound **1** identified as a binder of MRS was investigated using an MRS overexpressor strain made from an open reading frame (ORF) expression library of the *S. aureus* genome[1,2] The measured MICs of compound **1** were 0.5, 4 and 64 µg/ml in the *S. aureus* RN4220 strains transformed with pYH4 vector alone, pYH4-MRS overexpressor (uninduced) and pYH4-MRS (induced with 0.1 µg/ml anhydrotetracycline), respectively. Hence, compound **1** demonstrated significant MIC increases of 8 (uninduced) and > 128-fold (induced) in the MRS overexpressors compared to the vector control. These data combined with the reported IC_{50} of 0.00083 µM in **Table 2**, and described below, are consistent with on-target compound activity**.**

IRS compounds were tested for MoA using a *S. aureus* IRS overexpressor strain but no MIC increases were observed compared to isogenic parent strain hence IRS compound antibacterial MoAs were not confirmed. The MetAP ELT compound **3** lacked *S. aureus* antibacterial activity and could not be tested for MoA using a *S. aureus* MetAP overexpressor strain.

A. baumannii ELT compounds:

As *lpxA* has been shown to be not essential for *A. baumannii* viability in growth media, the LpxA ELT hits would not be expected to have MIC against this pathogen[3]. However, *lpxA* null mutants showed severely impaired growth that is clearly distinct from the wild type growth on MIC microtiter test plates. When testing for MIC against *A. baumannii* efflux mutant strain BM4652, we identified several LpxA ELT hits including **4** which exhibited this impaired growth. The antibacterial MoA of **4** was firmly established to be due to inhibition of LpxA by three methods: First, compound **4** showed a growth inhibition (we defined as MGIC for minimum growth inhibition concentration) starting at ~8 to 64 µg/ml although it exhibited no MIC (MIC is >128 µg/ml) against the same strain. Second, the inhibitory mode-of-action of LpxA ELT compound **4** was further verified by its ability to potentiate teicoplanin, a gram positive antibacterial agent that is inactive against gram negative bacteria because it cannot penetrate the outer membrane[3]. We reasoned that like LpxC inhibitors[4] LpxA ELT hits should be able to potentiate the activity of teicoplanin. The MIC of teicoplanin against *A. baumannii* BM4652 is very poor at > 64 µg/ml, however in the presence of 32 µg/ml of **4**, the teicoplanin MIC dramatically decreases to 0.5 µg/ml (**Supplementary Table 4**). This complete potentiation of teicoplanin is consistent with inhibition of LPS/lipid A production by the LpxA inhibitor resulting in altered outer membrane permeability. Third, when LpxA overexpressor clone was introduced into *A. baumannii* BM4652, the MGIC of **4** was increased from 8 to > 128 µg/ml (**Supplementary Table 5**) and the concentration of compound **4** required to completely potentiate teicoplanin increased from 32 to >128 µg/ml (**Supplementary Table**

4). These results strongly suggest that the observed impaired growth and potentiation of teicoplanin by compound **4** are mediated though LpxA.

Supplementary Note 2: Antibacterial mechanism of action of compound 5.

The on-target MoA of UppS compound **5** was investigated using an *A. baumannii* UppS overexpressor strain. In these studies, polymyxin B nonapeptide (30 µg/ml) was added to permeabilise the cell and help with compound entry. The MIC of compound **5** without the permabilising agent was > 128 µg/ml. In the presence of permeabilising agent, the MICs of **5** were 0.25 and 2 µg/ml against the *A. baumannii* pRK415 and pRK415-UppS expressor strains respectively. Hence, the compound MIC increased 8-fold in the UppS overexpressing strain relative to the parent strain. In contrast, MICs of control antibiotics ciprofloxacin, ceftazidime and azithromycin with different mechanism of actions were unchanged (**Supplementary Table 6**). These data, again combined with *in vitro* biochemical inhibition data, are consistent with an antibacterial MoA of **5** being through the target of interest, UppS.

Supplementary Note 3: Antibacterial mechanism of action of compound 6.

Antimicrobial MoA of LolA compound **6** identified from *A. baumannii* campaign was investigated using a *lolA* antisense expressor in *E.coli*. This approach uses antisense induced from a plasmid construct, to titrate down the LolA target and possibly sensitize the cell to specific inhibition by LolA inhibitors. Compound **6** showed a 32–fold MIC decrease to *E. coli* efflux strain transformed with the *lolA* antisense plasmid compared to vector alone, and is consistent with an on target MoA. In contrast, there was little or no MIC effect on control antibiotics of different mechanisms of action with imipenem and ciprofloxacin while azithromycin produced a 4-fold decrease, illustrating the specificity of the antisense strain for determining LolA MoA. Results, shown in **Supplementary Table 7**, are consistent with a positive LolA MoA for compound **6** though further confirmation is required using a LolA biochemical assay such as that described[5].

<u>Supplementary Methods:</u>

Construct Cloning and Protein Expression:

All genes were synthesized according to sequences available in the NCBI public database or from the genome of *A. baumannii* BM4454 that was completely sequenced in house, and cloned into either pCOLD vector for cytoplasmic proteins, or pBAD vector for membrane proteins. The pCOLD vectors contain a N-terminal Flag tag and a C-terminal SBP tag. The pBAD vectors contain a C terminal SBP-Flag tag. *E. coli* BL21(DE3) cells were transformed with recombinant plasmid (pCOLD or pBAD). For pCOLD expression, a single colony was inoculated into 5 mL of Luria-Bertani (LB) medium containing 100 µg/mL ampicillin. The cells were incubated at 37°C, and shaken at 180 rpm overnight. Cultures were diluted 1:50 and expression was initiated with the addition of 0.1 mM IPTG at OD_{600}=0.6-0.8. Expression was carried out at 16°C for approximately 20 hrs. Cells were harvested by centrifugation. For pBAD expression, a single colony was inoculated into 5 mL of LB medium containing 100 µg/mL ampicillin + 34 µg/ml chloramphenicol. The cells were incubated at 37°C with shaking at 180 rpm overnight. Cultures were diluted 1:50 and expression was initiated with the addition of 0.2 % L-arabinose at OD_{600}=0.6-0.8. Expression was carried out at 16°C for approximately 20 hrs. Cells were harvested by centrifugation. Expression was evaluated using Western blot with anti-Flag or anti-His antibodies. Cloning of *A. baumannii* targets and their protein overexpression and production were outsourced to GenScript.

Construction of MRS & UppS overexpressor in S.aureus

S. aureus strains RN4220 (pYH4) and RN4220 (pYH4-UppS) and RN4220 (pYH4-MRS) were from an ORF expression library of the genome of *S. aureus* and ORF overexpression was induced by 0.1 µg/ml of anhydrotetracycline[1,2].

Construction of LpxA and UppS overexpressor in A. baumannii

The ORFs of *lpxA* & *uppS* were PCR amplified from *A. baumannii* BM4454 genomic DNA and cloned into pCR™-Blunt II-TOPO® using the ZeroBlunt® TOPO® PCR Cloning Kit (Life Technologies). The following primers were used in the PCR amplification, which include unique restriction sites (in italic) and an *E. coli* consensus ribosome binding site (RBS, in bold): AbLpxAF, 5'-CGC*TCTAGA***GAAGGA**GATAAGGCATGAGCAATCACGATTTAATC-3', AbLpxAR, 5'-CGC*GAGCTC*TTAGCGCACAATTCCACG-3'and AbuppSF, 5'-GCC*AAGCTT***GAAGGA**GATAAACCATGACCGATTCAGA-3', ABuppSR 5'–GCCG*TCTAGA*TTATAATTTCTCGATTTTCTCTTGCTG-3'. The resultant clones were sequenced to confirm the ORF identity and to be free of errors, digested with the unique restriction enzymes and subcloned into pRK415[8]. The pRK415-*lpxA* or pRK415-*uppS* plasmids were electroporated into *A. baumannii* BM4652 (efflux deletion strain) followed by selection on Mueller-Hinton (MH) agar plates containing 4 µg/ml of

tetracycline. The transcription of pRK415-lpxA and pRK415-uppS is from the P_{lac} promoter on pRK415 and translation uses the *E. coli* RBS.

Construction of LolA antisense expressor in E. coli

Primers AC**GGCGCGCC**GGGAGTGACGTAATTTGAGGA and TT**GTTTAAAC**GGCTTTTCAGATCGCTTGCG (introducing unique restriction sites *Pme*I and *Asc*I in bold on the respective primers) were used to PCR amplify from *E. coli* TOP10 genomic DNA, a DNA fragment complementary to 25 bp of an upstream region encompassing the *lolA* ribosome binding site (rbs) and 89 bp of the N-terminal region of the *E. coli lolA* gene. The PCR product was digested with *Pme*I and *Asc*I, ligated into similarly cut pHN678', a vector with an IPTG-inducible promoter modified to include new *Pme*I and *Asc*I cloning sites, and transformed into *E. coli* TOP10 competent cells (Invitrogen) with selection on chloramphenicol (5 µg/ml)[9]. Plasmid isolated from a single colony was confirmed by DNA sequencing to contain *lolA* in an antisense orientation relative to the IPTG promoter. The construct was transformed into *E. coli* TOP10 tolC (an efflux knockout mutant strain). In mode-of action studies, MIC plates were set up containing test compound in MH broth supplemented with 0.125 mM of IPTG to induce expression of *lolA* antisense and specifically sensitize the *E. coli* tolC *lolA* antisense expressor cells to LolA inhibitors. 7.5 µg/ml of polymyxinB nonapeptide was added to permeabilize cells to compound. Cells were incubated at 37°C and growth monitored over 48 h.

***In vitro* Biochemical Assay Testing:**

t-RNA synthetase biochemical assay (IRS, MRS)

A modified protocol from Kumar, R. *et. al.* was used to measure *S. aureus* t-RNA synthetase activity[10]. Compounds were tested using an 11 point dose-response to measure an IC_{50}. Briefly, 2-2.5 nM enzyme (IRS or MRS) was added to reaction plate containing inhibitor. All reagents were added using a Matrix multichannel 1250 µl pipette. The reaction was initiated with the addition of substrate mix which contained: 25 µM (0.005 µCi/µL) ^{14}C-isoleucine, or 0.5 µM (0.04 µCi/µL) ^{3}H-methionine with 1 mg/ml *E. coli* tRNA in assay buffer with 50 mM Tris-HCl, pH 7.9, 10 mM $MgCl_2$, 50 mM KCl, 2 mM DTT and 0.1 mg/ml BSA. The reaction was incubated for 30 min at room temperature. After the incubation, the reaction was terminated by transferring 35 µL into filter plate containing 100 µL of 10% trichloro acetic acid (TCA). The plate was filtered and the filter washed 3 times with 100 µL of 10% TCA. The filter plate (Multiscreen HV filter plate (0.45µM)(cat# MSHVN45B50) was dried in 60°C oven for 1h, and read in Topcount after adding 50 µL of MicroScint cocktail. Compounds were tested at 1% DMSO in 11 point dose response and fit to a standard 4 parameter fit to calculate an IC_{50} value and are reported as the average of two replicates. Standard deviation values were calculated using the n-1 method.

UppS biochemical assay

Both the *S. aureus and A. baumannii* UppS *in vitro* biochemical assay use a pyrophosphatase and Biomol Green Phosphate detection reagent to assess catalytic activity. These assays were based on previously described assays both within GSK and others[11-13]. Briefly, the enzyme will add up to 8 isopentyl pyrophosphate (IPP) units onto a farnesyl pyrophosphate FPP molecule, resulting in the production of 8 moles of inorganic pyrophosphates per mole of substrate added. The pyrophosphate is then converted to inorganic phosphate by a pyrophosphatase, and this phosphate is detected by Biomol Green. The assay is conducted in buffer containing 100 mM Trizma pH7.5, 1 mM $MgCl_2$, 6 mM CHAPS and 0.005% bovine serum albumin (BSA). 5 nM UppS, 1 µM FPP, 10 µM IPP and 0.5 U/ml pyrophosphatase were incubated for 30 min at room temperature followed by an equal volume addition of Biomol Green reagent. All reagents were added using a Multidrop combi (Thermo Scientific) to a final assay volume of 10 µL. The detection reagent was incubated for 20 min and 610-620 nm light was monitored by a Perkin Elmer Envision plate reader. Assay plates (384-well Corning plate # 3540) were pre-dispensed with 100 nL DMSO or with 100 nL compounds dissolved in neat DMSO using an Echo® liquid handler (Labcyte Inc.). Compounds were tested at 1% DMSO in 11 point dose response with a 1:3 dilution and fit to a standard 4 parameter fit to calculate an IC_{50} value and are reported as the average of two replicates. Standard deviation values were calculated using the n-1 method.

MetAP biochemical assay

A fluorescence intensity coupled assay was used to measure the *in vitro* biochemical activity of *S. aureus* methionine aminopeptidase, MetAP. A 7-amino-trifluoromethylcoumarin (AFC) labeled peptide with the amino acid sequence MGFGF-AFC is converted to GFGF-AFC by MetAP. A cathepsin C coupling enzyme then digests the liberated peptide GFGF-AFC releasing the AFC causing a fluorescence increase monitored by an Envision plate reader. The assay was conducted in 50 mM HEPES (pH 7.5), 100 mM NaCl, 0.5 mM CHAPS, 200 µM $NiCl_2$, 2.5 mM glutathione. 50 nM MetAP and 8 µM peptide are incubated at 25°C for 1 hr before quenching with a 1 mM 1,10-phenanthroline, 15 nM cathepsin C solution. After 90 minute incubation with quench/detection solution, plates were read on an envision plate reader with an excitation wavelength of 405 nm, emission wavelength of 530 nm, and a dichroic filter of 505 nm. All reagents were added using a Multidrop combi (Thermo Scientific) to a final assay volume of 10 µL. Assay plates (384 Black Greiner Catalog # 784075) were pre-dispensed with 100 nL DMSO or with 100 nL compounds dissolved in neat DMSO using an Echo® liquid handler (Labcyte Inc.). Compounds were tested at 1% DMSO in 11 point dose response with a 1:3 dilution and fit to a standard 4 parameter fit to calculate an IC_{50} value and are reported as the average of two replicates. Standard deviation values were caluculated using the n-1 method.

Dihydrofolate reductase assay

A fluorescence intensity coupled assay was used to measure the *in vitro* biochemical activity of *M. tuberculosis* Dihydrofolate reductase (DHFR). DHFR catalyzes the reduction of dihydrofolate (DHF) to tetrahydrofolate (THF) using NADPH as a cofactor. A diaphorase coupling enzyme then uses the

remaining NADPH to convert resazurin into the fluorescent resorufin[14]. The assay buffer containing 81.4 mM Hepes pH 7.8, 300 mM KCl, 0.4 mg/mL BSA was used to make the enzyme addition containing 1.2 µg/mL Mtb DHFR and 50 µM NADPH. This was added in equal volume (5 µL) to a substrate solution composed of 240 µM DHF in H_2O and incubated for 50 min. Developing solution was prepared with 0.045 mM Resazurin and 0.6 U/ml Diaphorase in 200 mM sodium phosphate buffer pH 7.8 and 5 µL was added. The experiments were conducted in black 384-well plates (Greiner Catalog # 784076) and all liquid additions were conducted using a Multidrop combi (Thermo Scientific). The plate was read on a ViewLux following a 10 minute delay. Resorufin light production was measured using appropriate Viewlux filters: Ex: 525 / 20 Pol (BODIPY TMR FP) Em: 598 / 25 (BODIPY TMR) (B04). Assay plates were pre-dispensed with 100 nL DMSO or with 100 nL compounds dissolved in neat DMSO using an Echo® liquid handler (Labcyte Inc.) where all wells contained compound samples, except wells in columns 6 and 18 that contained DMSO control. Column 6 represented 100% Mtb DHFR activity in the absence of GSK compounds. Column 18 represented 100% inhibition in absence of Mtb DHFR. Compounds were tested at 1% DMSO in 11 point dose response with a 1:3 dilution and fit to a standard 4 parameter fit to calculate an IC_{50} value and are reported as the average of two replicates. Standard deviation values were calculated using the n-1 method.

Minimum Inhibitory Concentration Assays:

MIC determination against M. tuberculosis H37Rv:

The measurement of the Minimum Inhibitory Concentration (MIC) against *M. tuberculosis* H37Rv for each tested compound was performed in 96-well flat-bottom, polystyrene microtiter plates in a final volume of 100 µl. Ten two-fold drug dilutions in neat DMSO starting at 50 mM were performed. Drug solutions were added to Middlebrook 7H9 medium (Difco) and Isoniazid (INH) (Sigma Aldrich) was used as a positive control with two-fold dilutions of INH starting at 160 µg/ml. The inoculum was standardized to approximately $1x10^7$ cfu/ml and diluted 1 in 100 in Middlebrook 7H9 broth (Difco). This inoculum (100 µl) was added to the entire plate but G-12 and H-12 wells were used as blank controls. All plates were placed in a sealed box to prevent drying out of the peripheral wells and incubated at 37ºC without shaking for six days. A Resazurin solution was prepared by dissolving one tablet of resazurin (Resazurin Tablets for Milk Testing; Ref 330884Y' VWR International Ltd) in 30 ml of sterile PBS (phosphate buffered saline). Of this solution, 25 µl were added to each well. Fluorescence was measured (Spectramax M5 Molecular Devices, Excitation 530nm, Emission 590 nm) after 48 hours to determine the MIC_{90} value. The values reported were observed in a minimum of 2 replicates.

MIC determination against all other bacteria:

Minimum inhibitory concentrations (MICs) of compounds were determined using broth microdilution methods according to Clinical and Laboratory Standards Institute guidelines[15,16]. The MIC was the lowest concentration of an antibacterial that showed no visible growth after incubation at 37 °C for 18–24 h,

with a starting inoculums of ~5.5×10^5 colony forming units per mL. Bacterial strains used were from GSK's culture collection. All compound MICs are representative of at least two independent experiments and were within acceptable two-fold variation range.

Compound Synthesis:

General Methods: Commercially available starting reagents for the synthesis were purchased from Sigma-Aldrich and Fisher Scientific and used without further purification. Purification of final compounds for biological testing was performed on a Gilson GX-281 system with a Phenomenex Luna 5µ C8(2) 100X30 mm 100A column running gradient of 10-80% acetonitrile (ACN)/H_2O (+0.1% trifluoroacetic acid [TFA]) over 20 minutes with flow rate of 35mL/min. The purity of final compounds was checked using an Agilent 1100 HPLC system coupled with a Thermo Finnigan LCQ Mass Spectrometer – Phenomenex Luna 3µ C8(2) 100A 50 x 3.00 mm column running gradient of 10-95% ACN/H_2O (+0.1% formic acid) over 15 minutes with flow rate 0.5mL/min. ^1H and ^{13}C NMR spectra were recorded on a Varian Mercury 400 plus (^1H at 400.2 MHz and ^{13}C at 100.6MHz) or on a Bruker Avance instrument equipped with a TCI cryoprobe Plus (^1H at 600.1 MHz and ^{13}C at 150.9MHz). Chemical shifts are expressed in parts per million (ppm, δ units). Coupling constants (*J*) are in units of Hertz (Hz). Splitting patterns describe apparent multiplicities and are designated as s (singlet), d (doublet), t (triplet), q (quartet), dd (double doublet), dt (double triplet), m (multiplet). High Resolution mass spectrometry was measured either on the Thermo LTQ Orbitrap Discovery (SN01442B) operating in electrospray ionization, positive (ESI+) at a resolving power 30,000; or on the Thermo Exactive Plus (SN02078P) operating in electrospray ionization, negative (ESI-) at a resolving power 35,000. The system is calibrated using Thermo's positive ion calibration mix (caffeine, MRFA, and Ultramark polymer) on a weekly basis. Sample is introduced in 50:50 water: ACN having 0.05% TFA at 250 µL/min by an Agilent 1200 LC to the MS Iontrap-Orbitrap, or by an Agilent 1100 LC to the MS Orbitrap.

Synthesis of 2-(4-bromo-3-hydroxyphenyl)-1-(2-(2-(2-(*tert*-butyl)phenoxy)acetamido)-1-(3,4-dimethoxyphenyl)ethyl)-*N*-methyl-1*H*-benzo[*d*]imidazole-5-carboxamide (Compound 1)

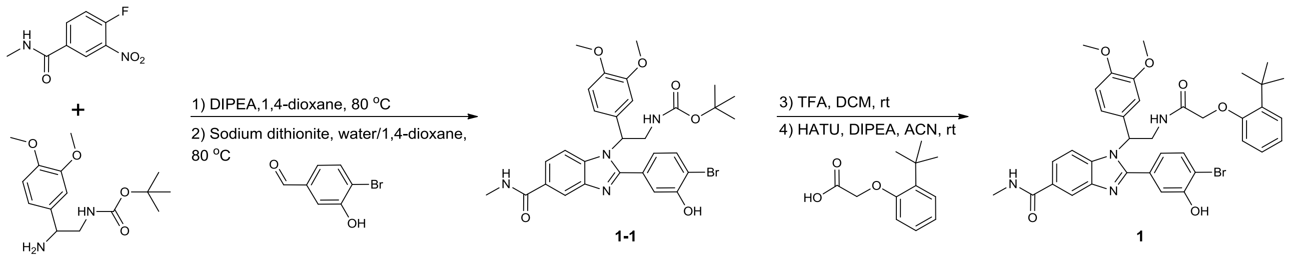

To a solution of 4-fluoro-*N*-methyl-3-nitrobenzamide (141 mg, 0.712 mmol) and *tert*-butyl (2-amino-2-(3,4-dimethoxyphenyl)ethyl)carbamate (211 mg, 0.712 mmol) in 1,4-dioxane (3 mL) was added *N*,*N*-diisopropylethylamine (DIPEA) (0.173 mL, 0.997 mmol) at room temperature. The reaction was heated at 80 °C for 8 hours. To the reaction was then added sodium dithionite (372 mg, 2.136 mmol), 4-bromo-3-hydroxybenzaldehyde (143 mg, 0.712 mmol) and water (0.75 mL). The reaction was heated at 80 °C for 48 hours. The reaction was concentrated under vacuum and the residue was purified by a reverse phase HPLC to give the desired product **1-1** (240 mg, 54% yield). ^{1}H NMR (400MHz, DMSO-d6): δ 10.76 (br s, 1H), 8.45 (m, 1H), 8.17 (s, 1H), 7.68 (dd, 1H, *J*=8.6, 1.6Hz), 7.64 (m, 1H), 7.29 (m, 1H), 7.23 (m, 1H), 6.99-6.76 (m, 4H), 5.79 (br s, 1H), 4.01 (m, 1H), 3.80 (m, 1H), 3.71 (s, 3H), 3.66 (s, 3H), 2.78 (d, 3H, *J*=4.7Hz), 1.19 (s, 9H); ^{13}C NMR (101MHz, DMSO-d6): δ 167.1, 155.9, 154.9, 154.8 149.2, 149.1, 148.9, 135.7, 133.7, 128.9, 122.7, 121.4, 119.5, 117.9, 117.6, 113.4, 112.5, 112.3, 111.4, 110.8, 110.0, 78.5, 59.5, 56.0, 55.9, 28.6, 28.4, 26.8; MS (ESI+) (*m/z*): $[M+H]^+$ calcd. for $C_{30}H_{34}BrN_4O_6$, 625.16; found, 624.75.

To a solution of **1-1** (200 mg, 0.320 mmol) in dichloromethane (DCM) (2 mL) was added TFA (0.246 mL, 3.20 mmol) at room temperature. The reaction was stirred at room temperature for 12 h and then concentrated. The residue was re-dissolved in ACN (1 mL) and added to a solution of 2-(2-(*tert*-butyl)phenoxy)acetic acid (33.3 mg, 0.160 mmol) and 1-[bis(dimethylamino)methylene]-1*H*-1,2,3-triazolo[4,5-*b*]pyridinium 3-oxide hexafluorophosphate (HATU) (73.0 mg, 0.192 mmol) in ACN (2 mL) at room temperature. The reaction was stirred at room temperature for 2 hours and concentrated. The residue was purified by a reverse phase HPLC to give the desired product **1** (46 mg, 39% yield). ^{1}H NMR (600MHz, DMSO-d6): δ 10.75 (br s, 1H), 8.45 (br d, 1H, *J*=4.2 Hz), 8.22 (d, 1H, *J*=1.1 Hz), 8.05 (t, 1H, *J*=5.9 Hz), 7.73 (br d, 1H, *J*=8.7 Hz), 7.65 (d, 1H, *J*=7.9 Hz), 7.45 (d, 1H, *J*=8.3Hz), 7.25 (s, 1H), 7.19 (dd, 1H, *J*=7.7, 1.3 Hz), 7.07 (t, 1H, *J*=7.8 Hz), 6.99 (dd, 1H, *J*=8.1, 1.7 Hz), 6.92 (d, 1H, *J*=8.3 Hz), 6.88 (d, 1H, *J*=2.3 Hz), 6.84-6.87 (m, 1H), 6.82 (br d, 1H, *J*=8.3 Hz), 6.61 (d, 1H, *J*=8.3 Hz), 5.86 (dd, 1H, *J*=9.1, 5.7 Hz), 4.35-4.40 (m, 1H), 4.31 (q, 2H, *J*=17.0 Hz), 4.03-4.05 (m, 1H), 3.73 (s, 3H), 3.68 (s, 3H), 2.81 (d, 3H, *J*=4.5 Hz), 1.24 (s, 9H); ^{13}C NMR (151MHz, DMSO-d6): δ 168.1, 166.5, 156.7, 154.5, 154.4, 148.8, 148.5, 141.2, 137.6, 135.1, 133.4, 129.4, 128.4, 127.1, 126.3, 122.3, 121.2, 120.9, 119.0, 117.9, 117.0, 113.1, 112.8,

112.0, 111.8, 110.4, 67.1, 58.4, 55.5, 55.5, 39.9, 34.3, 29.7, 26.3; HRMS (*m/z*): $[M+H]^+$ calcd. for $C_{37}H_{40}BrN_4O_6$, 715.2126; found, 715.2115.

Synthesis of 3-(((3-(*tert*-butyl)-5-(4-(3,5-dichlorophenyl)piperazine-1-carbonyl)benzyl)amino)methyl)-*N*-methylbenzamide (Compound 2)

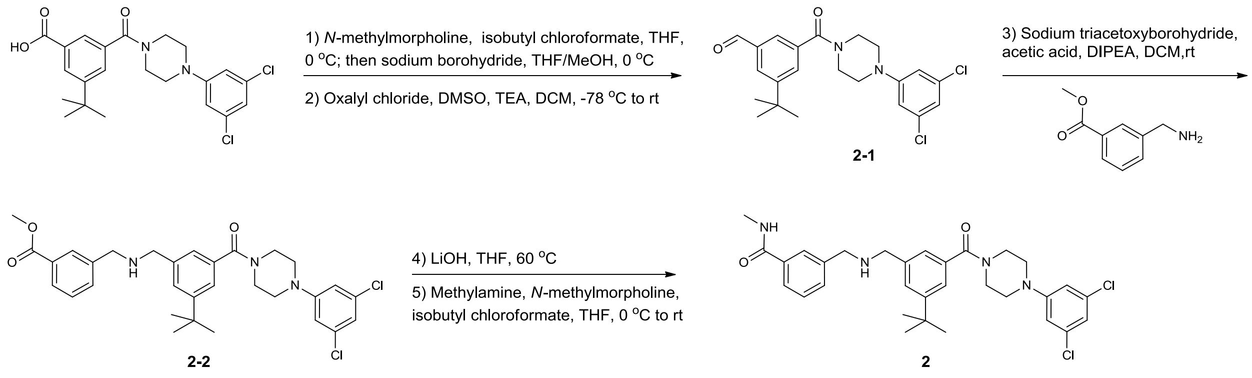

To a solution of 3-(*tert*-butyl)-5-(4-(3,5-dichlorophenyl)piperazine-1-carbonyl)benzoic acid (106 mg, 0.243 mmol) and *N*-methylmorpholine (0.027 mL, 0.243 mmol) in tetrahydrofuran (THF) (2 mL) at 0 °C was added dropwise a solution of isobutyl chloroformate (0.032 mL, 0.243 mmol) in THF (1 mL). The reaction mixture was then stirred at the same temperature for 15 minutes at which time the reaction mixture was added dropwise to a solution of sodium borohydride (9.19 mg, 0.243 mmol) in a 3:1 mixture of THF (3 mL) and methanol (1 mL). After 30 minutes the reaction was quenched with 10% acetic acid/H_2O. The reaction was then concentrated. The residue was taken up in ethyl acetate and washed with dilute $NaHCO_3$ (2×) and brine. It was dried over $MgSO_4$ and then concentrated. To a solution of oxalyl chloride (0.024 mL, 0.279 mmol) in DCM (1mL) at -78 °C was added dimethyl sulfoxide (DMSO) (0.040 mL, 0.559 mmol) dropwise. The reaction mixture was then stirred at the same temp for 10 minutes at which time the residue from the previous step in DCM (1mL) was added. The reaction was continued to stir at the same temperature for 10 minutes at which time triethylamine (0.177 mL, 1.27 mmol) was added dropwise to the reaction mixture. After stirring at -78 °C for 5 more minutes the cold bath was removed and the reaction mixture was allowed to warm to room temperature. The reaction mixture was concentrated and purified with a reverse phase HPLC to obtain the desired product **2-1** (70.5 mg, 69% yield). ^1H NMR (400MHz, $CDCl_3$): δ 10.05 (s, 1H), 8.01 (t, 1H, *J* = 1.8Hz), 7.74 (d, 2H, *J* = 2Hz), 6.88 (t, 1H, *J* = 1.8Hz), 6.77 (d, 2H, *J* = 1.6Hz), 3.97 (m, 2H), 3.62 (m, 2H), 3.33 (m, 2H), 3.20 (m, 2H), 1.38 (s, 9H); ^{13}C NMR (101MHz, $CDCl_3$): δ 191.7, 170.3, 153.6, 152.0, 136.4, 135.7, 135.0, 130.4, 128.6, 125.3, 120.2, 114.6, 52.2, 50.1, 35.1, 31.1; MS (ESI+) (*m/z*): $[M+H]^+$ calcd. for $C_{22}H_{25}Cl_2N_2O_2$, 419.12; found, 419.15.

To a solution of **2-1** (84.9 mg, 0.202 mmol) in DCM (4mL) was added methyl 3-(aminomethyl)benzoate (61.2 mg, 0.304 mmol) and DIPEA (0.035 mL, 0.202 mmol) at room temperature. The reaction was stirred at room temperature for 5 minutes at which time acetic acid (0.4

mL) was added. The reaction was continued to stir at room temperature for an additional 15 minutes, and then sodium triacetoxyborohydride (86 mg, 0.405 mmol) was added and the reaction was allowed to stir at room temperature overnight. The reaction was quenched with a small amount of methanol and washed with water. The aqueous layer was separated and back extracted with DCM (1x). The combined organic layers were then washed with brine, dried over $MgSO_4$, and concentrated to obtain the desired product **2-2** (90 mg, 78% yield) which was used without any further purification. ^{1}H NMR (400MHz, $CDCl_3$): δ 8.04 (s, 1H), 8.02 (d, 1H, *J* = 1.6Hz), 7.56 (d, 1H, *J* = 8Hz), 7.47 (m, 1H), 7.44 (m, 1H), 7.40 (t, 1H, *J* = 1.6Hz), 7.30 (s, 1H), 6.87 (t, 1H, *J* = 1.8Hz), 6.75 (d, 2H, *J* = 2Hz), 4.10 (m, 4H), 3.91 (m, 2H), 3.88 (s, 3H), 3.53 (m, 2H), 3.26 (m, 2H), 3.16 (m, 2H), 1.25 (s, 9H); ^{13}C NMR (101MHz, $CDCl_3$): δ 170.6, 166.2, 153.5, 152.0, 135.6, 135.2, 134.2, 131.1, 130.9, 130.8, 130.7, 130.5, 129.4, 129.1, 125.2, 125.1, 120.0, 114.5, 52.3, 50.3, 50.1, 48.8, 42.1, 34.9, 30.9; MS (ESI+) (*m/z*): $[M+H]^+$ calcd. for $C_{31}H_{36}Cl_2N_3O_3$, 568.21; found, 568.20.

The residue was re-dissolved in THF (1 mL) and to it was added 1M LiOH aqueous solution (1 mL) and stirred at 60 ^{o}C for 2 hours. The reaction was acidified with 1N HCl and then diluted with ethyl acetate and washed with water (2×). The combined aqueous layers were back-extracted with ethyl acetate. The combined organic layers were then washed with brine and concentrated. The residue (39.7 mg, 0.072 mmol) was dissolved in THF (1.5 mL) and added *N*-methylmorpholine (9.84 µL, 0.089 mmol) at 0 ^{o}C, and it was then added a solution of isobutyl chloroformate (0.012 mL, 0.089 mmol) in THF (1 mL) dropwise. The reaction mixture was allowed to stir at the same temperature for 20 minutes at which time a solution of methylamine (6.7 mg, 0.215 mmol) in THF (0.5 mL) was added. The reaction was continued to stir at 0 ^{o}C for 5 minutes and then allowed to warm to room temperature. The reaction was continued to stir at room temperature for 3 days. The reaction was concentrated and set-up again with isobutyl chloroformate (0.006 mL, 0.045 mmol) and methylamine (6.7 mg) and continued for another 30 minutes. The reaction mixture was concentrated and the residue was purified by a reverse phase HPLC to obtain the desired product **2** (4.1 mg, 10% yield) ^{1}H NMR (600MHz, DMSO-d6): δ 9.31 (br s, 2H), 8.40-8.58 (m, 1H), 8.01 (s, 1H), 7.86 (d, 1H, *J*=7.6 Hz), 7.63 (d, 1H, *J*=7.0 Hz), 7.62 (s, 1H), 7.53 (t, 1H, *J*=7.9 Hz), 7.48 (s, 1H), 7.38 (s, 1H), 6.97 (s, 2H), 6.91 (s, 1H), 4.23-4.29 (m, 4H), 3.74 (br d, 2H, *J*=0.8 Hz), 3.40-3.47 (m, 2H), 3.22-3.32 (m, 4H), 2.79 (d, 3H, *J*=4.5 Hz), 1.32 (s, 9H) ; ^{13}C NMR (151MHz, DMSO-d6): δ 168.8, 166.1, 152.3, 151.5, 135.7, 134.9, 134.7, 132.6, 132.1, 132.0, 129.4, 128.6, 128.1, 127.2, 125.8, 124.5, 117.4, 113.4, 50.2, 50.2, 47.4, 41.2, 34.7, 30.9, 26.3; HRMS (*m/z*): $[M+H]^+$ calcd. for $C_{31}H_{37}Cl_2O_2N_4$, 567.2281; found, 567.2275.

Synthesis of 2-(1-(benzo[*d*][1,3]dioxol-5-yl)propan-2-yl)-7-chloro-1-((5-methyl-4*H*-1,2,4-triazol-3-yl)methyl)-1*H*-benzo[*d*]imidazole (Compound 3)

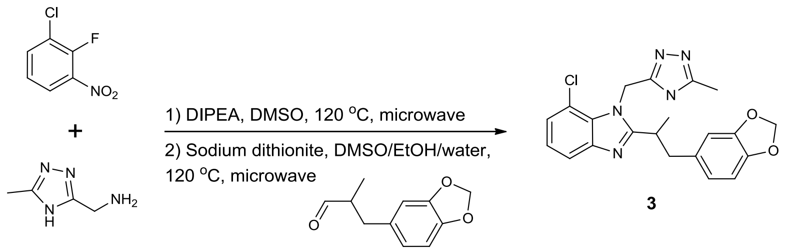

To a microwave reaction vial (5 mL) was added 1-chloro-2-fluoro-3-nitrobenzene (39.9 mg, 0.227 mmol), (5-methyl-4*H*-1,2,4-triazol-3-yl)methanamine HCl (46.3 mg, 0.250 mmol), DMSO (1 mL), and DIPEA (0.040 ml, 0.227 mmol). The reaction vial was sealed and heated in a microwave reactor at 120 °C for 15 minutes. To the reaction vial was added 3-(benzo[*d*][1,3]dioxol-5-yl)-2-methylpropanal (48.1 mg, 0.250 mmol) and sodium dithionite (79 mg, 0.455 mmol), followed by the addition of ethanol (0.5 mL) and water (0.5 mL). The reaction vial was sealed and heated in a microwave reactor at 120 °C for 20 minutes. The reaction mixture was purified with a reverse phase HPLC to give the desired product **3** (15 mg, 14% yield). ^{1}H NMR (600MHz, DMSO-d6): δ 13.49 (br s, 1H), 7.59 (d, 1H, *J*=7.7 Hz), 7.21 (d, 1H, *J*=7.7 Hz), 7.18 (t, 1H, *J*=7.6 Hz), 6.75 (br d, 1H, *J*=7.9 Hz), 6.74 (s, 1H), 6.59 (d, 1H, *J*=7.9 Hz), 5.94 (d, 2H, *J*=4.9 Hz), 5.70 (q, 2H, *J*=17.4 Hz), 3.47-3.54 (m, 1H), 2.99 (dd, 1H, *J*=13.6, 6.4 Hz), 2.76 (dd, 1H, *J*=13.4, 8.1 Hz), 2.26 (s, 3H), 1.20 (d, 3H, *J*=6.8 Hz) ; ^{13}C NMR (151MHz, DMSO-d6): δ 160.6, 156.0, 153.5, 147.0, 145.4, 143.8, 133.2, 130.2, 123.6, 122.6, 121.9, 117.6, 115.2, 109.2, 107.9, 100.6, 41.6, 41.0, 32.7, 19.0, 11.6; HRMS (*m*/*z*): $[M+H]^+$ calcd. for $C_{21}H_{21}ClN_5O_2$, 410.1378; found, 410.1373.

Synthesis of 2-(3-mesitylureido)-5-(4-methylpiperazin-1-yl)-5-oxo-*N*-(5,6,7,8-tetrahydronaphthalen-1-yl)pentanamide (Compound 4)

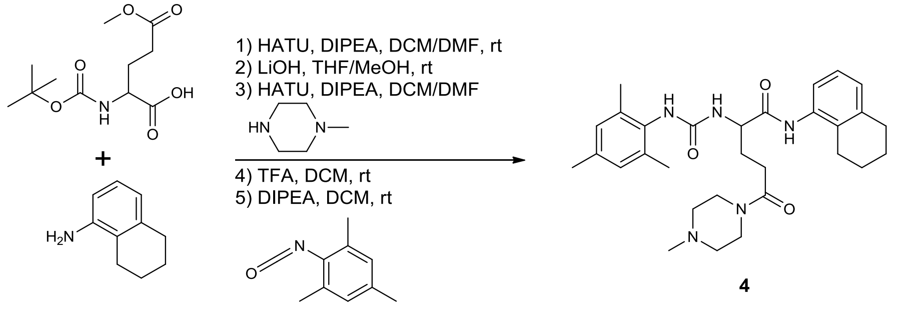

To a solution of 2-((*tert*-butoxycarbonyl)amino)-5-methoxy-5-oxopentanoic acid (78 mg, 0.300 mmol) in DCM (2 mL) was added a solution of HATU (137 mg, 0.360 mmol) in *N*,*N*-dimethylformamide (DMF) (1 mL), and this reaction was stirred at room temperature for 5 minutes at which point a solution of 5,6,7,8-tetrahydronaphthalen-1-amine (44.2 mg, 0.300 mmol) in DCM was added along with DIPEA

(0.157 ml, 0.9 mmol). The reaction was stirred an additional 5 hours at room temperature until complete acylation was observed by LCMS. Upon completion, the reaction was concentrated and then diluted with ethyl acetate and washed with $NaHCO_3$ aqueous solution (3×) followed by brine. The organic layer was collected and the solvent removed in vacuum. This crude material was then dissolved in THF (2 mL) and was added 2M LiOH aqueous solution (0.450 mL, 0.900 mmol) and methanol. The reaction was stirred at room temperature for 15 hours, at which point complete hydrolysis was observed by LCMS. Upon completion, the solvent was removed and ethyl acetate was added. The reaction was acidified with 1N HCl and then the organic layer was collected, washed with brine then dried over $MgSO_4$, and ethyl acetate was then removed. The residue was then dissolved in DCM (2 mL) and a solution of HATU (137 mg, 0.360 mmol) in DMF (1 mL) was added. After stirring at room temperature for 10 minutes, 1-methylpiperazine (30.0 mg, 0.300 mmol) along with DIEPA (0.157 mL, 0.900 mmol) was added. The reaction was stirred at room temperature for 2 hours at which point complete acylation was observed by LCMS. Upon completion the reaction was concentrated and then diluted with ethyl acetate and washed with $NaHCO_3$ aqueous solution (3×) followed by brine. The organic layer was collected and the solvent was removed. The residue was added to a solution of 20% TFA/DCM and stirred at room temperature for 3 hours, at which point complete deprotection of the Boc group was observed by LCMS. Upon completion the reaction was dried. The crude reaction material was dissolved in DCM (2 mL) and was added 2-isocyanato-1,3,5-trimethylbenzene and DIPEA (0.157 mL, 0.900 mmol). After stirring at room temperature for 3 hours complete urea formation was observed by LCMS. Upon completion the reaction was dried and the crude material was purified by a reverse phase HPLC to give the desired product **4** (26 mg, 13% yield). ^{1}H NMR (600MHz, DMSO-d6): δ 9.28 (s, 1H), 7.66 (br s, 1H), 7.25 (d, 1H, *J*=7.6 Hz), 6.98-7.15 (m, 1H), 6.91 (d, 1H, *J*=7.6 Hz), 6.85 (s, 2H), 6.59 (br s, 1H), 4.35-4.53 (m, 2H), 3.88-4.07 (m, 1H), 3.36-3.43 (m, 3H), 2.82-3.12 (m, 3H), 2.78 (br s, 3H), 2.73 (br t, 2H, *J*=5.5 Hz), 2.52-2.64 (m, 2H), 2.43-2.49 (m, 2H), 2.21 (s, 3H), 2.12 (s, 6H), 2.00-2.07 (m, 1H), 1.86 (dq, 1H, *J*=14.3, 7.3 Hz), 1.70-1.73 (m, 2H), 1.69 (br s, 2H); ^{13}C NMR (151MHz, DMSO-d6): δ 171.0, 170.4, 155.9, 137.4, 135.6, 135.3, 134.7, 133.1, 130.5, 128.3, 126.1, 125.1, 122.2, 53.1, 52.2, 42.3, 42.0, 38.3, 29.2, 28.7, 24.2, 22.4, 22.3, 20.4, 18.1; HRMS (*m/z*): $[M+H]^+$ calcd. for $C_{30}H_{42}N_5O_3$, 520.3282; found, 520.3280.

Synthesis of (*R*)-*N*1-(4-(*tert*-butyl)benzyl)-4,5-dichloro-*N*2-(3,3-dimethyl-1-(methylamino)-1-oxobutan-2-yl)phthalamide (Compound 5)

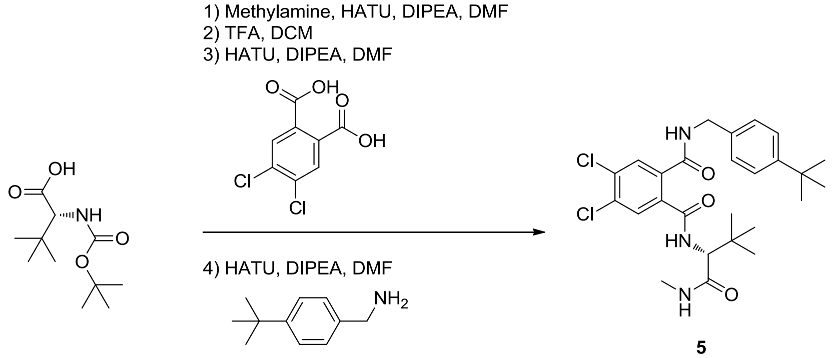

5

To a solution of (*R*)-2-((*tert*-butoxycarbonyl)amino)-3,3-dimethylbutanoic acid (100 mg, 0.432 mmol) and HATU (164 mg, 0.432 mmol) in DMF (2 mL) was added DIPEA (0.3 mL, 1.73 mmol) and the reaction was stirred at room temperature for 2 minutes before the addition of methylamine hydrochloride salt and the mixture was stirred at room temperature for 30 minutes. Ethyl acetate was added and washed with $NaHCO_3$ aqueous solution (3×) and brine. The organic layer was dried over Na_2SO_4 and concentrated. The residue was re-dissolved in a solution of 30% TFA/DCM (3 mL) and stirred at room temperature for 20 minutes. The solvent was removed under reduced pressure. The residue and HATU (164 mg, 0.432 mmol) was then added to a solution of 4,5-dichlorophthalic acid (102 mg, 0.432 mmol), (4-(*tert*-butyl)phenyl)methanamine (71 mg, 0.432 mmol), HATU (164 mg, 0.432 mmol) and DIPEA (0.3 mL, 1.73 mmol) in DMF (2mL) which had been stirred at room temperature for 30 minutes. The mixture was stirred at room temperature for 30 minutes and then purified with a reverse phase HPLC to give the desired product **5** (80 mg, 27% yield). ^{1}H NMR (600MHz, DMSO-d6): δ 8.98 (t, 1H, *J*=5.9 Hz), 8.33 (br d, 1H, *J*=8.7 Hz), 7.94 (br d, 1H, *J*=4.5 Hz), 7.77 (s, 1H), 7.69 (s, 1H), 7.33-7.36 (m, 2H, *J*=7.9 Hz), 7.26-7.28 (m, 2H, *J*=7.9 Hz), 4.37 (br d, 2H, *J*=5.7 Hz), 4.25 (d,1H, *J*=9.1 Hz), 2.58 (d, 3H, *J*=4.5 Hz), 1.27 (s, 9H), 0.96 (s, 9H) ; ^{13}C NMR (151MHz, DMSO-d6): δ 170.1, 165.8, 165.7, 149.2, 136.2, 135.7, 135.6, 132.0, 131.8, 130.3, 129.5, 127.1, 125.0, 61.0, 42.5, 34.1, 34.1, 31.2, 26.8, 25.3; HRMS (*m/z*): $[M+H]^+$ calcd. for $C_{26}H_{34}Cl_2N_3O_3$, 506.1972; found, 506.1971.

Synthesis of *N*-(2-(2-(3-cyano-5-fluorophenyl)-6-(ethylamino)pyrimidin-4-yl)-1-methyl-1,2,3,4-tetrahydroisoquinolin-6-yl)methanesulfonamide (Compound 6)

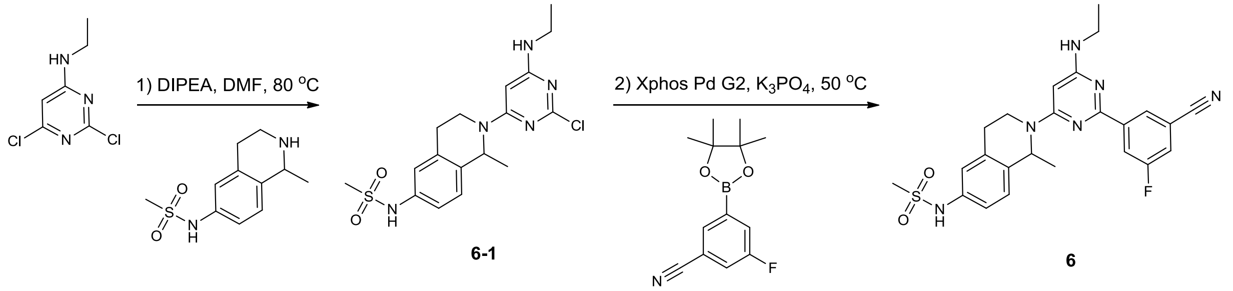

To a solution of 2,6-dichloro-*N*-ethylpyrimidin-4-amine (50 mg, 0.26 mmol) and *N*-(1-methyl-1,2,3,4-tetrahydroisoquinolin-6-yl)methanesulfonamide hydrochloride (72 mg, 0.26 mmol) in DMF (2 mL) was added DIPEA (0.135 mg, 1.04 mmol) and stirred at 80 °C for 1 day. The mixture was purified with a reverse phase HPLC to give the desired product **6-1** (48 mg, 47% yield). ^{1}H NMR (400MHz, DMSO-d6): δ 9.63 (s, 1H), 7.17 (d, 1H, *J* = 8.2Hz), 7.03 (dd, 1H, *J*= 8.4, 2.2Hz), 6.98 (d, 1H, *J*=2Hz), 6.15 (s, 1H), 3.35-3.18 (m, 5H), 2.94 (s, 3H), 2.79 (t, 2H, *J* = 5.7Hz) 1.37 (d, 3H, *J*= 6.7Hz), 1.08 (t, 3H, *J*= 7Hz); ^{13}C NMR (101MHz, DMSO-d6): δ 162.3, 161.1, 159.0, 158.6, 136.9, 135.6, 128.2, 120.1, 118.7, 90.9, 50.1, 35.9, 28.5, 21.4, 15.1. MS (ESI+) (*m/z*): $[M+H]^+$ calcd. for $C_{17}H_{23}ClN_5O_2S$, 396.12; found, 396.20.

To a solution of **6-1** (48 mg, 0.121 mmol) and 3-fluoro-5-(4,4,5,5-tetramethyl-1,3,2-dioxaborolan-2-yl)benzonitrile (64 mg, 0.26 mmol) in THF (2mL) and 0.5M K_3PO_4 aqueous buffer (1 mL) was added Xphos Pd G2 (12 mg, 0.016 mmol), and the mixture was stirred at 50 °C overnight. The

mixture was purified with a reverse phase HPLC to give the desired product **6** (4 mg, 6% yield). ^{1}H NMR (600MHz, DMSO-d6): δ 9.58 (s, 1H), 8.40 (s, 1H), 8.23 (br d, 1H, *J*=10.2 Hz), 7.86 (dd, 1H, *J*=8.1, 0.9 Hz), 7.15 (br d, 1H, *J*=8.3 Hz), 7.01 (br d, 1H, *J*=7.9 Hz), 6.95 (s, 1H), 6.77 (br s, 1H), 6.58-6.72 (m, 1H), 3.97 (s, 1H), 3.30 (br d, 2H, *J*=6.8 Hz), 2.90 (s, 3H), 2.78 (br s, 2H), 1.37 (d,3H, *J*=6.8 Hz), 1.09 (t, 3H, *J*=7.2 Hz); ^{13}C NMR (151MHz, DMSO-d6): 162.3, 161.9, 162.0, 159.0, 142.5, 136.4, 135.3, 134.5, 127.7, 126.7, 119.9, 119.7, 118.4, 118.2, 117.8, 112.9, 39.2, 35.4, 28.3, 21.0, 15.0; HRMS (*m*/*z*): [M+H]$^+$ calcd. for $C_{24}H_{26}FN_6O_2S$, 481.1816; found, 481.1827.

Synthesis of *N*-(3-amino-3-oxopropyl)-3-hydroxy-5-(2-(2-methyl-3-oxo-2*H*-benzo[*b*][1,4]oxazin-4(3*H*)-yl)acetamido)-*N*-(2-(4-methylphenylsulfonamido)ethyl)benzamide (Compound 7)

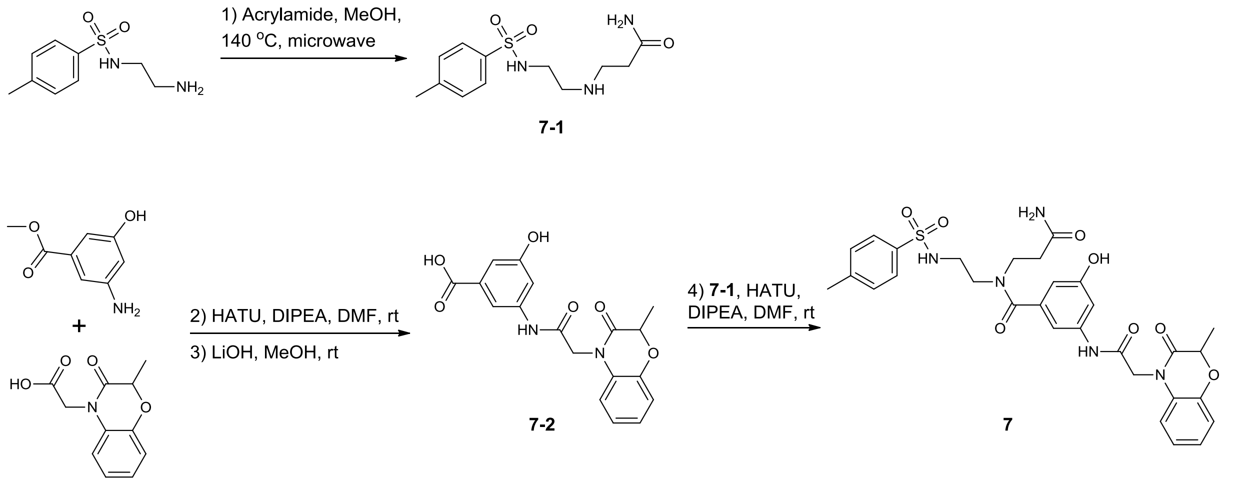

To a microwave vial was added a solution of *N*-(2-aminoethyl)-4-methylbenzenesulfonamide (2 g, 9.34 mmol) and acrylamide (331 mg, 4.67 mmol) in methanol (24 ml), and the vial was sealed and heated in a microwave reaction at 140 °C for 30 minutes. The reaction mixture was concentrated under reduced pressure and purified by a reverse phase HPLC to give the desired product **7-1** (952 mg, 90% yield). ^{1}H NMR (400MHz, DMSO-d6): δ 8.53 (br s, 2H), 7.84 (m, 1H), 7.69 (d, 2H, *J* = 8.2Hz), 7.41(d, 2H, *J* = 8.2Hz), 7.10 (br s, 1H), 3.08 (m, 2H), 2.97 (m, 4H), 2.44 (t, 2H, *J* = 8.0 Hz), 2.37 (s, 3H); ^{13}C NMR (101MHz, DMSO-d6): δ 171.9, 143.6, 137.0, 130.2, 127.01, 46.8, 43.6, 39.2, 30.8, 21.4; MS (ESI+) (*m*/*z*): [M+H]$^+$ calcd. for $C_{12}H_{20}N_3O_3S$, 286.11; found, 286.05.

To a solution of 2-(2-methyl-3-oxo-2*H*-benzo[*b*][1,4]oxazin-4(3*H*)-yl)acetic acid (50 mg, 0.22 6 mmol), methyl 3-amino-5-hydroxybenzoate (37.8 mg, 0.226 mmol) and HATU(86 mg, 0.226 mmol) in DMF (2 mL) was added DIPEA (0.16 mL, 0.9 mmol). The reaction was stirred at room temperature for 2 hours, and the solvent was then removed. The residue was suspended in methanol (3 mL) and LiOH 1M aqueous solution (1.1 mL, 1.1 mmol) was added. The solution was stirred at 50 °C for 2 hours and purified with a reverse phase HPLC to give the desired product **7-2** (65 mg, 72% yield). ^{1}H NMR (400MHz, DMSO-d6): δ 10.4 (s, 1H), 9.81 (br s, 1H), 7.61 (t, 1H, *J*=1.6Hz), 7.31 (t, 1H, *J*=2.2Hz), 7.01 (m, 5H), 4.76

(m, 3H), 1. 44 (d, 3H, *J* = 6.7Hz); ^{13}C NMR (101MHz, DMSO-d6): δ 167.5, 167.2, 166.0, 158.1, 144.2, 140.2, 132.6, 129.8, 124.1, 123.2, 117.2, 115.6, 111.6, 111.5, 110.7, 73.1, 44.9, 16.6; MS (ESI+) (*m/z*): $[M+H]^+$ calcd. for $C_{18}H_{17}N_2O_6$, 357.10; found, 356.69.

To a solution of **7-2** (22.6 mg, 0.063 mmol), **7-1** (18.1 mg, 0.063 mmol) and HATU (48.1 mg, 0.127 mmol) in DMF (1 ml) was added DIPEA (0.036 ml, 0.209 mmol). The reaction was stirred at room temperature for 16 hours and purified by a reverse phase HPLC to give the desired product **7** (6.7 mg, 17% yield). ^1H NMR (600MHz, DMSO-d6): δ 10.32 (br s, 1H), 9.74 (br s, 1H), 7.54 (br d, 3H, *J*=6.4 Hz), 7.25-7.45 (m, 3H), 7.13 (br s, 1H), 7.01-7.06 (m, 1H), 7.00 (br s, 1H), 7.00-7.05 (m, 1H), 6.99-7.06 (m, 1H), 6.89-6.99 (m, 1H), 6.82 (br d, 1H, *J*=11.7 Hz), 6.32-6.47 (m, 1H), 4.76-4.81 (m, 1H), 4.70 (br s, 2H), 3.49 (br s, 1H), 3.37-3.42 (m, 1H), 3.36-3.42 (m, 1H), 3.15-3.26 (m, 1H), 2.94 (br s, 1H), 2.78 (br s, 1H), 2.37 (br d, 3H, *J*=10.2 Hz), 2.31-2.37 (m, 1H), 2.22 (br s, 1H), 1.46 (d, 3H, *J*=6.4 Hz); ^{13}C NMR (151MHz, DMSO-d6): δ 172.1, 170.5, 166.7, 165.4, 157.6, 143.8, 142.6, 139.6, 138.1, 137.5, 129.6, 129.4, 126.4, 123.6, 122.7, 116.7, 115.2, 108.5, 107.7, 106.7, 72.7, 45.8, 44.5, 44.4, 40.5, 33.7, 20.9, 16.1; HRMS (*m/z*): $[M+H]^+$ calcd. for $C_{30}H_{34}N_5O_8S$, 624.2123; found, 624.2117.

Source of 6-(2,5-dimethoxybenzyl)-5-methylpyrido[2,3-d]pyrimidine-2,4-diamine (Compound 8)

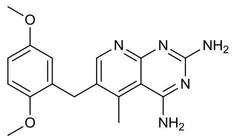

8

This compound was provided by an outsourcing company and was available through GSK corporate collection. Compound **8** is now available through commercial sources under CAS#72732-56-0 from Ark Pharma, Atomax Chemicals, BOC Sciences, and a few additional small vendors. Its structure was confirmed with the analytical data. ^1H NMR (600MHz, DMSO-d6): δ 8.34 (s, 1H), 6.92 (d, 3H, *J*=9.1 Hz), 6.76 (dd, 1H, *J*=8.7, 3.0 Hz), 6.42 (d, 1H, *J*=3.0 Hz), 6.19 (br s, 2H), 3.89 (s, 2H), 3.75 (s, 3H), 3.61 (s, 3H), 2.54 (s, 3H) ; ^{13}C (151MHz, DMSO-d6): δ 164.0, 161.5, 161.3, 156.0, 153.1, 151.0, 144.0, 129.2, 126.5, 115.9, 111.5, 111.0, 105.4, 55.8, 55.2, 30.2, 17.8; HRMS (*m/z*): $[M+H]^+$ calcd. for $C_{17}H_{20}O_2N_5$, 326.1612; found, 326.1605.

Synthesis of 3-(2-((6-methylpyridin-2-yl)amino)-2-oxoethyl)-1*H*-indole-2-carboxylic acid (Compound 9)

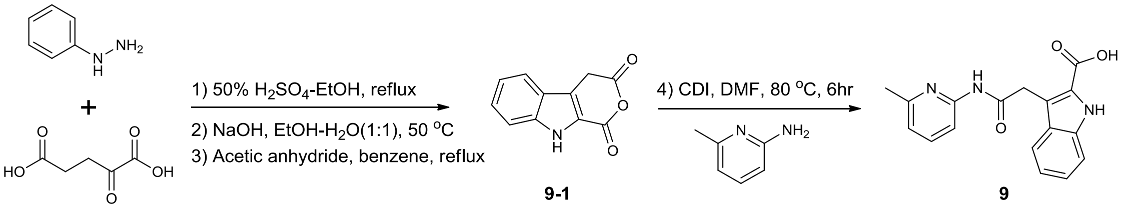

9-1 **9**

Intermediate **9-1** (200 mg, 1.0 mmol), which was prepared as previously reported[17], in anhydrous DMF (5 mL) was added 1,1'-carbonyldiimidazole (CDI) (182 mg, 1.15 mmol) and stirred at 80 °C for 1 hour. Then 6-methylpyridin-2-amine (151 mg, 1.4 mmol) was added and the mixture was stirred at 80 °C for 5 hours. The mixture was purified by a reverse phase HPLC to give the desired product **9** (186 mg, 60% yield). ^{1}H NMR (600MHz, DMSO-d6): δ 13.15 (br s, 1H), 11.59 (s, 1H), 10.39 (s, 1H), 7.82 (d, 1H, *J*=7.9 Hz), 7.65 (d, 1H, *J*=8.4Hz), 7.61 (t, 1H, *J*=7.8Hz), 7.42 (d, 1H, *J*=8.3Hz), 7.24 (t, 1H, *J*=7.6Hz), 7.05 (t, 1H, *J*=7.6 Hz), 6.93 (d, 1H, *J*=7.6 Hz), 4.21 (s, 2H), 2.40 (s, 3H); ^{13}C NMR (151MHz, DMSO-d6): δ 169.7, 163.5, 156.4, 151.4, 138.4, 135.9, 127.6, 125.3, 124.5, 120.3, 119.5, 118.4, 115.5, 112.4, 110.0, 32.6, 23.6; HRMS (*m/z*): $[M+H]^+$ calcd. for $C_{17}H_{16}N_3O_3$, 310.1186; found, 310.1180.

Supplementary References:

1 Ji, Y. *et al.* Validation of antibacterial mechanism of action using regulated antisense RNA expression in Staphylococcus aureus. *FEMS Microbiol Lett* **231**, 177-184 (2004).

2 Huang, J. *et al.* Novel chromosomally encoded multidrug efflux transporter MdeA in Staphylococcus aureus. *Antimicrob Agents Chemother* **48**, 909-917 (2004).

3 Moffatt, J. H. *et al.* Colistin resistance in Acinetobacter baumannii is mediated by complete loss of lipopolysaccharide production. *Antimicrob Agents Chemother* **54**, 4971-4977 (2010).

4 Tomaras, A. P. *et al.* LpxC inhibitors as new antibacterial agents and tools for studying regulation of lipid A biosynthesis in Gram-negative pathogens. *MBio* **5**, e01551-01514 (2014).

5 McLeod, S. M. *et al.* Small-molecule inhibitors of gram-negative lipoprotein trafficking discovered by phenotypic screening. *J Bacteriol* **197**, 1075-1082 (2015).

6 Concha, N. *et al.* Discovery and Characterization of a Class of Pyrazole Inhibitors of Bacterial Undecaprenyl Pyrophosphate Synthase. *J Med Chem* **59**, 7299-7304 (2016).

7 Payne, D. J., Gwynn, M. N., Holmes, D. J. & Pompliano, D. L. Drugs for bad bugs: confronting the challenges of antibacterial discovery. *Nat Rev Drug Discov* **6**, 29-40 (2007).

8 Keen, N. T., Tamaki, S., Kobayashi, D. & Trollinger, D. Improved broad-host-range plasmids for DNA cloning in gram-negative bacteria. *Gene* **70**, 191-197 (1988).

9 Nakashima, N. & Tamura, T. Conditional gene silencing of multiple genes with antisense RNAs and generation of a mutator strain of Escherichia coli. *Nucleic Acids Res* **37**, e103 (2009).

10 Islam, M. M., Pandya, P., Kumar, S. & Kumar, G. S. RNA targeting through binding of small molecules: Studies on t-RNA binding by the cytotoxic protoberberine alkaloid coralyne. *Mol Biosyst* **5**, 244-254 (2009).

11 Lee, L. V. *et al.* Biophysical investigation of the mode of inhibition of tetramic acids, the allosteric inhibitors of undecaprenyl pyrophosphate synthase. *Biochemistry* **49**, 5366-5376 (2010).

12 Chang, S. Y., Ko, T. P., Chen, A. P., Wang, A. H. & Liang, P. H. Substrate binding mode and reaction mechanism of undecaprenyl pyrophosphate synthase deduced from crystallographic studies. *Protein Sci* **13**, 971-978 (2004).

13 Chang, S. Y., Ko, T. P., Liang, P. H. & Wang, A. H. Catalytic mechanism revealed by the crystal structure of undecaprenyl pyrophosphate synthase in complex with sulfate, magnesium, and triton. *J Biol Chem* **278**, 29298-29307 (2003).

14 Kumar, A. *et al.* High-throughput screening and sensitized bacteria identify an M. tuberculosis dihydrofolate reductase inhibitor with whole cell activity. *PLoS One* **7**, e39961 (2012).

15 Barry, A. L. Standardization of antimicrobial susceptibility testing. *Clin Lab Med* **9**, 203-219 (1989).

16 (CLSI), C. a. L. S. I. Methods for dilution antimicrobial susceptibility tests for bacteria that grow aerobically; approved standard ninth edition, M07-A9, CLSI, Wayne, PA (2012).

17 Wucherpfennig, K., Nicholson, M., Xing, X., Stein, R., & Cuny, G. Preparation of 2-carboxy-3-indoleacetamides for enhancing MHC class II therapies. PCT Internation Application, WO 2008121836 A1 filed on Oct 09, 2008.
